# piR-26681 suppresses ovarian cancer progression by enhancing METTL3/METTL14-mediated m^6^A modification of FBXO16 and impairing DNA repair via MORF4L1 degradation

**DOI:** 10.1186/s11658-026-00969-x

**Published:** 2026-06-24

**Authors:** Jie-Lin Wang, Qing-Hua Wu, Jing Yuan, Hai-Juan Bao, Xiang-Chun Huang, Jia-Chen Cheng, Yang Zhao, Shuo Chen

**Affiliations:** 1 Department of Obstetrics and Gynecology, Department of Gynecologic Oncology Research Office, Guangzhou Key Laboratory of Targeted Therapy for Gynecologic Oncology, Guangdong Provincial Key Laboratory of Major Obstetric Diseases, Guangdong Provincial Clinical Research Center for Obstetrics and Gynecology, Guangdong-Hong Kong-Macao Greater Bay Area Higher Education Joint Laboratory of Maternal-Fetal Medicine, The Third Affiliated Hospital, Guangzhou Medical University, No.63 Duobao Road, Liwan District, Guangzhou, Guangdong People’s Republic of China; 2 Cancer Hospital of Dalian University of Technology, Cancer Hospital of China Medical University, Liaoning Cancer Hospital & Institute, Shenyang, Liaoning China

**Keywords:** Ovarian cancer, piR-26681, Patient-derived organoids, m^6^A methylation, Homologous recombination repair

## Abstract

**Background:**

Survival rates for ovarian cancer drop sharply at late-stages due to late diagnosis. Although PARP inhibitors are effective in homologous recombination-deficient (HRD) tumors, their efficacy in homologous recombination (HR)-proficient ovarian cancer remains limited, highlighting the need for novel molecular targets to inhibit tumor progression and improve patient outcomes.

**Methods:**

Differentially expressed piRNAs were screened using ovarian cancer tissues from early- and advanced-stage patients. Functional studies were performed in ovarian cancer cell lines, xenograft models, and patient-derived organoids. Molecular mechanisms were investigated using RNA pulldown, RNA immunoprecipitation, MeRIP-seq, ubiquitination assays, and DNA damage analyses.

**Results:**

We identified piR-26681 as a piRNA significantly downregulated in advanced-stage ovarian cancer and associated with favorable prognosis. Functional assays demonstrated that piR-26681 suppressed ovarian cancer progression in cell lines, xenograft mouse models, and patient-derived organoids. Mechanistically, piR-26681 directly interacted with METTL3 and METTL14, enhancing their interaction, reducing their ubiquitination, and thereby increasing their protein stability. This stabilization promoted global m^6^A methylation in ovarian cancer cells. Increased m^6^A modification subsequently enhanced the stability of FBXO16 mRNA through the m^6^A reader IGF2BP2, leading to elevated FBXO16 expression. As an E3 ubiquitin ligase, FBXO16 further mediated the ubiquitination and degradation of MORF4L1, a key regulator of homologous recombination repair. Loss of MORF4L1 impaired HR repair, increased DNA damage accumulation, and sensitized ovarian cancer cells to the PARP inhibitor niraparib.

**Conclusions:**

Our study identifies a novel piR-26681–METTL3/METTL14–FBXO16–MORF4L1 regulatory axis that impairs DNA repair and suppresses ovarian cancer progression. piR-26681 represents a promising therapeutic target for sensitizing HR-proficient ovarian cancers to DNA-damaging therapies.

**Supplementary Information:**

The online version contains supplementary material available at https://doi.org/10.1186/s11658-026-00969-x.

## Introduction

Survival rates for epithelial ovarian cancer decline sharply by stage, with approximately 89% for stage I, 71% for stage II, 41% for stage III, and only 20% for stage IV [1]. Early ovarian cancer often presents with nonspecific symptoms, and over 75% of patients are diagnosed at a late-stage [2, 3], leading to high mortality and poor prognosis [4–6]. Therefore, this study aims to identify nucleic acid molecules downregulated in late-stage ovarian cancer tissues, evaluate their role in tumor progression, and provide novel targets for nucleic acid-based therapies.

PIWI-interacting RNAs (piRNAs) are a class of small noncoding RNAs that specifically associate with PIWI proteins and were initially characterized for their role in protecting the genome of germ cells [7, 8]. Recent research has demonstrated that piRNAs are also dysregulated in various cancers and play important roles in regulating tumor initiation and progression [9, 10]. In light of the sharp prognostic differences between early- and late-stage ovarian cancer and the challenge of early diagnosis, we aimed to identify piRNAs with potential value in both disease prediction and tumor suppression. By performing sequencing of ovarian cancer tissues from patients at both early and late stages, we identified piR-26681 as a piRNA significantly downregulated in advanced-stage disease.

Homologous recombination deficiency (HRD) ovarian cancers demonstrate therapeutic efficacy with PARP inhibitors [11]. However, approximately 50% of high-grade serous ovarian cancers lack BRCA mutations and still rely on functional homologous recombination (HR) pathways for DNA repair [12, 13], emphasizing the need to identify new therapeutic targets to modulate DNA repair in HR-proficient tumors. MORF4L1 (Mortality Factor 4 Like 1), a key component of the NuA4 histone acetyltransferase complex, plays a crucial role in chromatin remodeling and HR-mediated DNA repair [14]. Loss of MORF4L1 impairs the recruitment of HR factors such as PALB2 and RAD51, leading to genomic instability and increased sensitivity to DNA-damaging agents [15]. Emerging evidence indicates that m^6^A RNA modification plays an important role in the DNA damage response. The METTL3–m^6^A–YTHDC1 axis promotes the accumulation of DNA–RNA hybrids at double-strand break (DSB) sites and facilitates the recruitment of HR repair factors such as RAD51 and BRCA1 [16], suggesting that regulating the m^6^A pathway may represent a promising therapeutic strategy.

In this study, we identify piR-26681 as a regulator of MORF4L1. Overexpression of piR-26681 promotes the assembly of the METTL3/METTL14 methyltransferase complex, enhancing the m^6^A modification of FBXO16 mRNA, which increases FBXO16 expression. FBXO16, an E3 ubiquitin ligase, facilitates the ubiquitin-mediated degradation of MORF4L1, thus reducing its protein levels. This novel piR-26681–METTL3/METTL14–FBXO16–MORF4L1 axis provides new insights into noncoding RNA-mediated regulation of DNA repair in ovarian cancer cells. The MORF4L1 strategy can inhibit HR activity, causing otherwise HR-proficient tumors to acquire an HR-deficient phenotype, thereby enhancing the therapeutic effect of PARP inhibitors.

## Materials and methods

### Ovarian cancer specimens

A total of 85 ovarian cancer (OC) tissue specimens and 19 normal ovarian controls were obtained from the Third Affiliated Hospital of Guangzhou Medical University.The study protocol was approved by the Ethics Committee of the Third Affiliated Hospital of Guangzhou Medical University (Approval No. 2021–055). All participants provided written informed consent prior to enrollment, and all procedures were conducted in accordance with the Declaration of Helsinki (2013 revision).

### Cell culture and transfection

Human ovarian cancer cell lines A2780, CAOV3, HO8910, OVCAR3, SKOV3, and IOSE-80 were acquired from Jennio Biotech (Guangzhou, China) and ATCC (Manassas, VA, USA). All media were supplemented with 10% fetal bovine serum (FBS) and penicillin/streptomycin (100 U/mL). The cells were incubated at 37 °C in a 5% CO_2_ atmosphere. For transfection purposes, Lipofectamine 2000 was used according to the manufacturer’s instructions (Invitrogen, Carlsbad, CA, USA). The piR-26681 mimic, si-METTL14, si-METTL3, si-IGF2BP2, si-FBXO16, and the negative control were synthesized by Ribobio (Guangzhou, China). Additionally, the short hairpin (sh) RNA for piR-26681 was designed and synthesized by Han Heng Company (China).

### PDO models and AGO treatment

Ovarian cancer tissues were sectioned into 1–3-mm^3^ pieces and digested for 30 min in TrypLE (12,604,013, Thermo Fisher Scientific). Subsequently, a mixture of 40% complete medium and 60% Matrigel was used to support the organoid culture. Next, 20 µL droplets of this mixture were placed in preheated 48-well plates (Corning dishes). The organoids were passaged every 10–15 days. To the medium, 50 nmol/L AgopiR-26681 or/and niraparib (10 nm) was added, and the organoids were coincubated To visualize organoid proliferation, images were captured using an inverted microscope at 4 × magnification. All key organoid experiments were conducted with at least three independent biological replicate passages. PDO cultures were derived from at least three independent patients with ovarian cancer, and all key experiments were performed using at least three independent biological replicate passages.

### Data analysis

GraphPad Prism (version 9.5.0) was used for graphics generation and data analysis. The Kaplan–Meier curve was drawn by https://www.bioinformatics.com.cn, an online platform for data analysis and visualization. All experiments were performed at least three times. The data were expressed as the mean ± standard error of the mean. Statistical analysis was conducted using a two-sample *t*-test or two-way ANOVA, and a *p*-value of < 0.05 was considered to indicate a significant difference.

Additional detailed methods are provided in the Supporting Information.

## Result

### piR-26681 is decreased in ovarian cancer and correlates with early stage and favorable prognosis

**Fig. 1:**
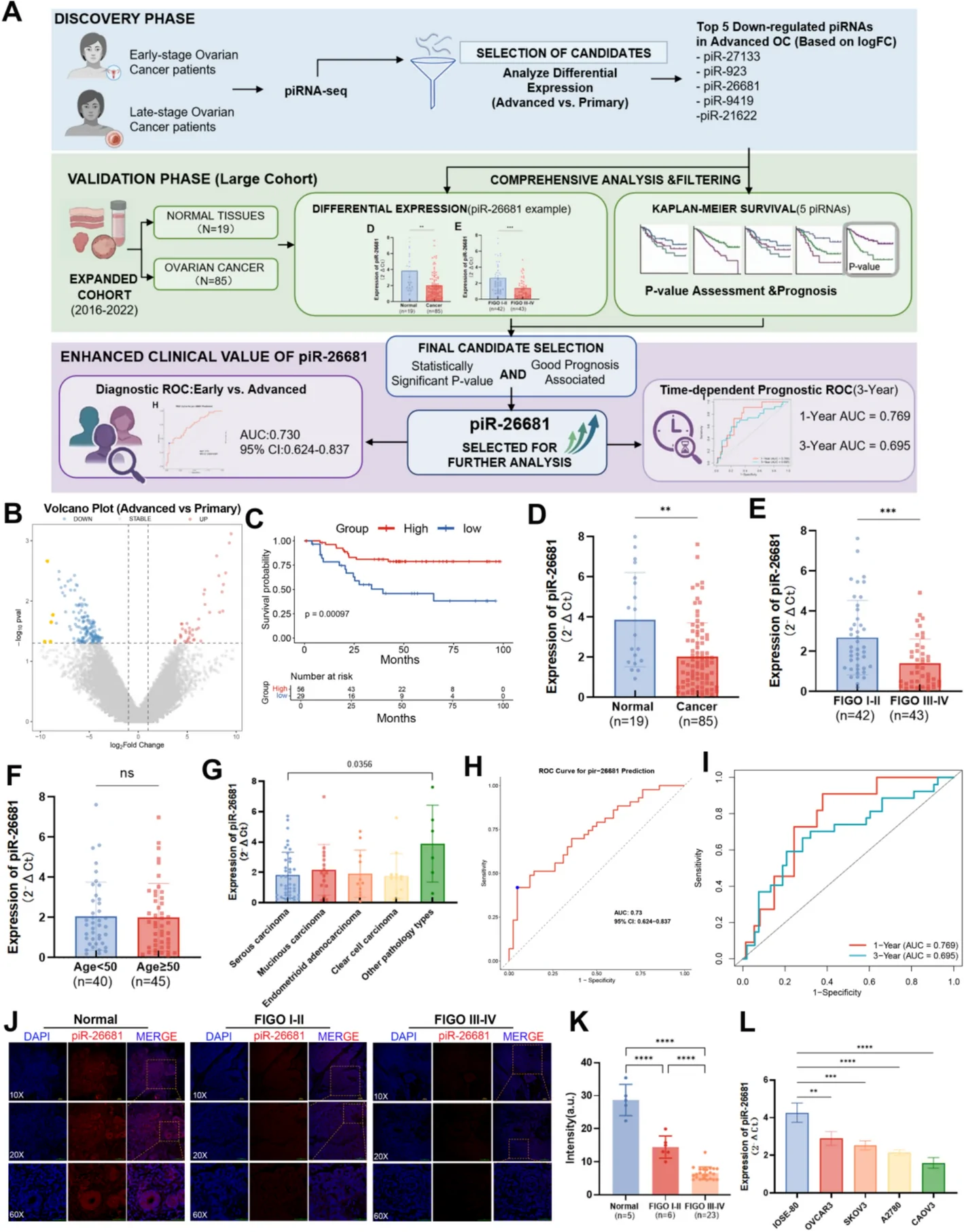
Identification and clinical relevance of piR-26681 in ovarian cancer. **A** Schematic overview of the multi-step screening strategy used to identify candidate piRNAs associated with ovarian cancer progression and prognosis. **B** Volcano plot showing differentially expressed piRNAs between advanced and early-stage ovarian cancer samples. **C** Kaplan–Meier analysis of progression-free survival (PFS) based on piR-26681 expression. **D** Comparison of piR-26681 expression between normal ovarian tissues (*n* = 19) and ovarian cancer tissues (*n* = 85). **E** piR-26681 expression in ovarian cancer tissues grouped by FIGO stage. **F** piR-26681 expression in ovarian cancer tissues by age group (< 50 versus > 50 years). **G** piR-26681 expression in ovarian cancer tissues grouped by pathology type. **H** Receiver operating characteristic (ROC) curve evaluating the diagnostic performance of piR-26681 in distinguishing early-stage from advanced ovarian cancer. **I** Time-dependent receiver operating characteristic (ROC) analysis showing the prognostic predictive value of piR-26681 expression for 1-year and 3-year survival. **J** Representative fluorescence in situ hybridization (FISH) images showing piR-26681 expression in normal ovarian tissue and ovarian cancer tissues at different stages. **K** Quantification of FISH signal intensity of piR-26681 in normal ovarian tissues and ovarian cancer tissues at different stages. **L** piR-26681 expression in ovarian cancer cell lines compared with normal ovarian epithelial cell line IOSE-80. Data are presented as mean ± SEM. *p < 0.05, **p < 0.01, ***p < 0.001

To identify piRNAs associated with ovarian cancer progression and prognosis, we performed a multi-step screening strategy (Fig. 1A). Briefly, piRNA sequencing was conducted on early-stage and late-stage ovarian cancer samples using Pandora sequencing to identify differentially expressed piRNAs (Fig. 1B). Based on the differential expression analysis, the top five downregulated piRNAs in advanced ovarian cancer were selected for further validation.

To validate these candidates, we analyzed a larger cohort consisting of 43 patients with early-stage ovarian cancer, 42 late-stage patients, and 19 normal ovarian tissues. Expression levels of the five candidate piRNAs were measured, and Kaplan–Meier survival analysis was subsequently performed. Among these candidates, only piR-26681 (piRBase ID: piR-hsa-26681) showed a significant association with improved progression-free survival (PFS), whereas the other four piRNAs did not exhibit a significant survival trend (Fig. 1C and Supplementary Fig. S1A–D). Therefore, piR-26681 was selected for further investigation.

Consistent with this observation, piR-26681 expression was significantly lower in ovarian cancer tissues compared with normal ovarian tissues (Fig. 1D). Moreover, its expression was further decreased in advanced-stage ovarian cancer compared with early-stage disease (Fig. 1E), suggesting a potential role in tumor progression. Clinicopathological analysis (Table 1) showed no significant association between piR-26681 expression and patient age (< 50 versus > 50 years; Fig. 1F). However, a significant difference in expression was observed among pathological subtypes, with serous carcinoma showing distinct expression levels compared with other histological subtypes (*p* = 0.0356), while no significant differences were detected among the remaining subtypes (*p* > 0.05; Fig. 1G).

**Table 1:** Correlation between PiR-26681 expression and clinical and pathological features in ovarian cancer

Clinicopathological features	*N*	PiR-26681 expression/U6	*p* value
Age			0.4401
< 50	40	2.0604 ± 1.6741	
> 50	45	2.0050 ± 1.6633	
The pathology types			
Serous carcinoma	40	1.8095 ± 1.5208	
Mucinous carcinoma	16	2.1673 ± 1.6766	
Endometrioid adenocarcinoma	11	2.1673 ± 1.6766	
Clear cell carcinoma	12	1.7640 ± 1.4711	
Other pathology types	6	3.8945 ± 2.5381	
FIGO stages			0.0002
I–II	42	2.6828 ± 1.8497	
III–IV	43	1.4129 ± 1.2149	

To further evaluate the clinical significance of piR-26681, receiver operating characteristic (ROC) analysis was performed in the cohort of patients with ovarian cancer. The ROC curve distinguishing early-stage and advanced-stage ovarian cancer showed an area under the characteristic curve (AUC) of 0.73 (95% CI: 0.624–0.837) (Fig. 1H), indicating a potential diagnostic value for distinguishing early and advanced ovarian cancer. In addition, time-dependent ROC analysis demonstrated that piR-26681 expression had predictive value for patient prognosis, with AUC values of 0.769 and 0.695 for 1-year and 3-year survival, respectively (Fig. 1I). To further assess the prognostic significance of piR-26681, Cox regression analyses were performed. Univariate analysis showed that piR-26681 expression was significantly associated with patient survival (Supplementary Table S3, HR = 0.64, *p* = 0.0046). However, after adjusting for age and FIGO stage in the multivariate model, piR-26681 was no longer an independent prognostic factor (*p* = 0.279; Supplementary Table S4), whereas FIGO stage remained strongly associated with survival. Consistently, stage-adjusted Kaplan–Meier analysis showed no significant difference between the high and low piR-26681 expression groups (*p* = 0.195; Supplementary Supplementary Fig. S1E).These findings suggest that piR-26681 expression is associated with tumor stage, which may partially explain its relationship with patient prognosis.To further strengthen the reliability of these findings, we analyzed an independent validation cohort. Consistent with the initial dataset, piR-26681 expression remained significantly lower in ovarian cancer tissues than in normal ovarian tissues (Supplementary Supplementary Fig. S1F) and was further reduced in advanced-stage tumors (Supplementary Supplementary Fig. S1G).

In addition, fluorescence in situ hybridization (FISH) analysis was performed using paraffin-embedded patient specimens. The results confirmed the same expression pattern, showing higher piR-26681 expression in normal and early-stage ovarian tissues but markedly decreased levels in advanced tumors (Fig. 1J–K). Additionally, piR-26681 expression was lower in ovarian cancer cell lines compared with the normal ovarian epithelial cell line IOSE-80, with the lowest expression observed in CAOV3 cells and the highest in OVCAR3 cells (Fig. 1L). Based on these findings, CAOV3 and OVCAR3 cell lines were selected for subsequent functional studies.

### piR-26681 inhibits malignant phenotypes of ovarian cancer cells

**Fig. 2:**
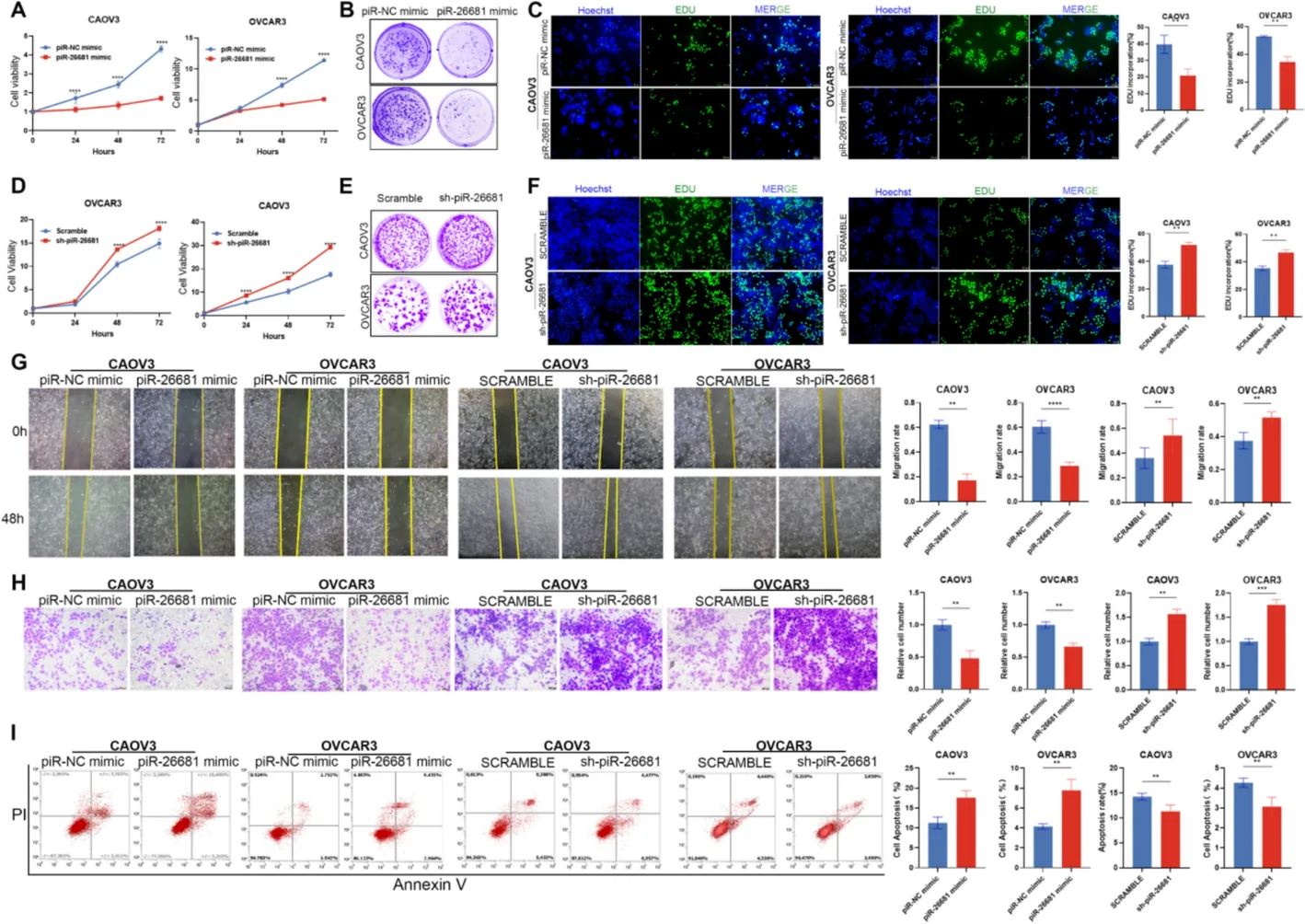
Effects of piR-26681 on ovarian cancer cell proliferation, migration, invasion, and apoptosis. **A** CCK-8 assay showing the effect of piR-26681 overexpression on cell proliferation in CAOV3 and OVCAR3 cells. **B** Plate colony formation assays evaluating the effect of piR-26681 overexpression on cell proliferation in CAOV3 and OVCAR3 cells. **C** EdU staining assessing cell proliferation in CAOV3 and OVCAR3 cells following piR-26681 overexpression**. D** CCK-8 assay evaluating cell proliferation in CAOV3 and OVCAR3 cells after piR-26681 downregulation. **E** Colony formation assays in CAOV3 and OVCAR3 cells following piR-26681 downregulation. **F** EdU staining assessing cell proliferation in CAOV3 and OVCAR3 cells after piR-26681 downregulation. **G** Wound healing assay assessing migration ability in CAOV3 and OVCAR3 cells after piR-26681 overexpression or knockdown. **H** Transwell invasion assay evaluating the invasive ability of CAOV3 and OVCAR3 cells after piR-26681 overexpression or knockdown. **I** Annexin V/PI staining to evaluate apoptosis in CAOV3 and OVCAR3 cells following piR-26681 overexpression or knockdown. Data are presented as mean ± SEM. *p < 0.05, **p < 0.01, ***p < 0.001. Bar graphs represent the quantification of the corresponding experimental results shown in the representative images

To assess the role of piR-26681 in ovarian cancer, we overexpressed it using a mimic and silenced it using sh-piR-26681 in CAOV3 and OVCAR3 cells, with transfection efficiency confirmed by qPCR (Supplementary Supplementary Fig. S2A–D). CCK-8 assays showed that piR-26681 overexpression inhibited cell proliferation, while silencing piR-26681 promoted growth (Fig. 2A, D). Plate colony formation assays (Fig. 2B, E) further confirmed these findings. EdU staining revealed reduced cell proliferation upon piR-26681 overexpression and increased proliferation with its knockdown (Fig. 2C, F). Wound healing (Fig. 2G) and transwell invasion assays (Fig. 2H) demonstrated that piR-26681 overexpression inhibited cell migration and invasion, while silencing piR-26681 had the opposite effect. Annexin V/PI staining (Fig. 2I) indicated that piR-26681 overexpression induced apoptosis, while silencing piR-26681 reduced apoptosis. In addition, functional assays of two nondifferentially expressed piRNAs identified from the sequencing dataset (piR-993 and piR-19620) showed no detectable effects on ovarian cancer cell proliferation, migration, invasion, or apoptosis (Supplementary Supplementary Fig. S1M–P), supporting the specificity of piR-26681. To further assess the role of piR-26681 in normal ovarian epithelial cells, similar functional assays were performed in IOSE-80 cells. Compared with ovarian cancer cells, piR-26681 overexpression showed only modest effects on proliferation, migration, and apoptosis, while its knockdown produced opposite but similarly limited effects (Supplementary Fig. S3A–E). These findings highlight the crucial role of piR-26681 in inhibiting the malignant behavior of ovarian cancer cells, suggesting that its modulation could impact the progression of the disease.

### piR-26681 regulates the stability of the METTL3–METTL14 complex

**Fig. 3:**
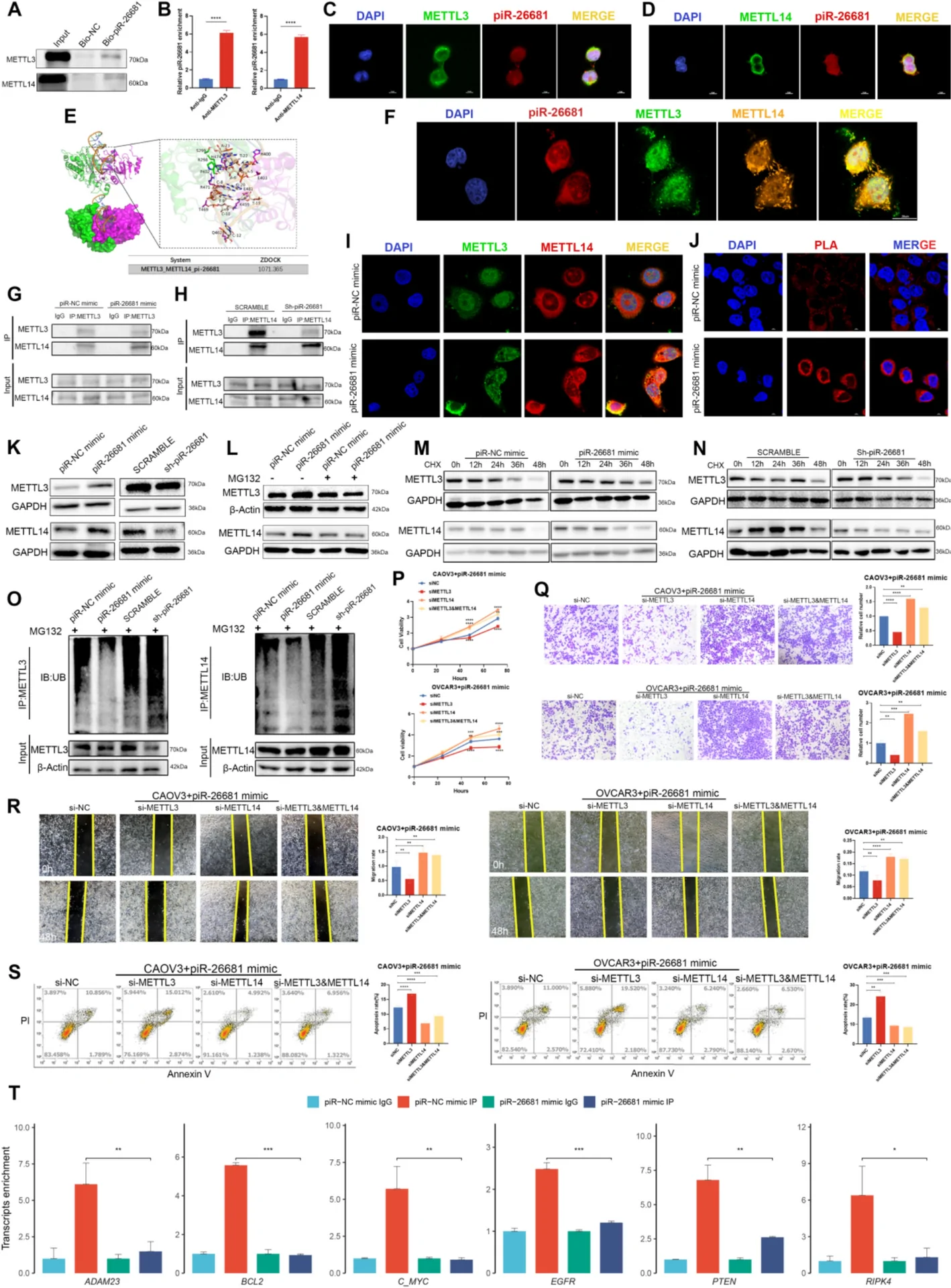
piR-26681 regulates the stability of the METTL3–METTL14 complex in ovarian cancer cells. **A** Pulldown assays identifying the interaction between piR-26681 and METTL3/METTL14. **B** RIP analysis of the association between piR-26681 and METTL3/METTL14. **C**, **D** Immunofluorescence images showing the cellular localization of piR-26681 with METTL3 (**C**) or METTL14 (**D**) in ovarian cancer cells. **E** Molecular docking prediction of piR-26681 binding sites on the METTL3–METTL14 heterodimer using ZDOCK software. **F** Immunofluorescence analysis of the colocalization of piR-26681, METTL3, and METTL14. **G**, **H** Co-IP assays examining the interaction between METTL3 and METTL14 after overexpression (**G**) or knockdown (**H**) of piR-26681. **I** Immunofluorescence images showing METTL3 and METTL14 staining in ovarian cancer cells following piR-26681 overexpression. **J** Proximity ligation assay (PLA) detecting METTL3–METTL14 interaction in ovarian cancer cells following piR-26681 overexpression. **K** Western blot analysis of METTL3 and METTL14 protein levels in cells with piR-26681 overexpression or knockdown. **L** Western blot analysis after treatment with the proteasome inhibitor MG132. **M**, **N** Cycloheximide (CHX) chase experiments assessing METTL3 and METTL14 protein stability in piR-26681 overexpressing (**M**) or knockdown (**N**) cells. **O** Ubiquitination assays showing the ubiquitination levels of METTL3 and METTL14 in cells with piR-26681 overexpression or knockdown. Data are presented as mean ± SEM. **P** CCK-8 assay assessing cell proliferation following individual or combined knockdown of METTL3 and METTL14 in piR-26681-overexpressing cells. **Q** Transwell invasion assay to assess cell invasive ability after individual or combined knockdown of METTL3 and METTL14in piR-26681-overexpressing cells. **R** Wound healing assay to evaluate cell migration after individual or combined knockdown of METTL3 and METTL14 in piR-26681-overexpressing cells. **S** Annexin V/PI staining to measure apoptosis in CAOV3 and OVCAR3 cells after METTL3 and/or METTL14 knockdown in piR-26681-overexpressing cells. **T** METTL3 RIP-qPCR showing enrichment of oncogenic transcripts (ADAM23, BCL2, c-MYC, EGFR, PTEN, and RIPK4) in METTL3 immunoprecipitates upon piR-26681 overexpression. Data are presented as mean ± SEM. *p < 0.05, **p < 0.01, ***p < 0.001

To explore the mechanisms through which piR-26681 exerts its anticancer effects in ovarian cancer, we predicted potential binding proteins using the RBPsuite website [17]. PiR-26681 was found to likely interact with METTL3 (score: 0.997) and METTL14 (score: 0.904). Since piRNAs regulate m^6^A-associated genes in cancer, investigating the interaction between piR-26681 and these methyltransferases is crucial. The interaction of piR-26681 with METTL3 and METTL14 was confirmed through pulldown assays (Fig. 3A) and RNA immunoprecipitation (RIP) (Fig. 3B). Furthermore, immunofluorescence experiments confirmed their colocalization (Fig. 3C, D).

Since piRNAs typically exert their function through PIWI proteins, we evaluated whether PIWIL1–4 could mediate the interaction between piR-26681 and the METTL3–METTL14 complex. Pulldown assays revealed that piR-26681 associates with PIWIL3, but not with PIWIL1, PIWIL2, or PIWIL4 (Supplementary Supplementary Fig. S4A). Co-immunoprecipitation assays further showed that PIWIL3 does not interact with METTL3 or METTL14 (Supplementary Supplementary Fig. S4B–C), indicating that PIWIL3 does not bridge the interaction between piR-26681 and the METTL3–METTL14 complex. These results suggest that piR-26681 regulates METTL3 and METTL14 through a PIWI-independent mechanism.

Since METTL3 and METTL14 typically function as heterodimers [18, 19], we used ZDOCK 3.0.2 software to predict the binding sites of piR-26681 on the METTL3–METTL14 heterodimer [20], with a high interaction score of 1071 (Fig. 3E). We identified the interface loop where METTL3 and METTL14 interact as the likely binding site [21]. Immunofluorescence analysis further confirmed the colocalization of piR-26681 with METTL3 and METTL14 (Fig. 3F). Co-IP assays demonstrated that overexpression of piR-26681 enhanced the interaction between METTL3 and METTL14 (Fig. 3G). Conversely, knockdown of piR-26681 reduced the interaction between these proteins (Fig. 3H).

Since piR-26681 binds to the interface loop where METTL3 and METTL14 interact, we hypothesis it may influence the association between these two proteins. To test this, we performed co-immunoprecipitation (Co-IP) assays. The results showed that overexpression of piR-26681 increased the amount of METTL14 pulled down by METTL3, indicating enhanced interaction between the two proteins (Fig. 3G). In contrast, knockdown of piR-26681 reduced the amount of METTL3 pulled down by METTL14 (Fig. 3H), suggesting a weakened interaction.To further support these findings, we conducted immunofluorescence colocalization experiments, which demonstrated that piR-26681 overexpression increased the colocalization of METTL3 and METTL14 (Fig. 3I). Additionally, we performed a Proximity Ligation Assay (PLA), which revealed a substantial increase in fluorescent PLA signals upon piR-26681 overexpression, providing further direct evidence that piR-26681 promotes the association between METTL3 and METTL14 (Fig. 3J).

Previous studies have demonstrated that the interaction between METTL3 and METTL14 stabilizes both proteins by preventing their ubiquitination and subsequent proteasomal degradation [22, 23]. Based on our findings that piR-26681 overexpression enhances the interaction between METTL3 and METTL14, we hypothesized that piR-26681 may promote the stability of these proteins by facilitating their association and inhibiting ubiquitination. Consistent with this hypothesis, overexpression of piR-26681 increased the protein levels of METTL3 and METTL14, whereas piR-26681 knockdown reduced their levels (Fig. 3k). Treatment with the proteasome inhibitor MG132 (Fig. 3L) and cycloheximide (CHX) chase experiments for METTL3 (Fig. 3M) and METTL14 (Fig. 3N) further confirmed that piR-26681 regulates their protein stability. Moreover, ubiquitination assays revealed that piR-26681 overexpression markedly decreased the ubiquitination of both METTL3 and METTL14, whereas knockdown of piR-26681 led to increased ubiquitination (Fig. 3O), supporting the notion that piR-26681 stabilizes the METTL3–METTL14 complex by preventing their ubiquitin-mediated degradation.

METTL3 has been shown to promote tumor progression in ovarian cancer [24], while METTL14 exhibits anticancer functions in various cancers, including ovarian cancer [25, 26]. Yang et al. reported that simultaneous knockdown of METTL3 and METTL14 enhances cell proliferation and invasiveness [27]. We hypothesize that piR-26681 suppresses tumor growth by strengthening the interaction between METTL3 and METTL14, thereby reshaping the transcript targeting of the METTL3–METTL14 complex. To investigate this, we assessed the effects of individual and combined knockdown of METTL3 and METTL14 on tumor progression in piR-26681-overexpressing cells. Transfection efficiency was confirmed by PCR (Supplementary Supplementary Fig. S5A, S5B). CCK-8 assays demonstrated that knockdown of METTL3 suppressed cell proliferation, whereas knockdown of METTL14, or simultaneous knockdown of both METTL3 and METTL14, promoted proliferation in piR-26681–overexpressing CAOV3 and OVCAR3 cells (Fig. 3P). Similar trends were observed in transwell invasion assayS (Fig. 3Q) and wound healing (Fig. 3R), where METTL3 knockdown reduced migration and invasion, while METTL14 knockdown or combined knockdown of both promoted them. Annexin V/PI staining (Fig. 3S) further confirmed that METTL3 knockdown increased apoptosis, while METTL14 knockdown or combined knockdown of both reduced apoptosis. To further explore the underlying mechanism, we examined whether piR-26681 influences the transcript recognition profile of the METTL3–METTL14 complex. METTL3 RIP assays revealed that the enrichment of several classical oncogenic transcripts (ADAM23, BCL2, c-MYC, EGFR, PTEN, and RIPK4) in METTL3 immunoprecipitates was markedly reduced under piR-26681 overexpression conditions (Fig. 3T).

Overall, our findings suggest that piR-26681 may contribute to ovarian cancer progression through modulation of the stability and interaction of the METTL3–METTL14 complex.

### piR-26681 shapes the m^6^A methylome and stabilizes downstream targets

**Fig. 4:**
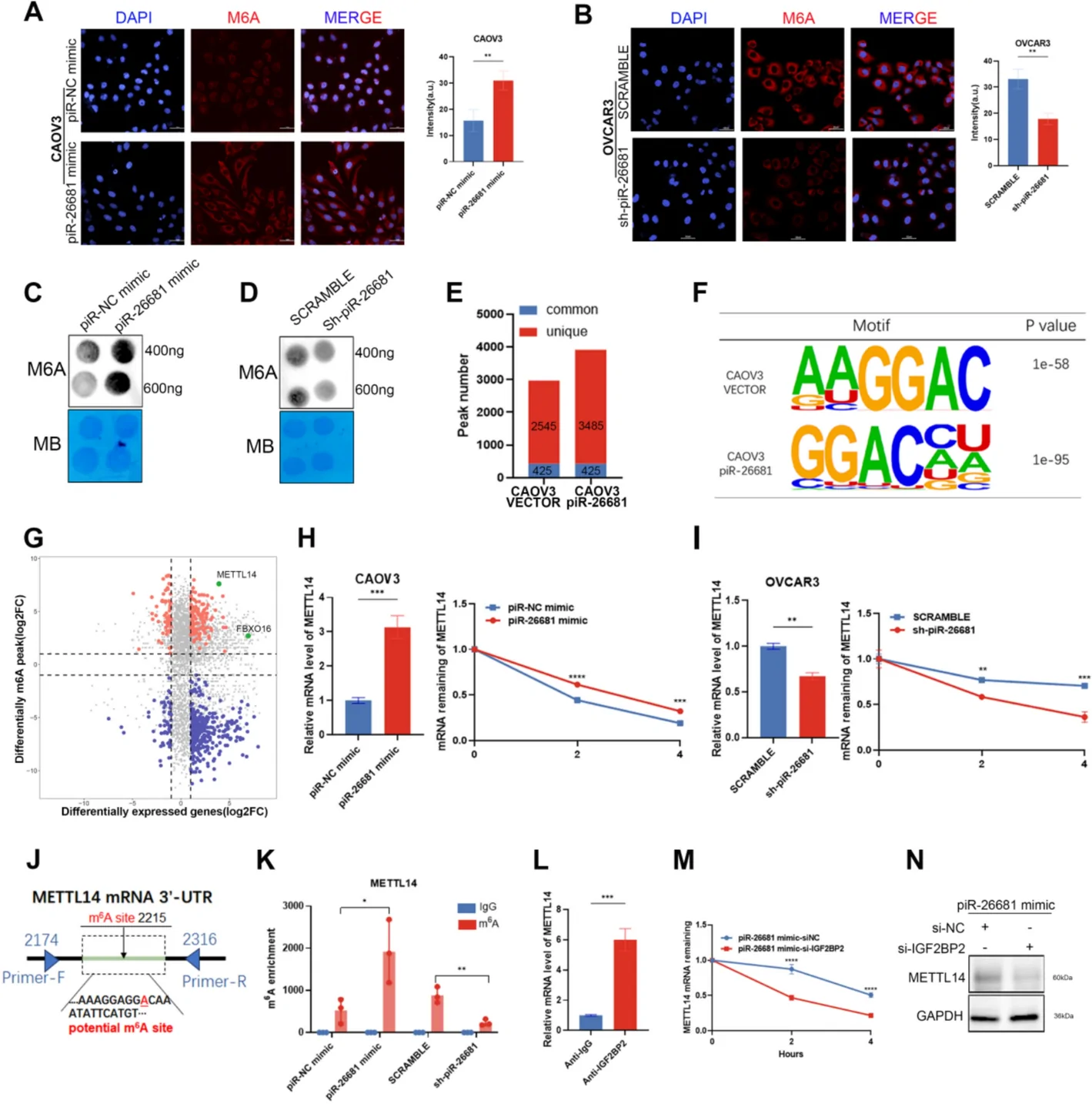
m^6^A methylome and downstream targets of piR-26681 in ovarian cancer. **A** Immunofluorescence analysis showing m^6^A levels in CAOV3 cells with piR-26681 overexpression, with quantification shown as a bar graph. **B** Immunofluorescence analysis showing m^6^A levels in OVCAR3 cells with piR-26681 knockdown, with quantification shown as a bar graph. **C** Dot blot assay to measure m^6^A levels in CAOV3 cells overexpressing piR-26681. **D** Dot blot assay to measure m^6^A levels in OVCAR3 cells with piR-26681 downregulation. **E** MeRIP-seq analysis of mRNA m^6^A peaks in CAOV3 cells after piR-26681 overexpression. **F** Motif enrichment analysis of MeRIP-seq data showing the GGAC consensus motif. **G** Differentially expressed genes with m^6^A modification. **H** qPCR analysis of METTL14 mRNA levels and RNA stability in CAOV3 cells with piR-26681 overexpression. **I** qPCR analysis of METTL14 mRNA levels and RNA stability in OVCAR3 cells with piR-26681 knockdown. **J** Schematic diagram of the predicted m^6^A modification site within the METTL14 3′UTR and the primer design used for MeRIP-qPCR validation. **K** MeRIP-qPCR analysis showing m^6^A enrichment on METTL14 mRNA following piR-26681 overexpression or knockdown. **L** RIP assay showing the binding of IGF2BP2 to METTL14 mRNA. **M** mRNA stability assay in CAOV3 cells overexpressing piR-26681 following IGF2BP2 knockdown, showing the regulation of METTL14 mRNA stability. **N** Western blot analysis of METTL14 protein levels in CAOV3 cells after IGF2BP2 knockdown. Data are presented as mean ± SEM. *p < 0.05, **p < 0.01, ***p < 0.001

We found that piR-26681 overexpression increased global m^6^A levels in CAOV3 cells, as shown by both immunofluorescence and dot blot assays (Fig. 4A, C), while knockdown of piR-26681 decreased m^6^A levels in OVCAR3 cells (Fig. 4B, D). MeRIP-seq analysis confirmed an increase in mRNA m^6^A peaks with piR-26681 overexpression (Fig. 4E), with enrichment of the GGAC consensus motif (Fig. 4F). As shown in the integrated scatter plot (Fig. 4G), genes exhibiting coordinated changes in both m^6^A modification and mRNA expression were identified. The enrichment results for m^6^A-Seq peaks are shown in Supplementary Supplementary Fig. S6A-D, whereas the enrichment results for RNA-Seq differential genes are presented in Supplementary Supplementary Fig. S6E-G. Notably, many of the enriched pathways are associated with protein ubiquitination and cancer progression, including ubiquitin-protein transferase activity, cadherin binding, focal adhesion, and cell cycle regulation. Notably, METTL14 ranked among the top 10 genes with increased m^6^A modification. Among transcripts with elevated m^6^A enrichment, FBXO16 showed the largest log2FC change in the RNA-seq dataset, prompting us to select FBXO16 for further mechanistic investigation (Fig. 4G).

Overexpression of piR-26681 increased METTL14 mRNA levels and enhanced RNA stability (Fig. 4H), while knockdown of piR-26681 resulted in the opposite effect (Fig. 4I). Guided by the MeRIP-seq peak prediction, primers were designed targeting the putative m^6^A modification site within the METTL14 3′UTR (2215 site), and MeRIP–qPCR was performed to validate m^6^A enrichment. As shown in Fig. 4J–K, m^6^A enrichment at this site was significantly increased upon piR-26681 overexpression, whereas knockdown of piR-26681 markedly reduced m^6^A enrichment, confirming that piR-26681 promotes m^6^A modification of METTL14 transcripts. IGF2BP2, a known m^6^A reader protein, binds to and stabilizes m^6^A-modified RNAs [28]. Prediction tools from the RBP binding prediction site indicated that IGBF2BP2 would bind to the m^6^A modification sites identified on METTL14 [17, 29]. Subsequent RIP experiments confirmed that IGF2BP2 binds to METTL14 mRNA (Fig. 4L). Knockdown of IGF2BP2 in CAOV3 cells overexpressing piR-26681 reduced METTL14 mRNA stability (Fig. 4M), as well as decreased protein level (Fig. 4N). These results suggest that piR-26681 may enhance METTL14 expression through increased m^6^A modification and improved transcript stability.

### piR-26681-mediated FBXO16 regulation promotes MORF4L1 ubiquitination and DNA damage in ovarian cancer cells

**Fig. 5:**
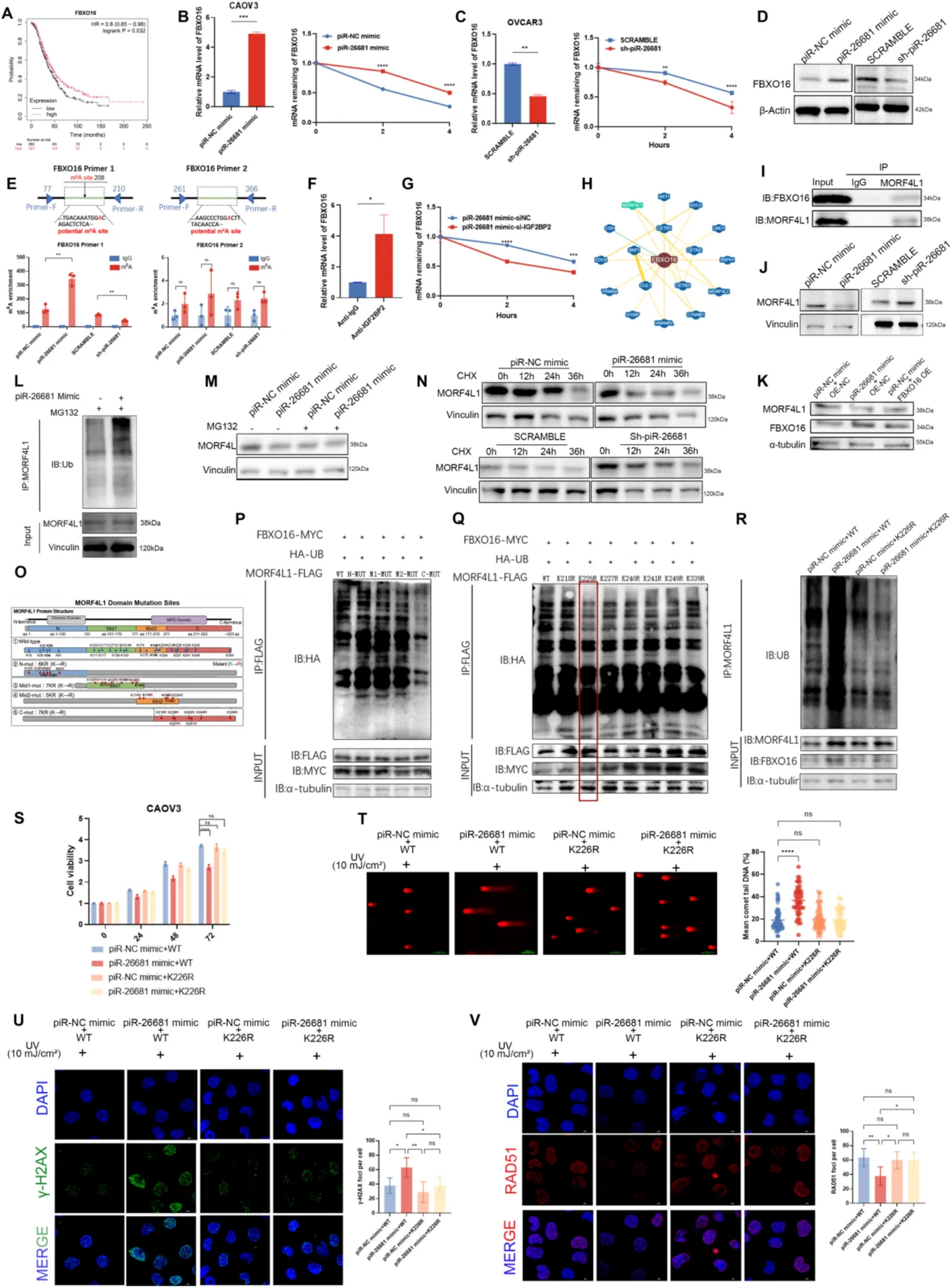
piR-26681-mediated FBXO16 regulation promotes MORF4L1 ubiquitination and DNA damage in ovarian cancer cells. **A** Kaplan–Meier analysis of FBXO16 levels and overall survival in patients with ovarian cancer. **B** qPCR analysis of FBXO16 mRNA levels and RNA stability in CAOV3 cells with piR-26681 overexpression. **C** qPCR analysis of FBXO16 mRNA levels and RNA stability in OVCAR3 cells with piR-26681 knockdown. **D** Western blot analysis of FBXO16 protein levels in CAOV3 cells with piR-26681 overexpression and downregulation. **E** Schematic of FBXO16 m^6^A-enriched regions and MeRIP–qPCR validation using two primer sets. **F** RIP assay showing the binding of IGF2BP2 to FBXO16 mRNA. **G** qPCR analysis of FBXO16 mRNA stability after IGF2BP2 knockdown in CAOV3 cells overexpressing piR-26681. **H** Interaction network of FBXO16 interactors identified using the BioGRID database. **I** Co-IP assays confirming the interaction between FBXO16 and MORF4L1. **J** Western blot analysis showing MORF4L1 protein levels in CAOV3 cells with piR-26681 overexpression and downregulation. **K** Western blot analysis of MORF4L1 protein levels following FBXO16 or piR-26681 overexpression. **L** Western blot showing MORF4L1 ubiquitination levels in piR-26681 overexpressing cells. **M** Western blot analysis of MORF4L1 in CAOV3 cells treated with MG132. **N** CHX chase assays showing MORF4L1 stability in piR-26681 overexpressing or knockdown cells. **O** Schematic illustration of predicted lysine residues in MORF4L1 and the corresponding stepwise truncation mutants. **P** Ubiquitination assays of MORF4L1 truncation mutants. **Q** Ubiquitination assays of C-terminal lysine-to-arginine (K → R) mutants. **R** Ubiquitination assay of wild-type (WT) and K226R mutant MORF4L1 under piR-26681 overexpression. **S** Cell proliferation analysis (CCK-8) of WT and K226R mutant conditions. **T** Comet assay following UV irradiation in WT and K226R cells with or without piR-26681 overexpression. **U** IF analysis of γ-H2AX foci in WT and K226R cells following UV treatment with or without piR-26681 overexpression. **V** IF analysis of RAD51 foci in WT and K226R cells following UV treatment with or without piR-26681 overexpression. Data are presented as mean ± SEM. *p < 0.05, **p < 0.01, ***p < 0.001

Previous studies have shown that FBXO16 inhibits ovarian cancer development [26, 30]. Kaplan–Meier analysis showed that higher FBXO16 levels correlate with better overall survival in patients with ovarian cancer (Fig. 5A) (https://doi.org/10.1007/s11357-023-00742-4). Overexpression of piR-26681 increased FBXO16 mRNA levels and stability (Fig. 5B), while knockdown of piR-26681 reduced both (Fig. 5C). In agreement with these results, piR-26681 overexpression elevated FBXO16 protein levels, while its depletion reduced FBXO16 expression (Fig. 5D). Based on the MeRIP-seq peak prediction, two candidate m^6^A-enriched regions within the FBXO16 CDS were validated by MeRIP–qPCR. Primer 1 (site 208) showed significantly increased m^6^A enrichment upon piR-26681 overexpression and decreased enrichment after piR-26681 knockdown. In contrast, Primer 2 (site 320) did not exhibit significant changes (Fig. 5E), indicating that piR-26681 specifically enhances m^6^A modification at the 208 region of FBXO16. Bioinformatics prediction suggested that this m^6^A region could be recognized by the m^6^A reader IGF2BP2. RIP experiments confirmed that IGF2BP2 binds to FBXO16 mRNA (Fig. 5F). Knockdown of IGF2BP2 in piR-26681 overexpressing cells reduced FBXO16 mRNA stability (Fig. 5G) and protein levels (Supplementary Supplementary Fig. S7A). Additionally, knockdown of METTL3 and METTL14 in piR-26681 overexpressing cells restored FBXO16 upregulation (Supplementary Fig. S7B). Together, these results indicate that piR-26681 enhances METTL3–METTL14–mediated m^6^A modification on FBXO16 transcripts and promotes IGF2BP2-dependent stabilization of FBXO16 mRNA.

To examine the biological function of FBXO16 in ovarian cancer, we explored its interacting proteins using the BioGRID database [31]. A total of 17 proteins were identified as FBXO16 interactors. Notably, Mortality factor 4 like 1 (MORF4L1, also known as MRG15), a member of the MRG transcription factor family [15], was among them (Fig. 5H). MORF4L1 is important for DNA double-strand break repair by facilitating the recruitment of the PALB2–BRCA2 complex to DNA damage sites and maintaining chromatin accessibility [15, 32–34]. Interestingly, database analysis showed that MORF4L1 interacts only with FBXO16 and FBXO32 among F-box proteins (Supplementary Fig. S8A). However, endogenous COIP experiments revealed that MORF4L1 interacted with FBXO16 but not FBXO32 (Fig. 5I, Supplementary Fig. S8B). We also found that overexpression of piR-26681 reduced MORF4L1 protein levels, while knockdown had the opposite effect (Fig. 5J). Similarly, FBXO16 overexpression also decreased MORF4L1 protein levels (Fig. 5K), suggesting that MORF4L1 may be a downstream target of FBXO16. Supporting this regulatory relationship, FBXO16 overexpression phenocopied the tumor-suppressive effects of piR-26681, including inhibition of proliferation, migration, and invasion, induction of apoptosis, and increased DNA damage responses, as demonstrated by CCK8, EdU, wound healing, Transwell, comet, and γ-H2AX assays (Supplementary Fig. S9A–H). Conversely, restoration of FBXO16 expression significantly rescued the protumorigenic phenotypes induced by piR-26681 knockdown (Supplementary Fig. S9I–N).

Mechanistically, piR-26681 overexpression also increased MORF4L1 ubiquitination (Fig. 5L). Treatment with MG132 (Fig. 5M) and CHX chase assays in overexpression and knockdown conditions further supported this finding (Fig. 5N). Notably, the downregulation of MORF4L1 by piR-26681 was reversed when FBXO16 (Supplementary Fig. S7C), IGF2BP2 (Supplementary Fig. S7D), or METTL3/METTL14 (Supplementary Fig. S7E) were depleted. These results demonstrate that piR-26681 upregulates FBXO16 via the METTL3–METTL14 complex, leading to increased MORF4L1 ubiquitination and degradation. To identify the ubiquitination site of MORF4L1, potential lysine residues were predicted using PhosphoSitePlus and subsequently examined by stepwise truncation mutagenesis (Fig. 5O). Mutation of the C-terminal region markedly reduced MORF4L1 ubiquitination (Fig. 5P). Further mutagenesis identified K226 as the critical ubiquitination site (Fig. 5Q). Importantly, overexpression of piR-26681 failed to increase MORF4L1 ubiquitination in the K226R mutant, confirming that K226 is the major ubiquitination site regulated by piR-26681 (Fig. 5R). Functionally, mutation of this ubiquitination site completely abolished the tumor-suppressive effects of piR-26681, including restored proliferation, migration, invasion, and reduced apoptosis (Fig. 5S and Supplementary Fig. S9O–R). Upon UV-induced DNA damage, piR-26681 overexpression increased DNA breaks as shown by comet assays (Fig. 5T), elevated γ-H2AX foci (Fig. 5U), and reduced RAD51 foci formation (Fig. 5V). Collectively, these results demonstrate that piR-26681 promotes FBXO16 expression through METTL3–METTL14–dependent m^6^A modification and IGF2BP2-mediated stabilization, thereby enhancing FBXO16-mediated ubiquitination and degradation of MORF4L1 and ultimately promotes DNA damage accumulation and inhibits homologous recombination repair.

**Fig. 6:**
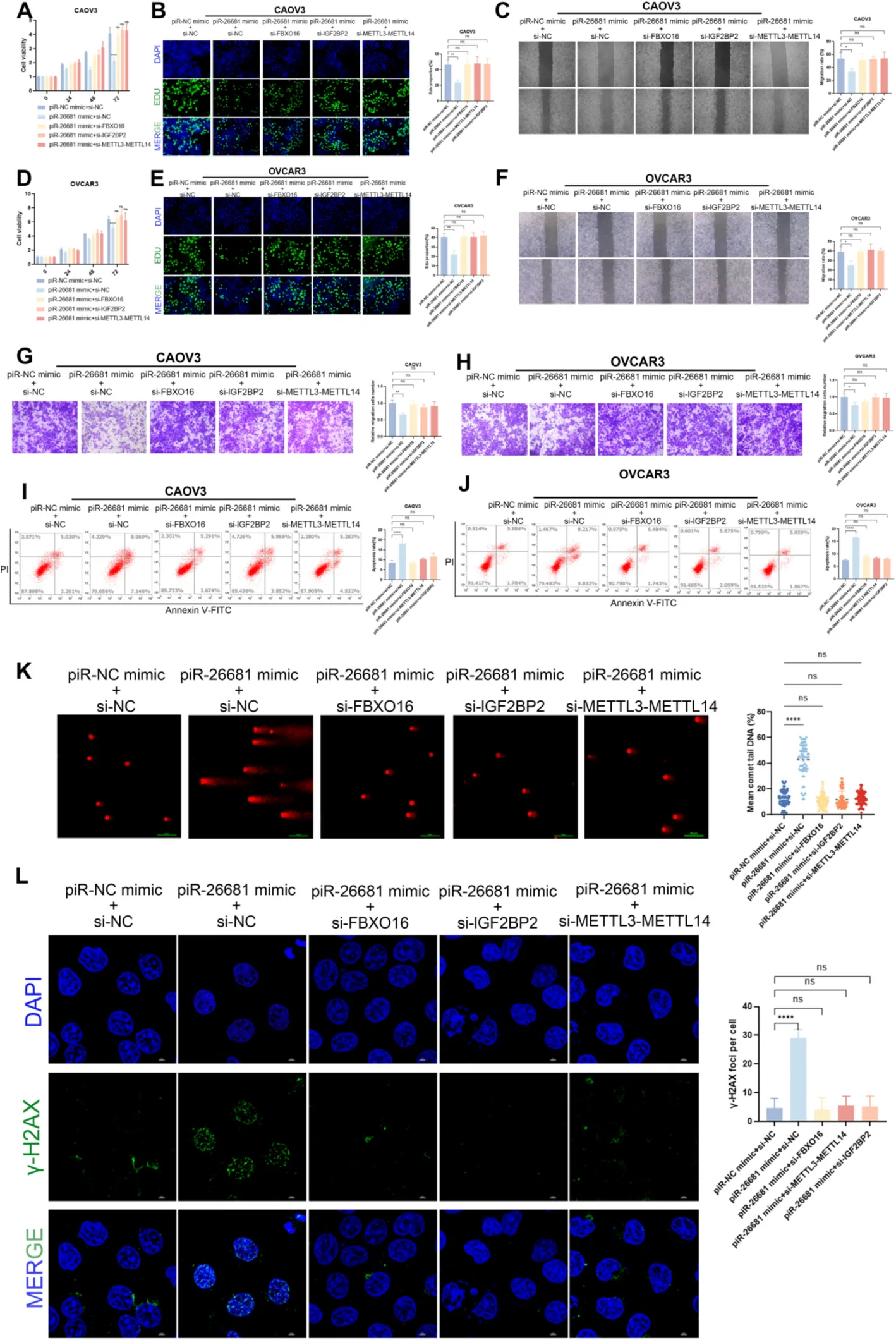
Rescue of piR-26681-mediated effects by knockdown of FBXO16, IGF2BP2, and METTL3/METTL14 in CAOV3 and OVCAR3 cells. **A**, **D** Cell proliferation measured by CCK-8 assays in CAOV3 and OVCAR3 cells, including control and cells overexpressing piR-26681 with or without knockdown of FBXO16, IGF2BP2, or METTL3/METTL14. **B**, **E** DNA synthesis assessed by EdU incorporation under the same knockdown conditions. **C**, **F** Cell migration analyzed by wound-healing assays under the same conditions. **G**, **H** Cell invasion assessed by Transwell assays under the same conditions. **I**, **J** Apoptosis measured by Annexin V-FITC/PI flow cytometry under the same conditions. **K** Comet assays performed to evaluate DNA damage under the same conditions. **L** Immunofluorescence staining of γ-H2AX foci under the same conditions. Data are presented as mean ± SEM. *p < 0.05, **p < 0.01, ***p < 0.001

To further validate the functional role of the piR-26681–FBXO16 axis, we performed rescue experiments by knocking down FBXO16, IGF2BP2, or METTL3/METTL14 in CAOV3 and OVCAR3 cells overexpressing piR-26681. Functional assays showed that depletion of these proteins markedly attenuated the tumor-suppressive effects induced by piR-26681 overexpression. Specifically, knockdown of FBXO16, IGF2BP2, or METTL3/METTL14 significantly restored cell proliferation as measured by CCK-8 assays (Fig. 6A, D) and increased DNA synthesis in EdU assays (Fig. 6B, E). Similarly, the inhibitory effects of piR-26681 on cell migration and invasion were partially reversed following depletion of these proteins, as demonstrated by wound-healing assays (Fig. 6C, F) and Transwell invasion assays (Fig. 6G, H). Consistently, flow cytometry analysis revealed that the increase in apoptosis induced by piR-26681 overexpression was significantly reduced after knockdown of FBXO16, IGF2BP2, or METTL3/METTL14 (Fig. 6I, J). Moreover, comet assays showed that the enhanced DNA damage caused by piR-26681 overexpression was markedly alleviated upon depletion of these proteins (Fig. 6K). In agreement with this observation, γ-H2AX IF staining demonstrated that the accumulation of DNA damage foci induced by piR-26681 was significantly reduced after knockdown of FBXO16, IGF2BP2, or METTL3/METTL14 (Fig. 6L). Together, these findings support a model in which piR-26681 promotes FBXO16 expression through METTL3–METTL14–dependent m^6^A modification and IGF2BP2-mediated stabilization, thereby enhancing FBXO16-mediated ubiquitination and degradation of MORF4L1 and ultimately promotes DNA damage accumulation and inhibits homologous recombination repair.

### piR-26681 enhances niraparib sensitivity in ovarian cancer

**Fig. 7:**
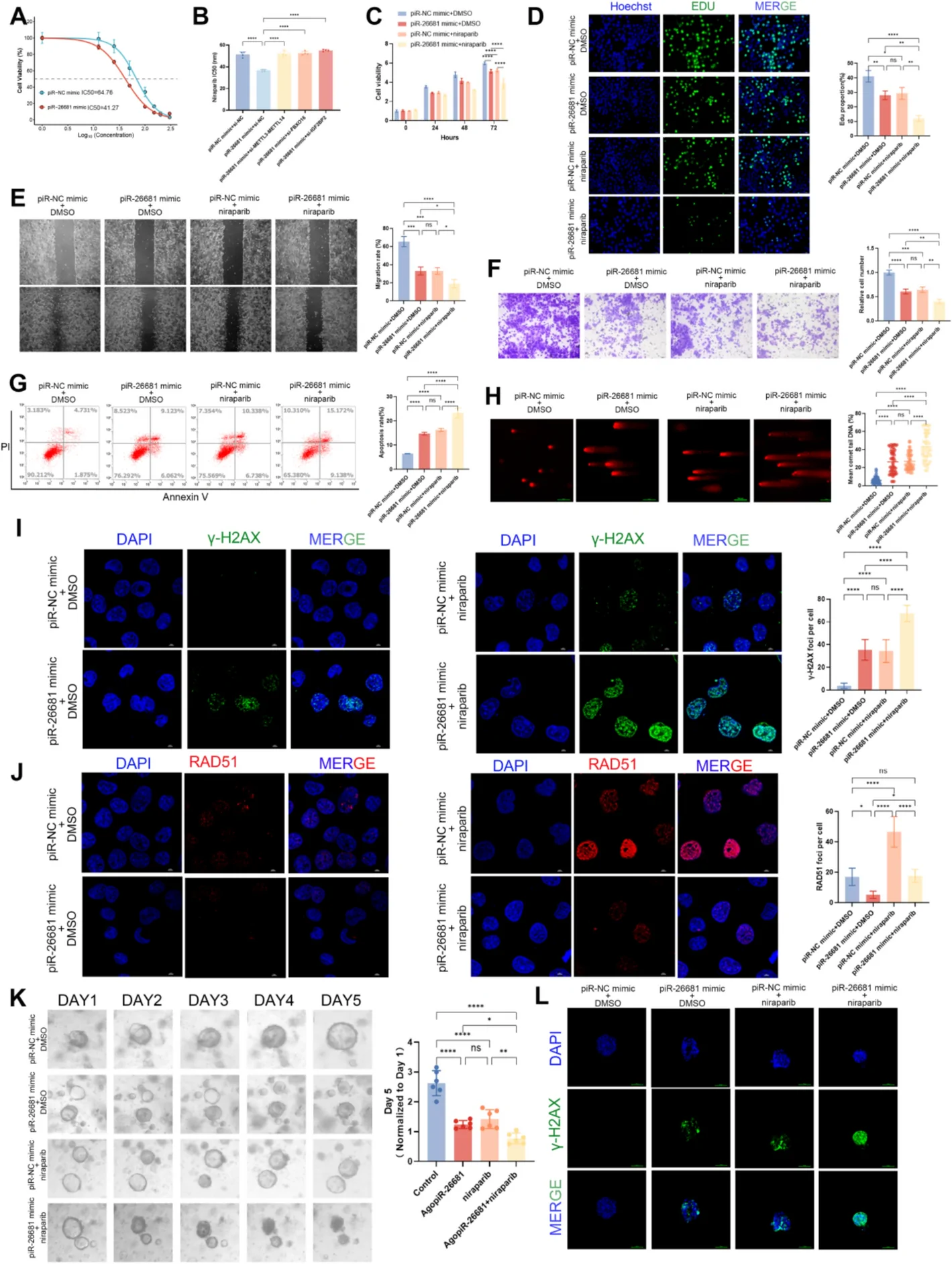
piR-26681 enhances niraparib sensitivity and impairs homologous recombination repair in ovarian cancer cells. **A** Dose–response curves showing the effect of piR-26681 overexpression on niraparib sensitivity in CAOV3 cells. **B** IC_50_ analysis showing that knockdown of downstream target genes of piR-26681 restores niraparib sensitivity. **C** CCK-8 assay evaluating cell viability under different treatment conditions. **D** EdU incorporation assay assessing cell proliferation after piR-26681 overexpression and niraparib treatment. **E** Wound healing assay showing the migration ability of ovarian cancer cells under different treatment conditions. **F** Transwell invasion assay evaluating the invasive ability of ovarian cancer cells. **G** Annexin V/PI staining analyzed by flow cytometry to evaluate apoptosis. **H** Comet assay assessing DNA damage levels in ovarian cancer cells under different treatments. **I** IF staining of γ-H2AX under different treatments. **J** IF staining of RAD51 under different treatments. **K** Bright-field images showing the growth of patient-derived organoids (PDOs) under different treatment conditions. **L** IF staining of γ-H2AX in PDOs. Data are presented as mean ± SEM. *p < 0.05, **p < 0.01, ***p < 0.001

To explore the potential translational relevance of piR-26681 in the context of PARP inhibition, we first evaluated the effect of piR-26681 overexpression on ovarian cancer cell sensitivity to niraparib. Overexpression of piR-26681 significantly decreased the IC50 of niraparib in CAOV3 (Fig. 7A). Importantly, simultaneous knockdown of piR-26681 downstream target genes restored the IC50 (Fig. 7B).

We next evaluated the combined effects of piR-26681 overexpression and niraparib treatment on ovarian cancer cells. CCK-8 assays revealed a significant reduction in cell viability in the combination group compared with either treatment alone (Fig. 7C). Consistently, EdU incorporation assays showed markedly decreased DNA synthesis following the combined treatment (Fig. 7D). In addition, cell migration and invasion were further suppressed by piR-26681 overexpression in the presence of niraparib, as demonstrated by wound healing (Fig. 7E) and Transwell invasion assays (Fig. 7F). Moreover, Annexin V/PI staining indicated a substantial increase in apoptotic cells in the piR-26681 plus niraparib group compared with the control groups (Fig. 7G).

DNA damage was further confirmed by comet assays, showing a significant increase in tail moment in the combination treatment (Fig. 7H). Immunofluorescence analysis of γ-H2AX foci demonstrated elevated DNA double-strand breaks (Fig. 7I). Moreover, RAD51 immunofluorescence revealed robust RAD51 foci formation after niraparib treatment, whereas piR26681 overexpression markedly suppressed RAD51 recruitment and significantly reduced niraparib-induced RAD51 foci, indicating that piR26681 impairs homologous recombination DNA repair (Fig. 7J).

To investigate the effect of piR26681 and niraparib in a more clinically relevant model, we generated patient-derived organoids (PDOs). Consistent with the results observed in cell lines, the combination treatment exhibited the most pronounced antitumor effect in PDOs (Fig. 7K). Immunofluorescence staining for γH2AX further confirmed increased DNA damage in organoids receiving the combination treatment (Fig. 7L).

Together, these results demonstrate that piR-26681 overexpression sensitizes ovarian cancer cells to niraparib by impairing homologous recombination repair, leading to increased DNA damage, reduced proliferation, and enhanced apoptosis. These findings provide functional evidence supporting the potential translational relevance of piR-26681 in combination with PARP inhibitor therapy.

### piR-26681 suppresses ovarian cancer progression in vivo and in organoids

**Fig. 8:**
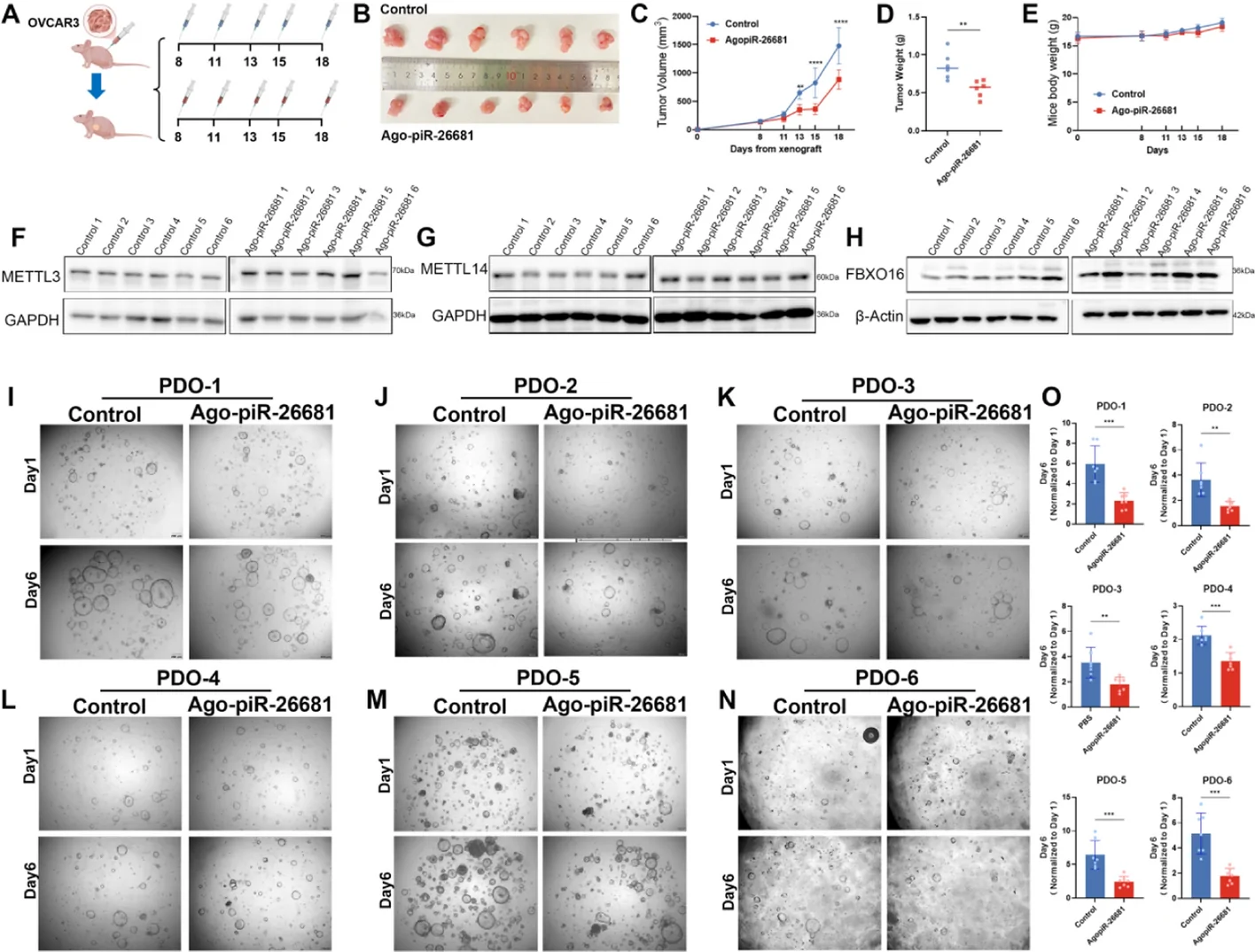
Effect of piR-26681 on ovarian cancer progression in xenograft and organoid models. **A** Schematic of the experimental setup for the xenograft model using OVCAR3 cells. **B** Tumor images from the control and Ago-piR-26681 groups. **C** Tumor growth over time in control and Ago-piR-26681-treated mice. **D** Tumor weight comparison between the control and Ago-piR-26681-treated groups. **E** Body weight measurements of mice in both groups. **F–H** Western blot analysis showing METTL3 (**F**), METTL14 (**G**), and FBXO16 (**H**) protein levels in tumor tissues from the xenograft model. **I**–**N** Representative images of 6 OC-PDOs treated with Ago-piR-26681, showing changes in organoid size. **O** Quantification of organoid size from various OC-PDO lines after Ago-piR-26681 treatment. Data are presented as mean ± SEM. *p < 0.05, **p < 0.01, ***p < 0.001

Previous experiments demonstrated the strong antitumor effect of piR-26681. To further validate these findings, we upregulated piR-26681 using agopiR-26681 in both xenograft models and ovarian cancer patient-derived organoids (OC-PDOs). In the xenograft model established with OVCAR3 cells (Fig. 8A), agopiR-26681 treatment significantly reduced tumor volume (Fig. 8B, C) and weight compared with the control group (Fig. 8D), without affecting mouse body weight (Fig. 8E). Western blot analysis of tumor tissues showed increased levels of METTL3, METTL14, and FBXO16 proteins in the agopiR-26681 group (Fig. 8F–H), which was further confirmed by immunohistochemistry (Supplementary Fig. S10A–C). Consistent with the in vivo results, agopiR-26681 treatment also led to a significant reduction in organoid size in multiple OC-PDO lines (Fig. 8I–N), as quantified in Fig. 8O. These results further support piR-26681 as a promising therapeutic target for ovarian cancer.

## Discussion

Ovarian cancer accounts for 2.5% of all malignancies among females but represents 5% of female cancer deaths due to its low survival rates, largely driven by late-stage diagnoses [35]. However, over 75% of ovarian cancer cases are diagnosed at late-stages, where therapeutic options become limited, and patient outcomes are poor [2, 3]. Thus, identifying molecular targets that can slow or prevent the progression of the disease, especially in late-stage, is crucial. In this study, we identified piR-26681, a piRNA significantly downregulated in late-stage ovarian cancer, as a potential therapeutic target. Our findings suggest that piR-26681 plays a critical role in inhibiting ovarian cancer progression by regulating m^6^A modification and DNA damage repair pathways.

M^6^A is the predominant chemical modification of mRNA, influencing gene expression and various bioprocesses [36]. In recent years, the dysregulation of the m^6^A modification machinery, comprising writers, erasers, and readers, has emerged as a key factor in tumorigenesis and cancer progression [37]. piRNAs, small noncoding RNAs, have been shown to interact with m^6^A-associated genes, either promoting or inhibiting cancer. For instance, piRNA-14633 binds to the 3’ UTR of METTL14, enhancing m^6^A methylation and contributing to cervical cancer development [38]. Although piRNAs are traditionally associated with PIWI proteins in germline cells, accumulating evidence suggests that certain piRNAs can exert regulatory functions independently of PIWI proteins in somatic cancer cells, often through interactions with RNA-binding proteins.

Our study identified an interaction between piR-26681 and the METTL3 and METTL14 proteins, key components of the m^6^A methyltransferase complex. Surprisingly, while piR-26681 is known for its anticancer properties, it appears to increase both METTL3 (oncogene) [39, 40] and METTL14 (tumor suppressor) [25, 41, 42] levels in ovarian cancer. Previous studies showed that blocking both METTL3 and METTL14 promotes cell growth in endometriosis, highlighting their complex roles [27]. We silenced METTL3 and METTL14 individually and together in piR-26681–overexpressing CAOV3 and OVCAR3 cells. Our results showed that METTL3 depletion inhibited cell growth, while silencing METTL14 or both proteins promoted growth. This apparent paradox suggests that the biological consequences of m^6^A regulation cannot be inferred solely from the expression level of individual methyltransferases. Instead, the functional outcome may depend on the composition and regulatory state of the METTL3–METTL14 complex as well as the specific transcripts targeted for modification. Accumulating evidence indicates that the oncogenic activity of METTL3 is highly context-dependent and largely determined by the specific transcripts it recognizes and modifies. Moreover, post-translational modifications of METTL3 itself can influence its transcript recognition and regulatory functions [43]. Therefore, measuring METTL3 protein abundance alone does not fully reflect its functional outcome, Mechanistically, molecular docking analysis indicated that piR-26681 may bind to key regions of the METTL3–METTL14 complex involved in RNA recognition, including the positively charged region of METTL3 (amino acids 465–478) and the RNA-binding interface of METTL14 (amino acids 297–299) [21, 44]. These findings suggest that piR-26681 may modulate the RNA-binding specificity of the METTL3–METTL14 complex, potentially directing it toward tumor-suppressor targets. Recent work further demonstrated that mutations affecting this interface can reprogram the RNA recognition preference of the METTL3–METTL14 complex, shifting its preferred methylation motif from the canonical GGAC sequence to an alternative GGAU motif and thereby reshaping the global m^6^A landscape [45]. These findings suggest that piR-26681 may similarly influence the RNA-binding properties of the METTL3–METTL14 complex and alter its transcript targeting profile.

Furthermore, our results demonstrate that piR-26681 enhances m^6^A methylation of FBXO16, thereby increasing the stability of its mRNA. Mechanistically, the m^6^A reader IGF2BP2 recognizes the m^6^A-modified FBXO16 transcripts and promotes their stabilization, forming a piR-26681–METTL3/METTL14–IGF2BP2 regulatory axis that elevates FBXO16 expression. Given that FBXO16 exerts its function mainly through ubiquitin-mediated degradation of downstream substrates, we investigated its targets and identified MORF4L1 as a key effector. While the involvement of additional E3 ligases cannot be entirely ruled out, our interaction and functional analyses indicate that FBXO16 serves as the principal E3 ligase mediating MORF4L1 ubiquitination in ovarian cancer cells.

PARP inhibitors are a mainstay in ovarian cancer therapy, exerting their effects by blocking PARP-mediated DNA repair [46]. However, individuals with homologous recombination deficiency (HRD)-negative condition have exhibited lower PARPi efficacy [47, 48]. Fortunately, the piR-26681 effectively targets the METTL3/METTL14 complex, facilitating m^6^A modification of FBXO16 mRNA and enhancing the ubiquitination of MORF4L1 proteins, which play a role in DNA damage repair within the PALB2–BRCA2 complex. Ultimately resulting in functional impairments in homologous recombination repair. PiR-26681 may be able to increase the efficacy of PARP inhibitors in the future. Previous studies have shown that alterations in m^6^A methyltransferases can change their transcript recognition profiles, thereby influencing DNA repair and homologous recombination processes [16]. Importantly, the functional consequences of m^6^A regulation largely depend on the specific transcripts that are selectively recognized and modified. In our study, piR-26681 rewires the functional output of the METTL3–METTL14 complex toward the FBXO16–MORF4L1 axis, resulting in MORF4L1 degradation, impaired homologous recombination repair, and accumulation of DNA damage. Thus, the biological consequences of m^6^A regulation appear to be highly transcript-specific and context-dependent in ovarian cancer.

Despite piR-26681’s efficacy as a therapeutic target, RNA therapeutics grapple with challenges in clinical success due to issues with delivery systems. Efforts to improve stability and efficacy include chemical modifications and novel delivery methods. However, such chemical modifications could increase the risk of off-target effects by altering their structure, folding, biological activity, and safety of synthesized RNA molecules [49, 50]. Nanodelivery technologies, utilizing natural compounds such as gelatin, chitosan, and polyphenols, have shown the potential to decrease drug toxicity, enhance bioavailability, and provide tailored drug delivery [51]. The combination of piR-26681 with a nano-delivery system is promising and could revolutionize treatment options for ovarian cancer by improving drug efficacy and reducing side effects. Further research and clinical trials are needed to fully explore the benefits of integrating piR-26681 with these innovative delivery technologies in the treatment of ovarian cancer.

In our study, we found that piR-26681 expression correlated with early FIGO stages and a good prognosis, inhibiting malignant tendencies in ovarian cancer, as confirmed in both ovarian cancer cell lines and xenograft models, as well as in patient-derived organoids. These observations suggest that piR-26681 may play a protective role during ovarian cancer progression. However, it remains unclear whether the decrease of piR-26681 is a cause or a consequence of tumor progression. piR-26681 downregulation may both contribute to and reflect ovarian cancer progression, and future studies, such as longitudinal patient analyses or early tumorigenesis models, will be required to clarify the temporal relationship. In terms of mechanism, piR-26681 targets the METTL3/METTL14 complex and increases its stability, while facilitating their binding and increasing the levels of m^6^A methylation and increasing the stability of METTL14 and FBXO16 mRNAs. PiR-26681 inhibits the homologous recombination repair by elevating FBXO16 expression to augment the ubiquitination of MORF4L1 proteins. These findings indicate that piR-26681 represents a potentially promising strategy for improving the treatment outcomes of ovarian cancer.

## Conclusions

**Fig. 9:**
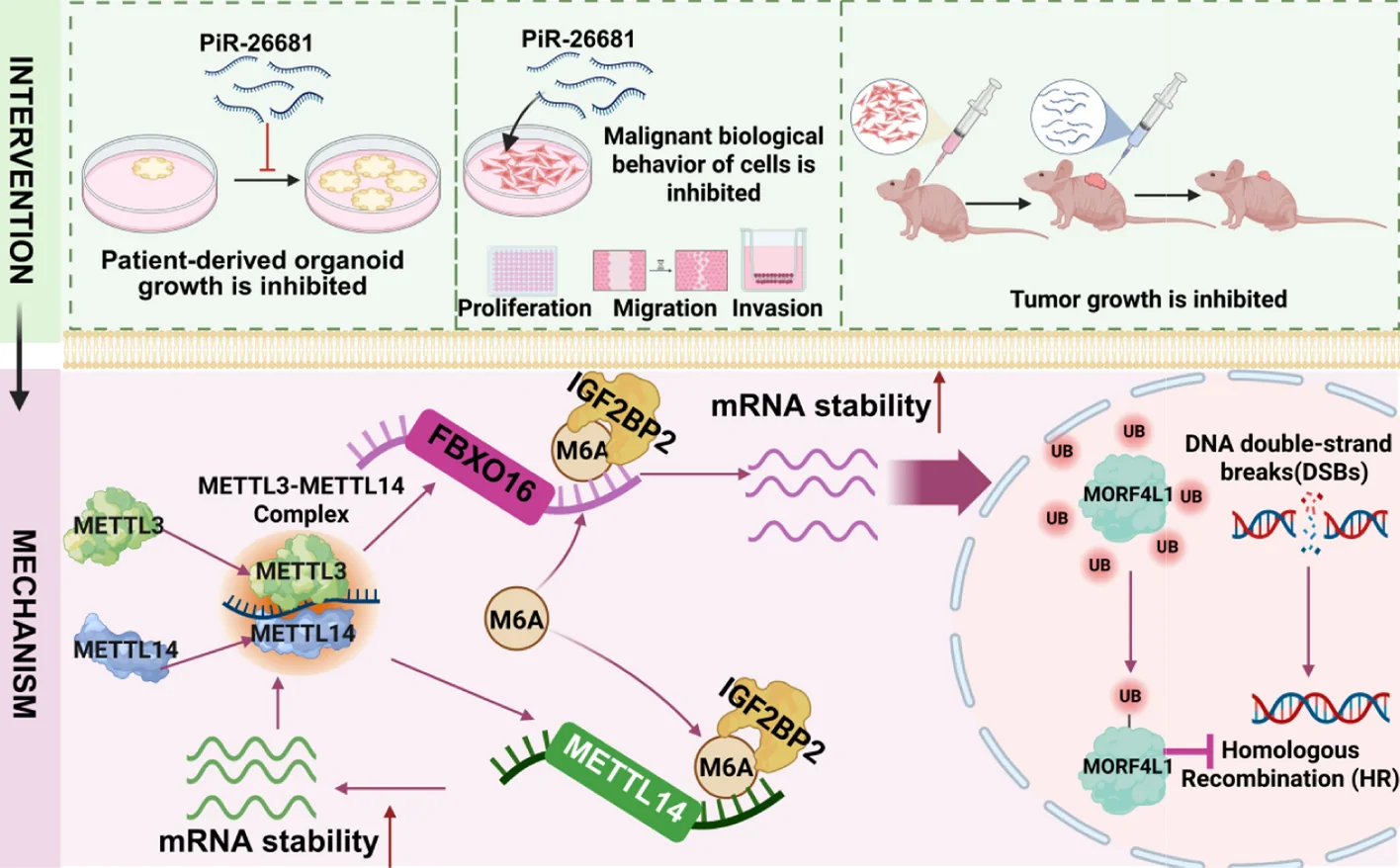
Mechanism diagram of piR-26681 in ovarian cancer. piR-26681 suppresses the aggressive behavior of ovarian cancer cells, patient-derived organoids, and xenograft models. Mechanistically, piR-26681 interacts with METTL3 and METTL14, promoting their stability and interaction, leading to increased m^6^A methylation and enhanced stability of METTL14 and FBXO16 mRNAs. This ultimately results in the downregulation of MORF4L1 protein, a key component of the PALB2–BRCA2 homologous recombination repair complex, thereby impairing DNA repair in ovarian cancer cells

PiR-26681 expression correlated with early FIGO stages and a good prognosis, inhibiting malignant tendencies in ovarian cancer, as confirmed in both ovarian cancer cell lines and xenograft models, as well as in patient-derived organoids. In terms of mechanism, piR-26681 targets the METTL3/METTL14 complex and increases its stability, while facilitating their binding and increasing the levels of m^6^A methylation and increasing the stability of METTL14 and FBXO16 mRNAs. PiR-26681 inhibits the homologous recombination repair by elevating FBXO16 expression to augment the ubiquitination of MORF4L1 proteins (Fig. 9). These findings indicate that piR-26681 represents a potentially promising strategy for improving the treatment outcomes of ovarian cancer.

## Supplementary Information

Additional file 1.

Additional file 2.

Additional file 3.

## Data Availability

All data needed to evaluate the conclusions in the paper are presented in the paper. The resources, tools, and codes used in our analyses were described in the methods section. For any further data requests, please contact the corresponding author.

## Abbreviations

PIWI: P-element-induced wimpy testis interacting
piRNAs: P-element-induced wimpy testis interacting RNAs
MORF4L1: Mortality factor 4 like 1
METTL3: Methyltransferase-like 3
METTL14: Methyltransferase-like 14
FBXO16: F-box protein 16
PARP: Polyadenosine diphosphate ribose polymerase
HRR: Homologous recombination repair
PDOs: Patient-derived organoids
shRNA: Short-hairpin RNA
ActD: Actinomycin D
CCK-8: Cell Counting Kit-8
EdU assay: 5-Ethynyl-2′-deoxyuridine assay
RIP: RNA Immunoprecipitation
FISH: Fluorescence In Situ Hybridization
COIP: Co-immunoprecipitation
