# Supplementary material for: piR-26681 suppresses ovarian cancer progression by enhancing METTL3/METTL14-mediated m6A modification of FBXO16 and impairing DNA repair via MORF4L1 degradation
Source: Cell Mol Biol Lett. 2026 Jun 24;31:143. doi: 10.1186/s11658-026-00969-x (PMC13560453; doi:10.1186/s11658-026-00969-x)

Figure 3A

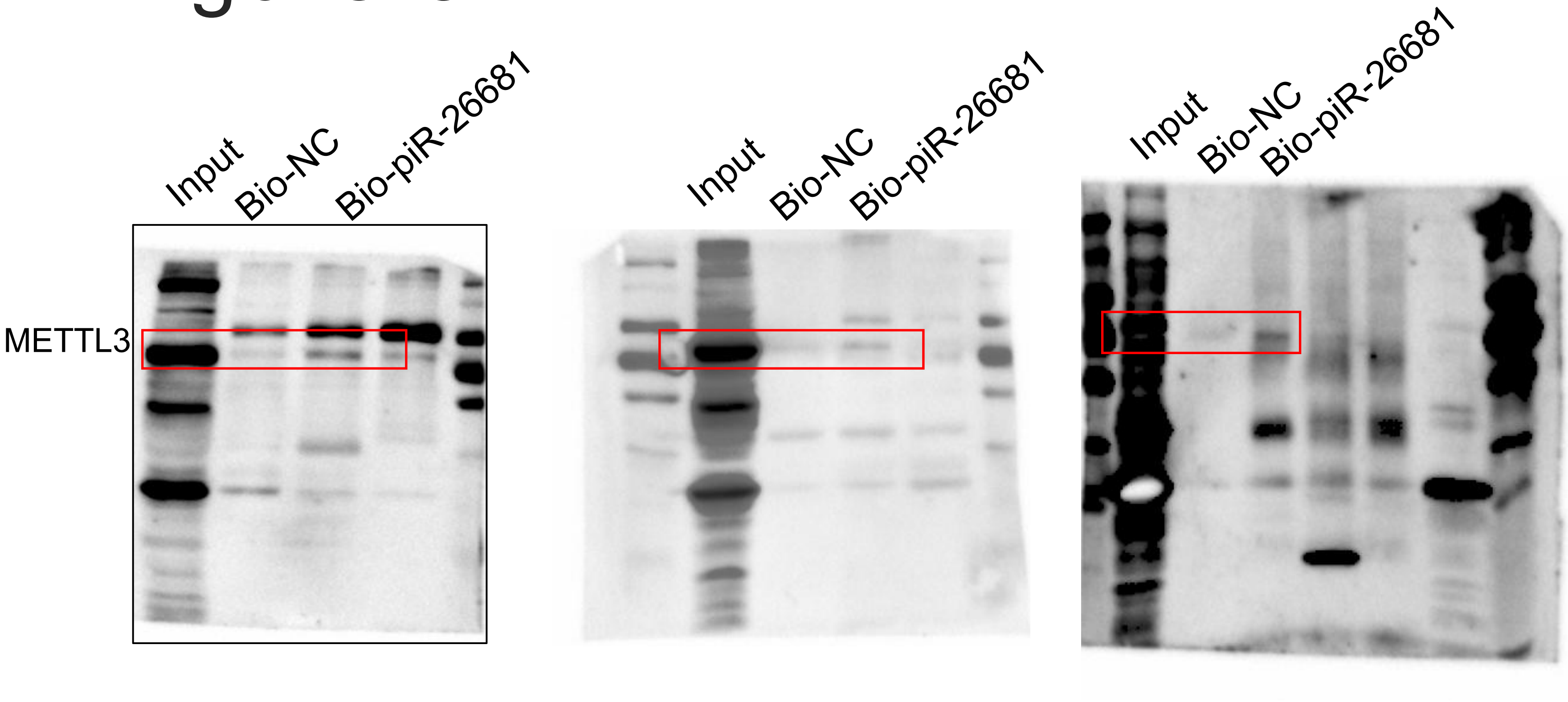
Input
Bio-NC
Bio-piR-26681
METTL3
Input
Bio-NC
Bio-piR-26681
Input
Bio-NC
Bio-piR-26681

Figure 3A

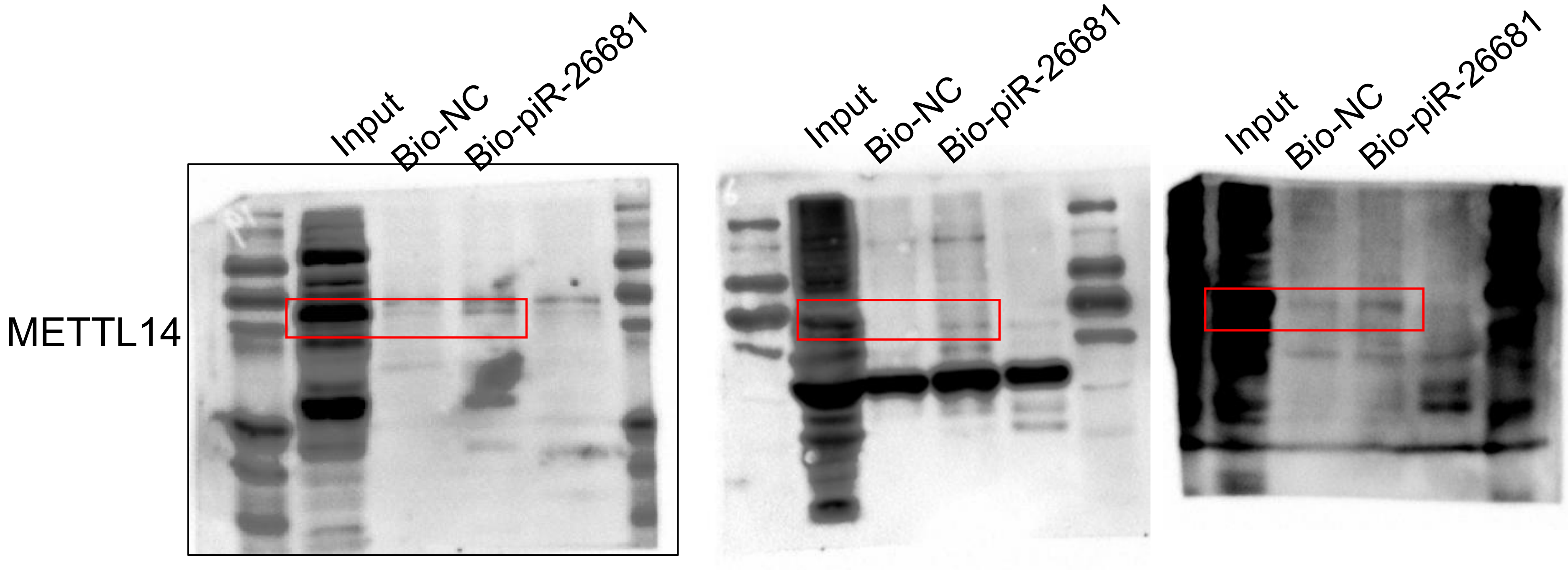

Input
Bio-NC
Bio-piR-26681
METTL14
Input
Bio-NC
Bio-piR-26681
Input
Bio-NC
Bio-piR-26681

Figure 3G

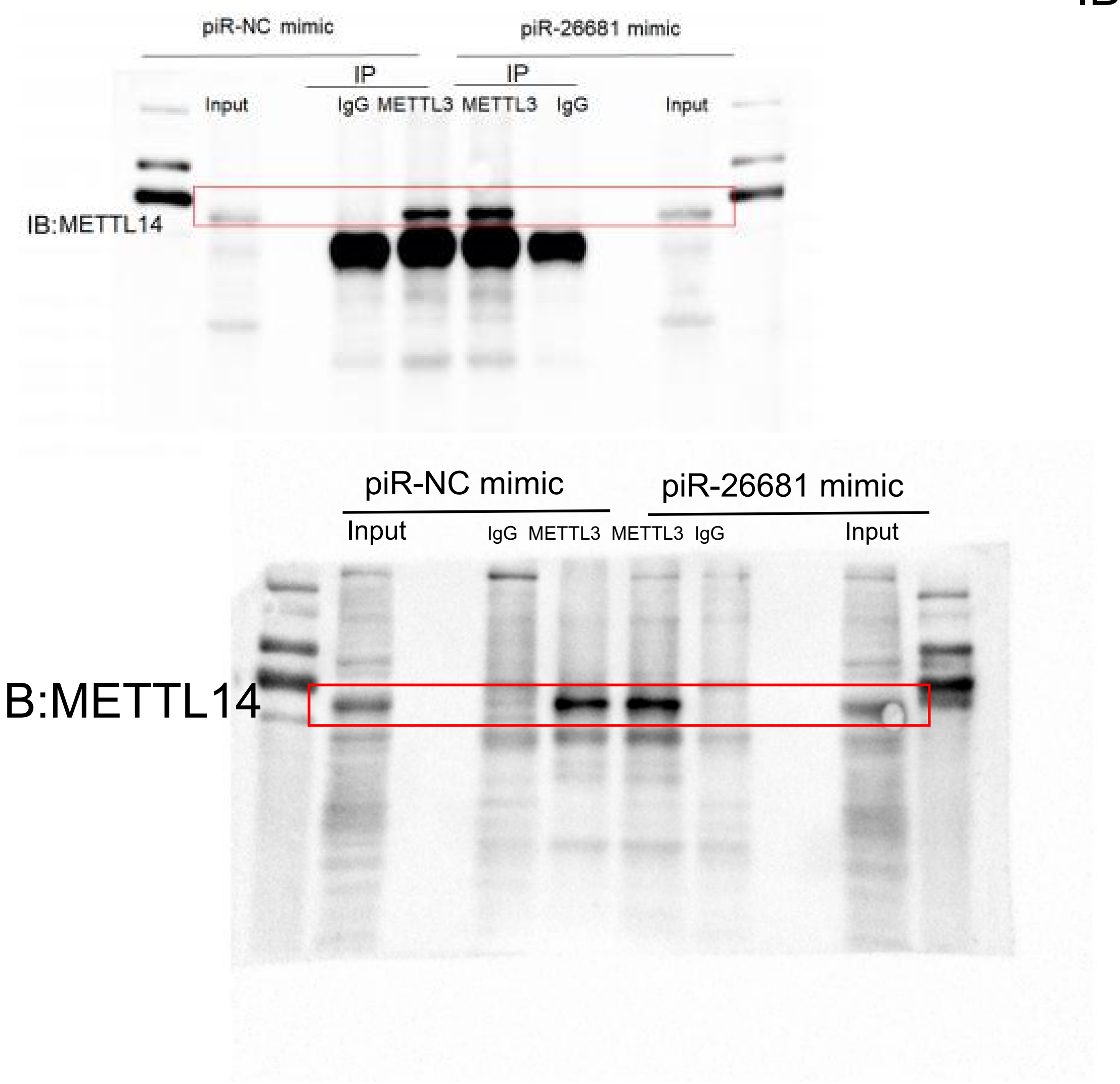
piR-NC mimic
piR-26681 mimic
IP
IP
Input
IgG METTL3 METTL3 IgG
Input
IB:METTL14
piR-NC mimic
piR-26681 mimic
Input
IgG METTL3 METTL3 IgG
Input
B:METTL14

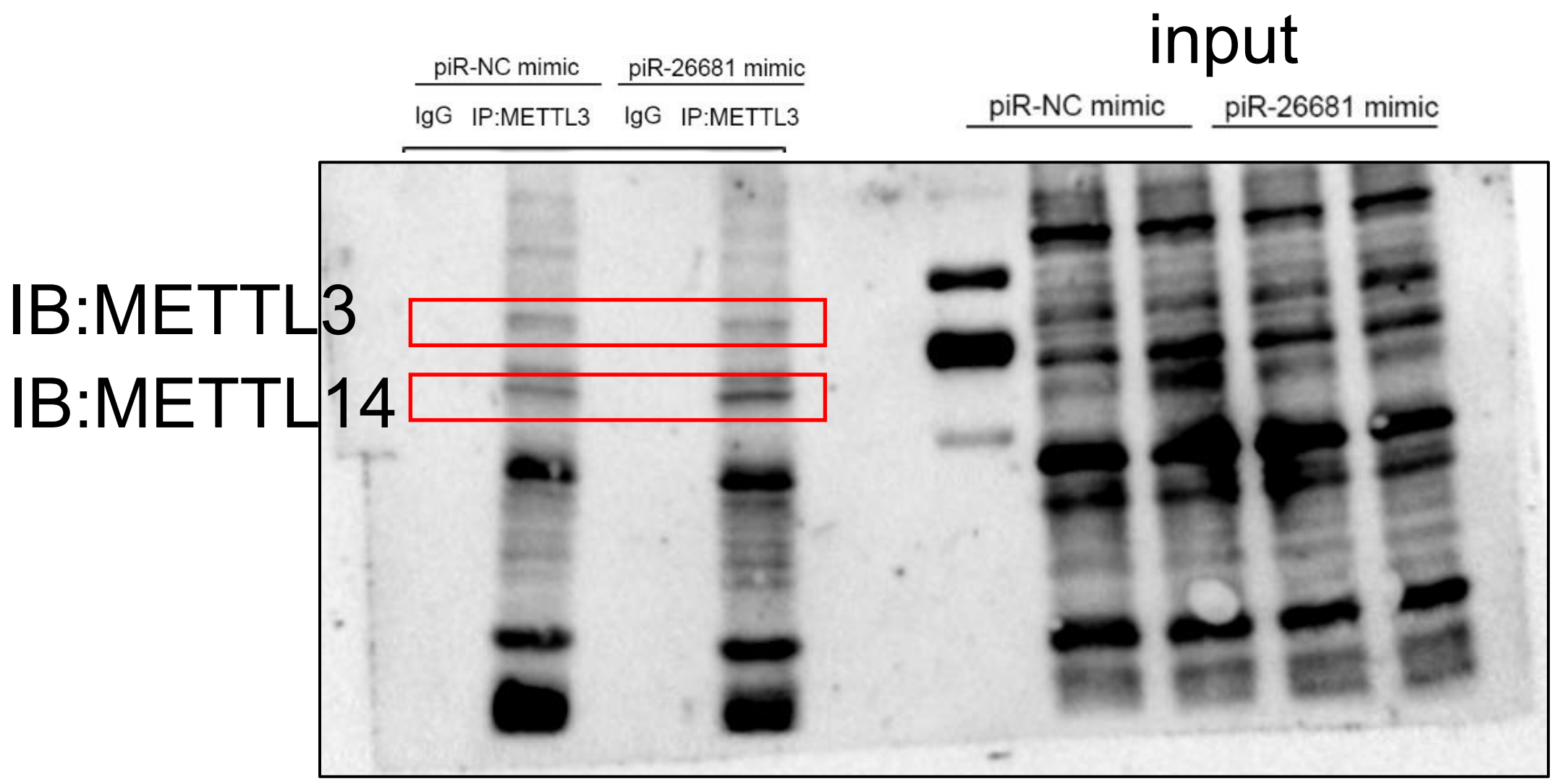
input
piR-NC mimic
piR-26681 mimic
IgG IP:METTL3
IgG IP:METTL3
piR-NC mimic
piR-26681 mimic
IB:METTL3
IB:METTL14

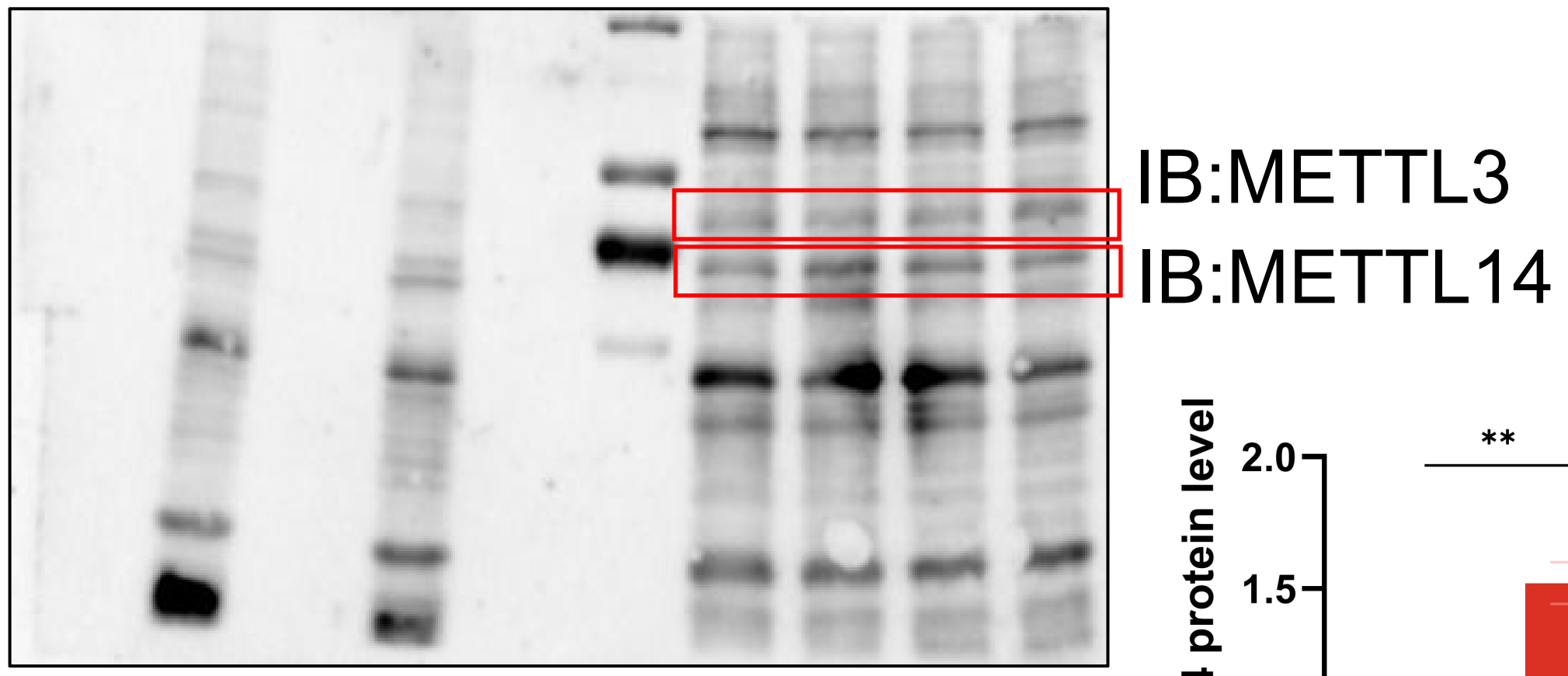
IB:METTL3
IB:METTL14

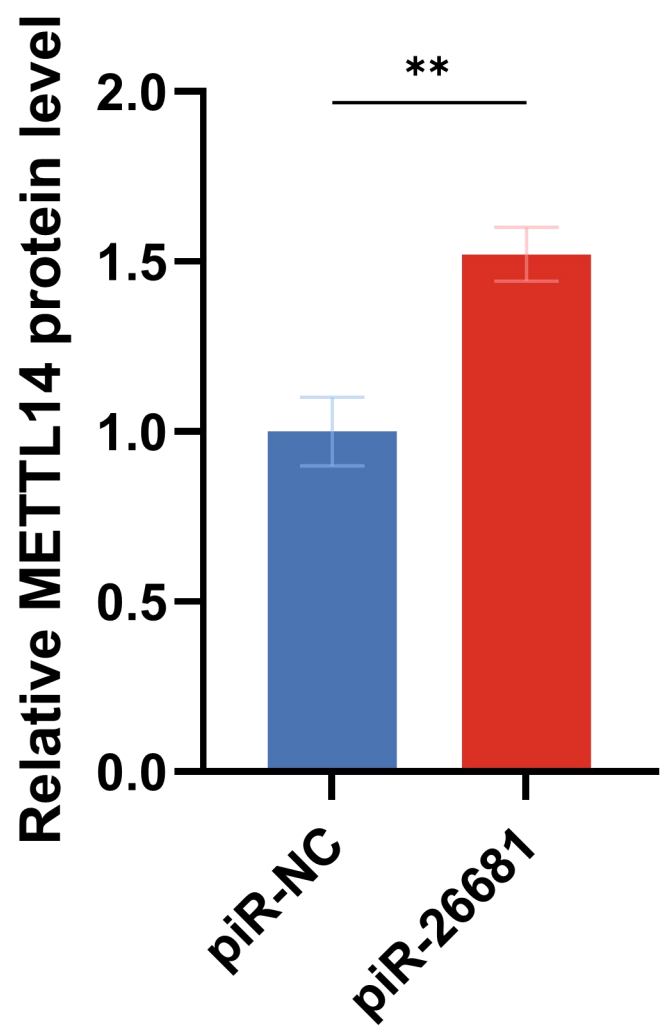
Relative METTL14 protein level
2.0
1.5
1.0
0.5
0.0
**
piR-NC
piR-26681

Figure 3H

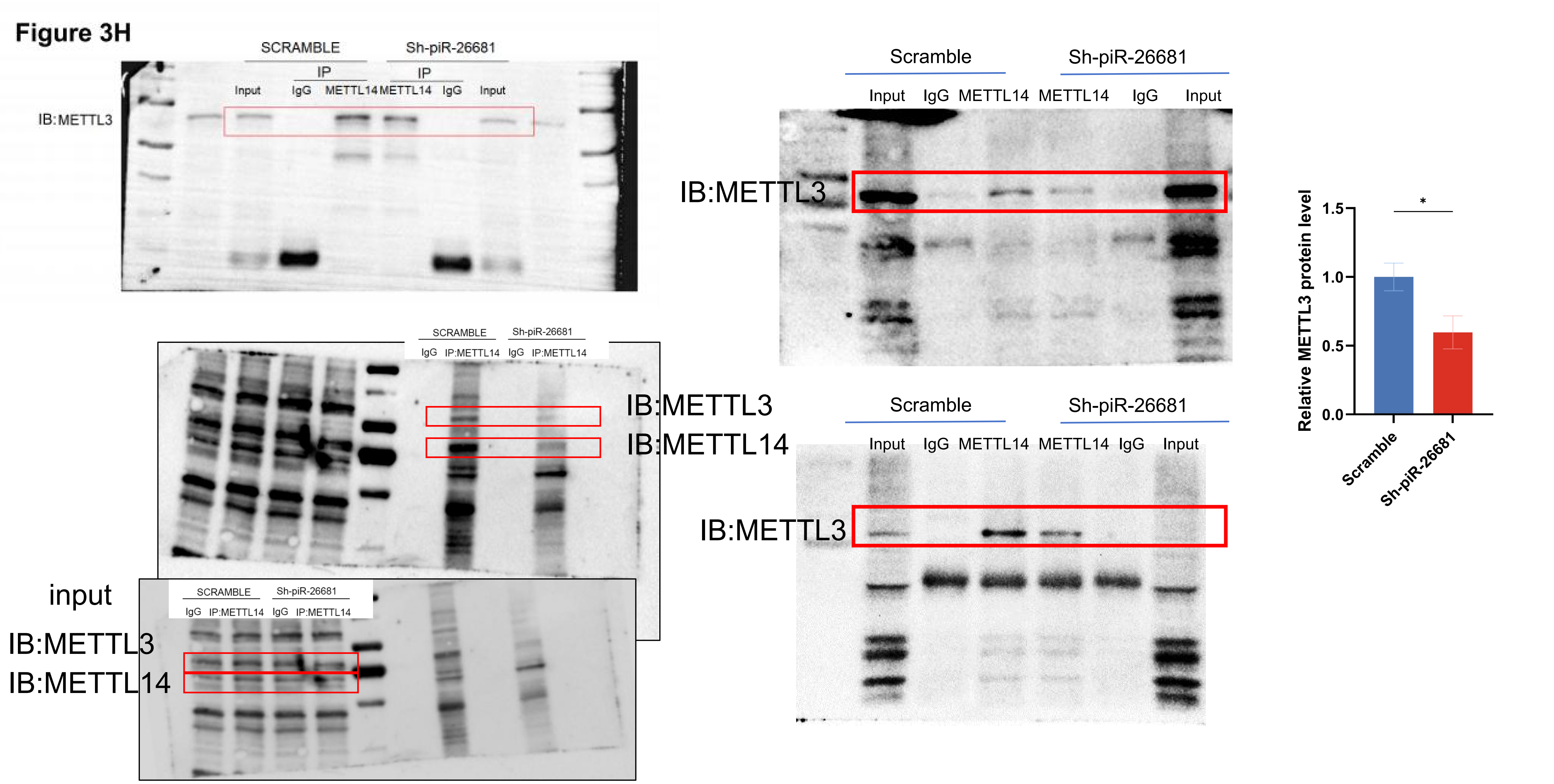
Figure 3H
SCRAMBLE
Sh-piR-26681
IP
IP
Input
IgG
METTL14
METTL14
IgG
Input
IB:METTL3
SCRAMBLE
Sh-piR-26681
IgG
IP:METTL14
IgG
IP:METTL14
IB:METTL3
IB:METTL14
input
SCRAMBLE
Sh-piR-26681
IgG
IP:METTL14
IgG
IP:METTL14
IB:METTL3
IB:METTL14
Scramble
Sh-piR-26681
Input
IgG
METTL14
METTL14
IgG
Input
IB:METTL3
Scramble
Sh-piR-26681
Input
IgG
METTL14
METTL14
IgG
Input
IB:METTL3
Relative METTL3 protein level
1.5
1.0
0.5
0.0
*
Scramble
Sh-piR-26681

Figure 3I

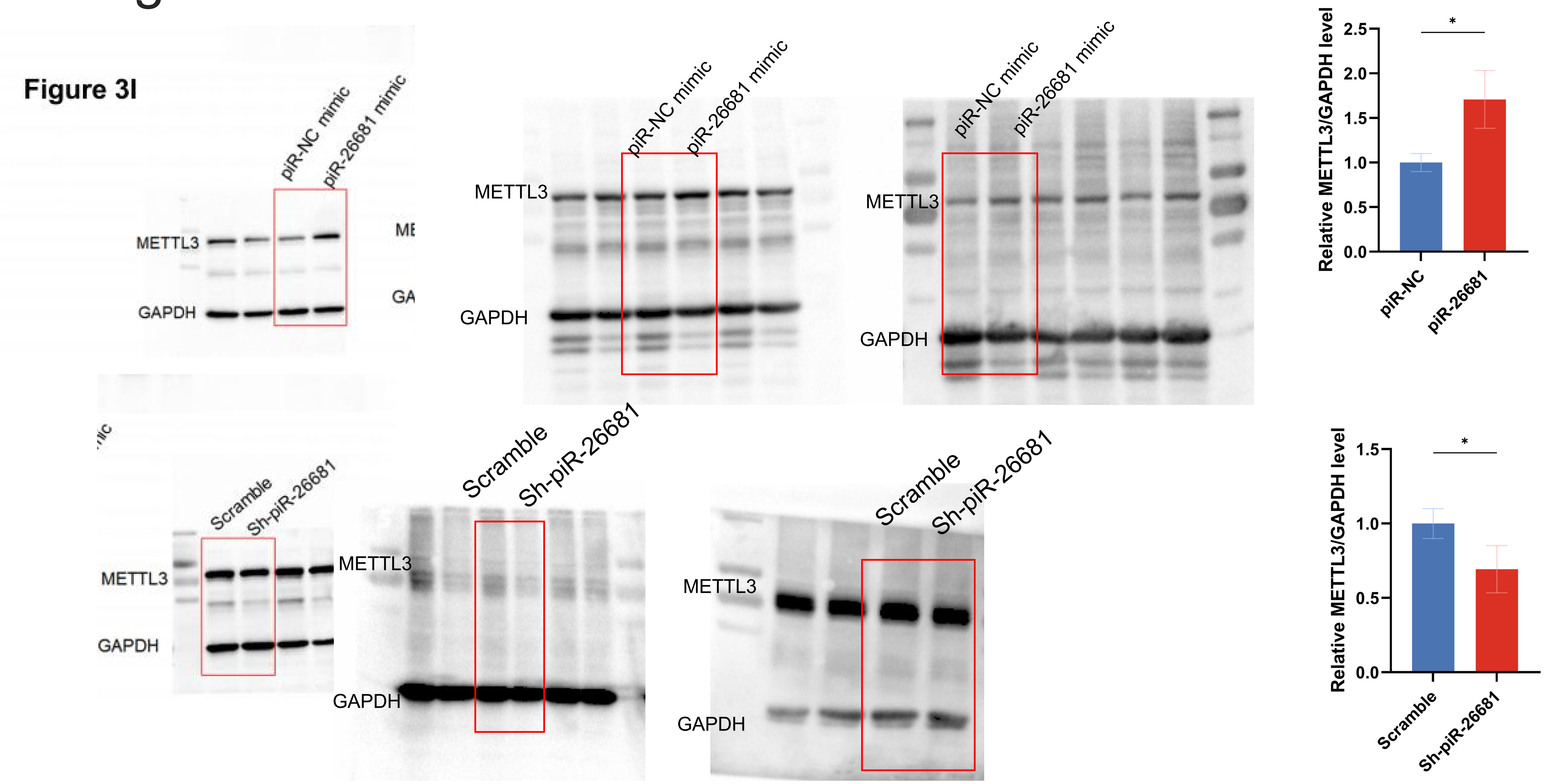
Figure 3I
piR-NC mimic
piR-26681 mimic
METTL3
GAPDH
Scramble
Sh-piR-26681
Relative METTL3/GAPDH level
2.5
2.0
1.5
1.0
0.5
0.0
*
piR-NC
piR-26681
Scramble
Sh-piR-26681

Figure 3k

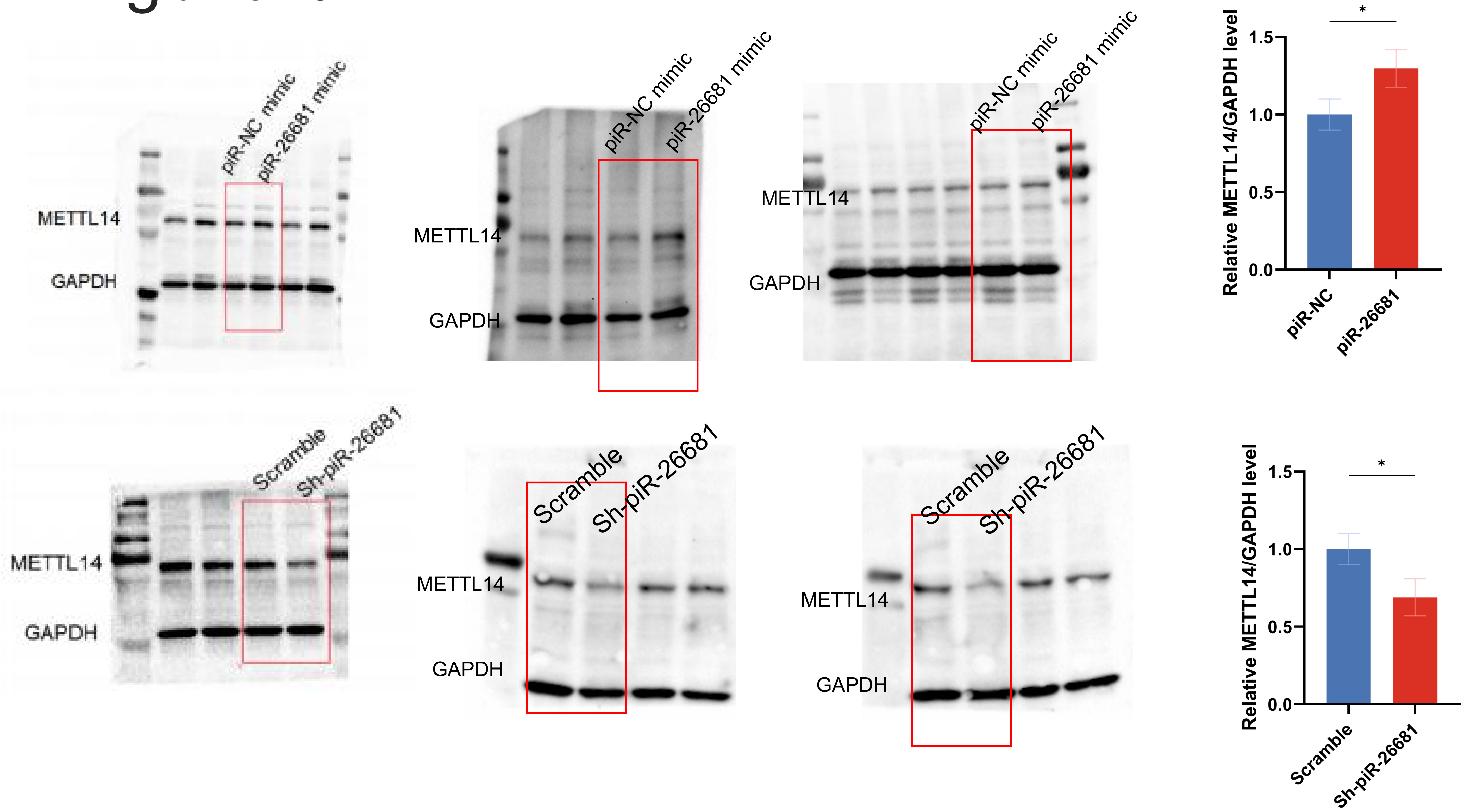

piR-NC mimic
piR-26681 mimic
METTL14
GAPDH
piR-NC mimic
piR-26681 mimic
METTL14
GAPDH
piR-NC mimic
piR-26681 mimic
METTL14
GAPDH
Relative METTL14/GAPDH level
1.5
1.0
0.5
0.0
*
piR-NC
piR-26681
Scramble
Sh-piR-26681
METTL14
GAPDH
Scramble
Sh-piR-26681
METTL14
GAPDH
Scramble
Sh-piR-26681
METTL14
GAPDH
Relative METTL14/GAPDH level
1.5
1.0
0.5
0.0
*
Scramble
Sh-piR-26681

Figure 3L

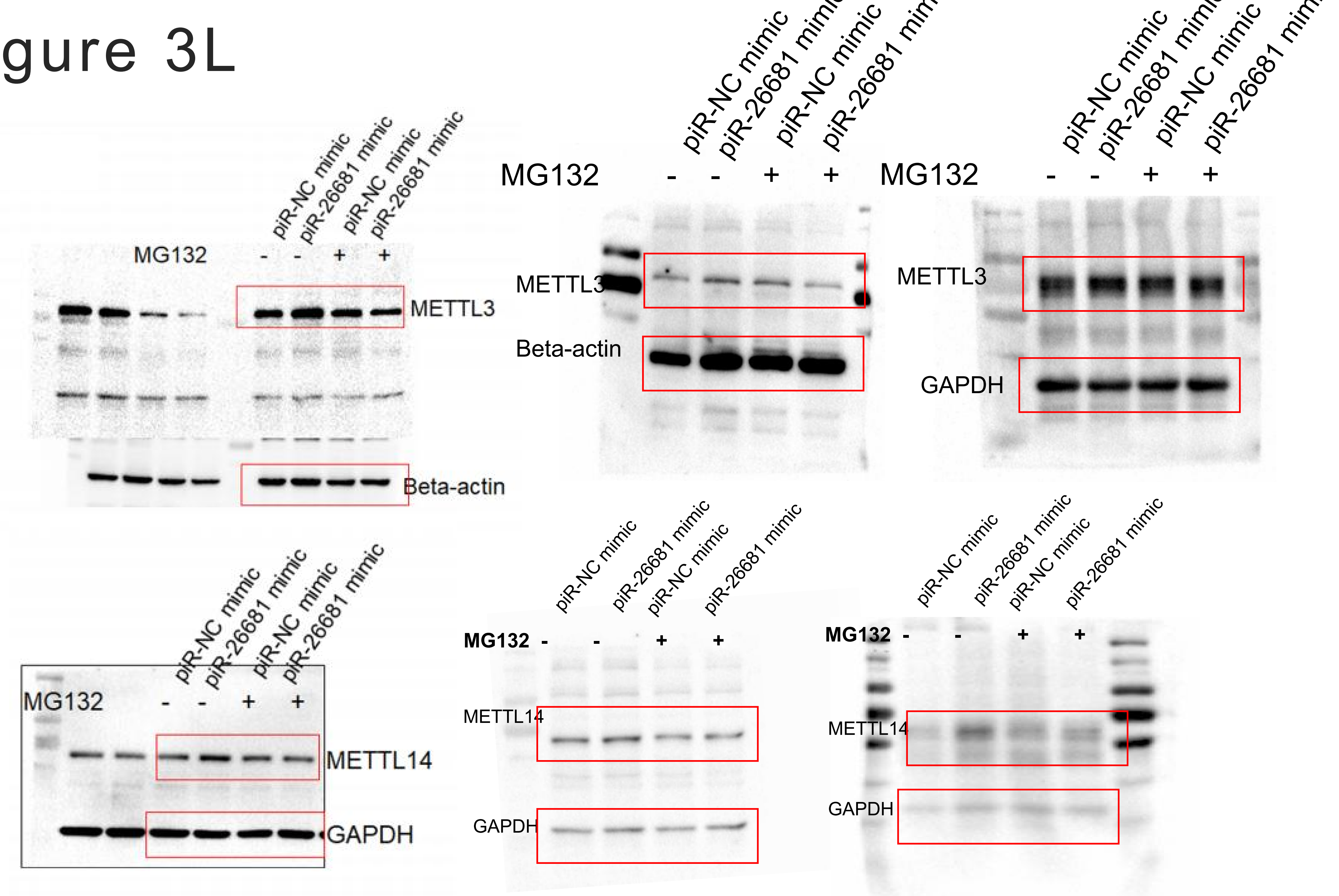

piR-NC mimic
piR-26681 mimic
piR-NC mimic
piR-26681 mimic
MG132
-
-
+
+
METTL3
Beta-actin
GAPDH
METTL14

Figure 3M

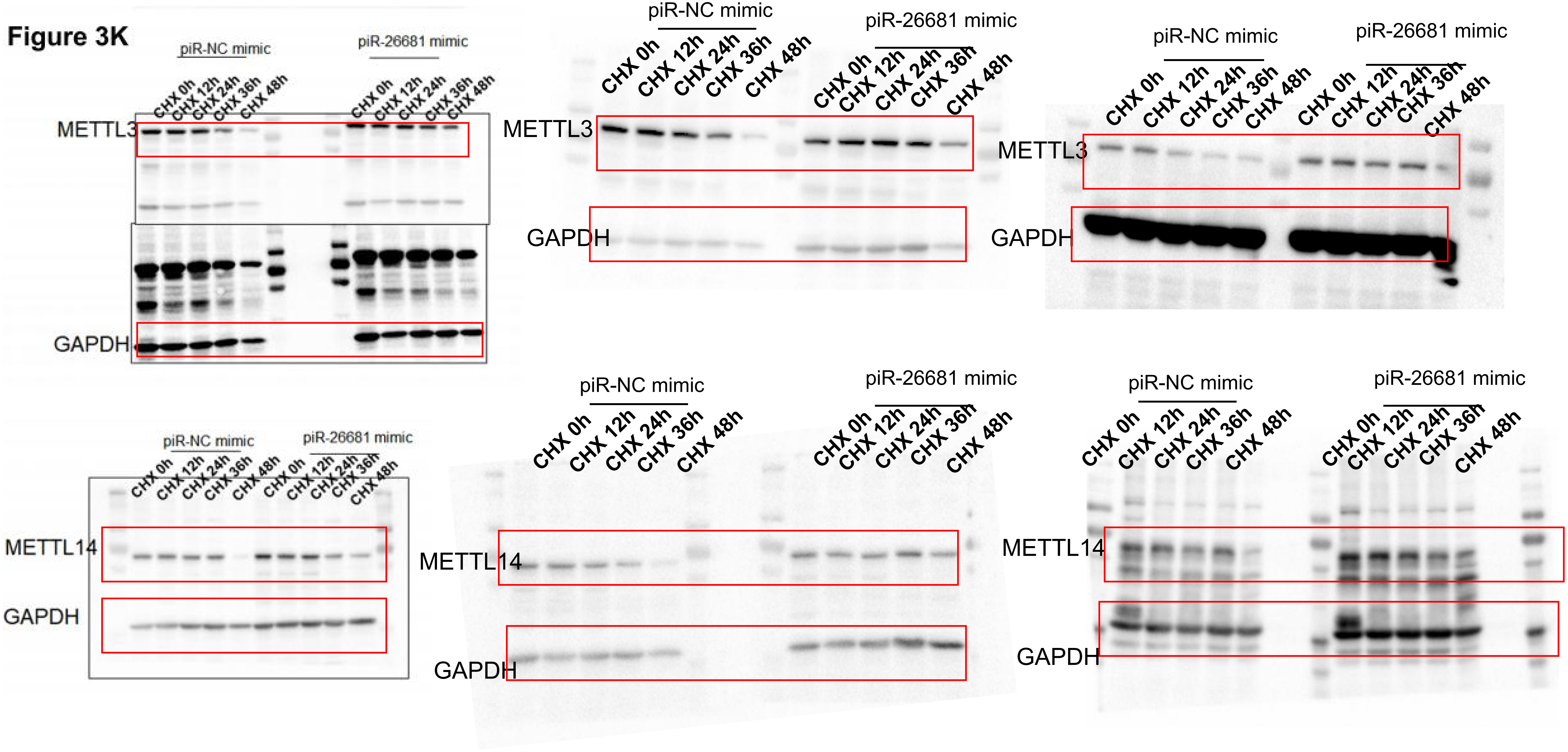
Figure 3K
piR-NC mimic
piR-26681 mimic
CHX 0h
CHX 12h
CHX 24h
CHX 36h
CHX 48h
METTL3
GAPDH
METTL14

Figure 3N

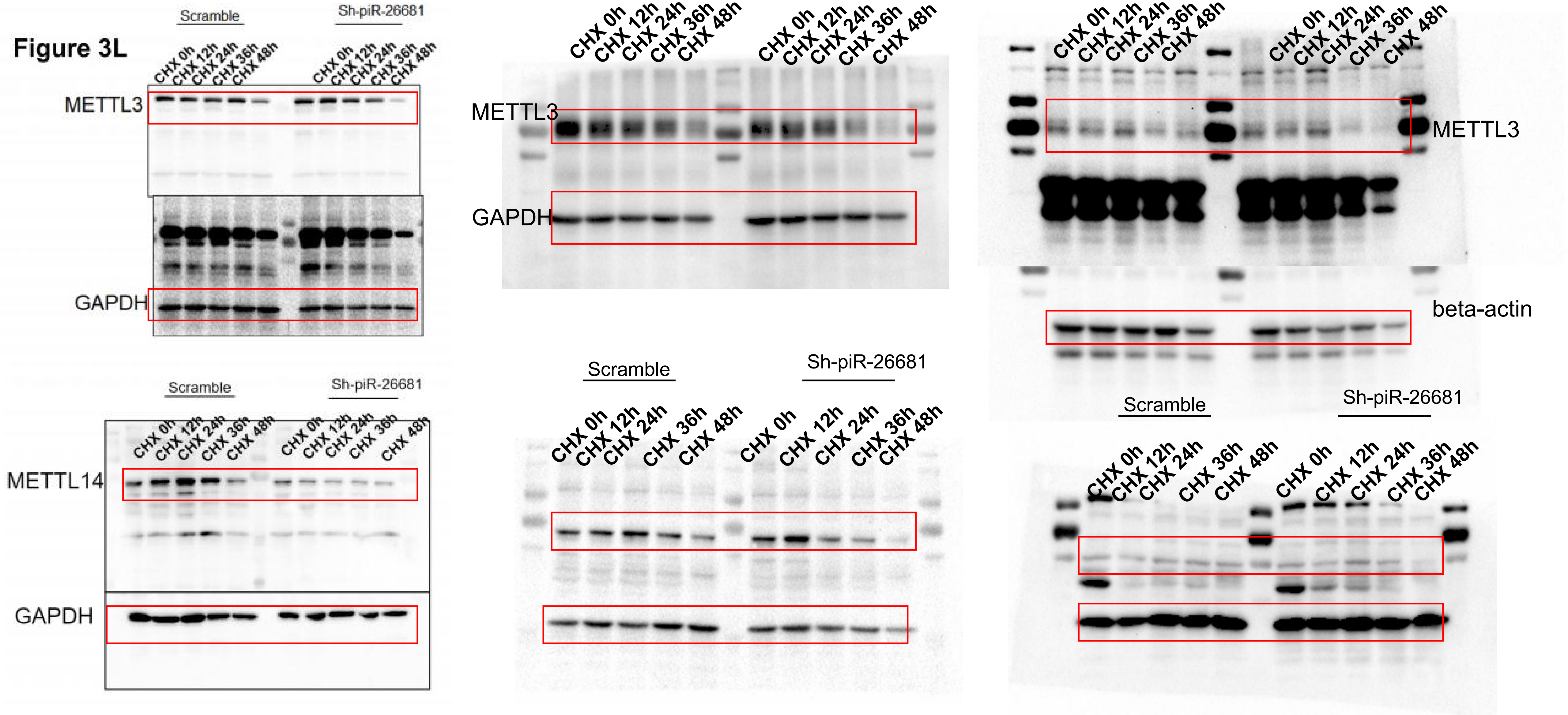
Figure 3L
Scramble
Sh-piR-26681
CHX 0h
CHX 12h
CHX 24h
CHX 36h
CHX 48h
METTL3
GAPDH
METTL14
beta-actin

Figure 4N

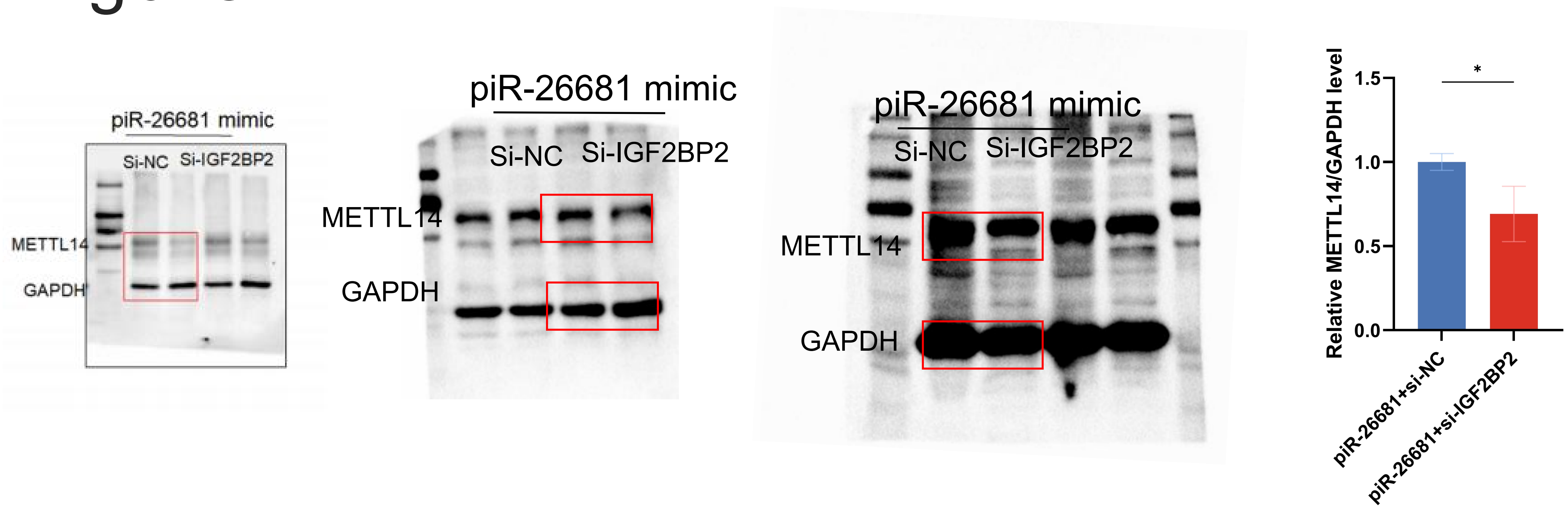
piR-26681 mimic
Si-NC
Si-IGF2BP2
METTL14
GAPDH
piR-26681 mimic
Si-NC
Si-IGF2BP2
METTL14
GAPDH
piR-26681 mimic
Si-NC
Si-IGF2BP2
METTL14
GAPDH
Relative METTL14/GAPDH level
1.5
1.0
0.5
0.0
*
piR-26681+si-NC
piR-26681+si-IGF2BP2

Figure5D

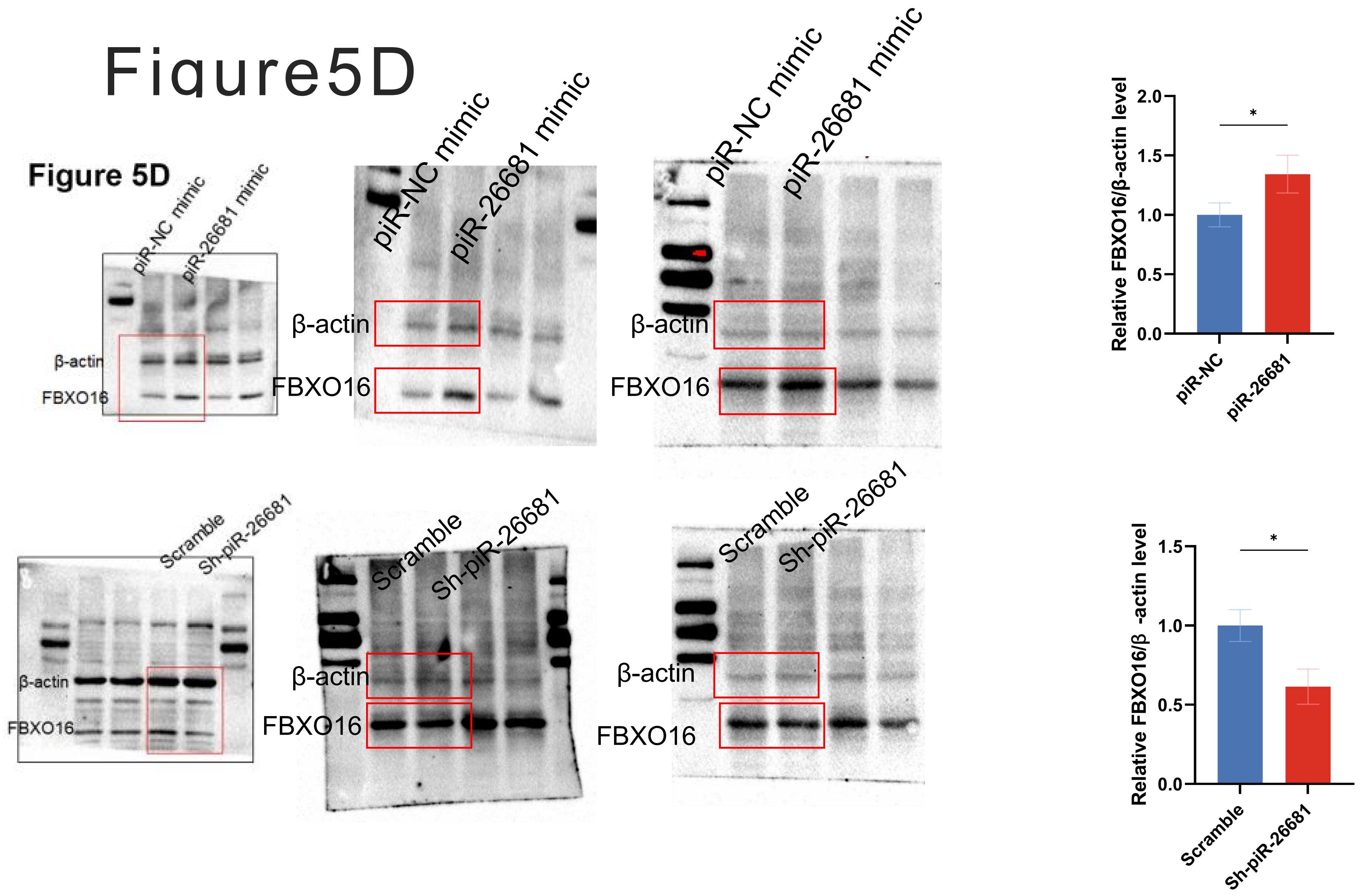

Figure 5D
piR-NC mimic
piR-26681 mimic
β-actin
FBXO16
piR-NC mimic
piR-26681 mimic
β-actin
FBXO16
piR-NC mimic
piR-26681 mimic
β-actin
FBXO16
Relative FBXO16/β-actin level
2.0
1.5
1.0
0.5
0.0
*
piR-NC
piR-26681
Scramble
Sh-piR-26681
β-actin
FBXO16
Scramble
Sh-piR-26681
β-actin
FBXO16
Scramble
Sh-piR-26681
β-actin
FBXO16
Relative FBXO16/β -actin level
1.5
1.0
0.5
0.0
*
Scramble
Sh-piR-26681

Figure5I

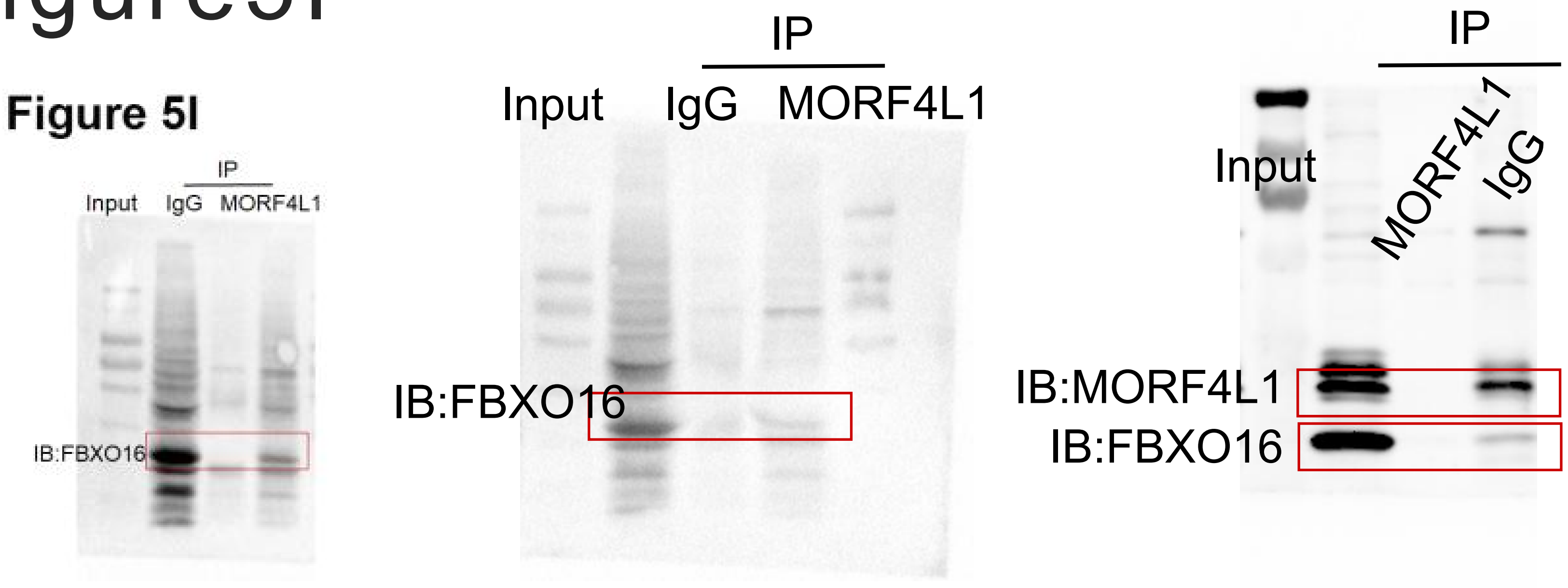

Figure 5I
IP
Input
IgG
MORF4L1
IB:FBXO16
IP
Input
IgG
MORF4L1
IB:FBXO16
IP
Input
MORF4L1
IgG
IB:MORF4L1
IB:FBXO16

Figure 5J

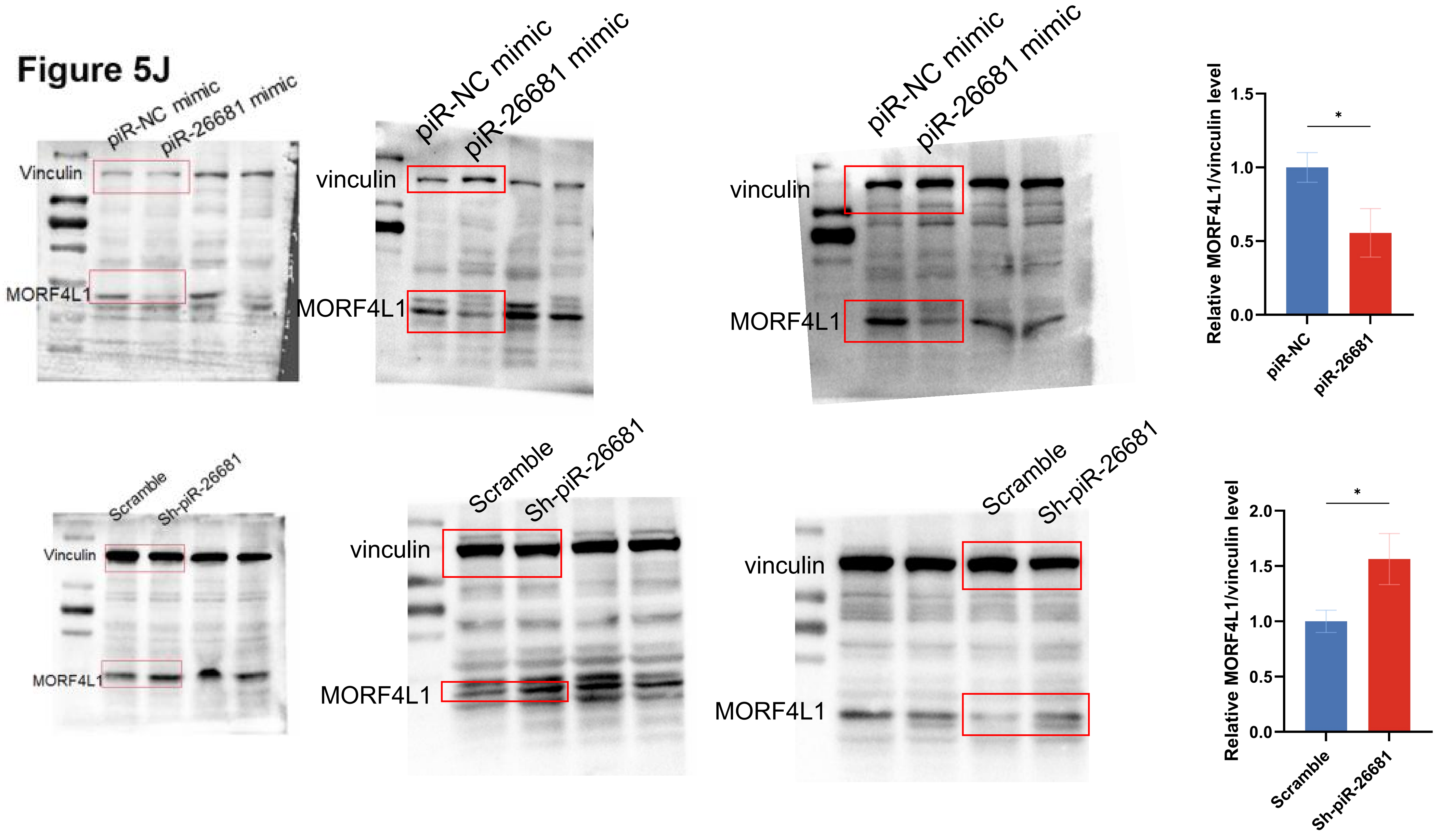
piR-NC mimic
piR-26681 mimic
Vinculin
MORF4L1
vinculin
MORF4L1
Relative MORF4L1/vinculin level
1.5
1.0
0.5
0.0
*
piR-NC
piR-26681
Scramble
Sh-piR-26681
Vinculin
MORF4L1
2.0
Scramble
Sh-piR-26681

Fig. 5K

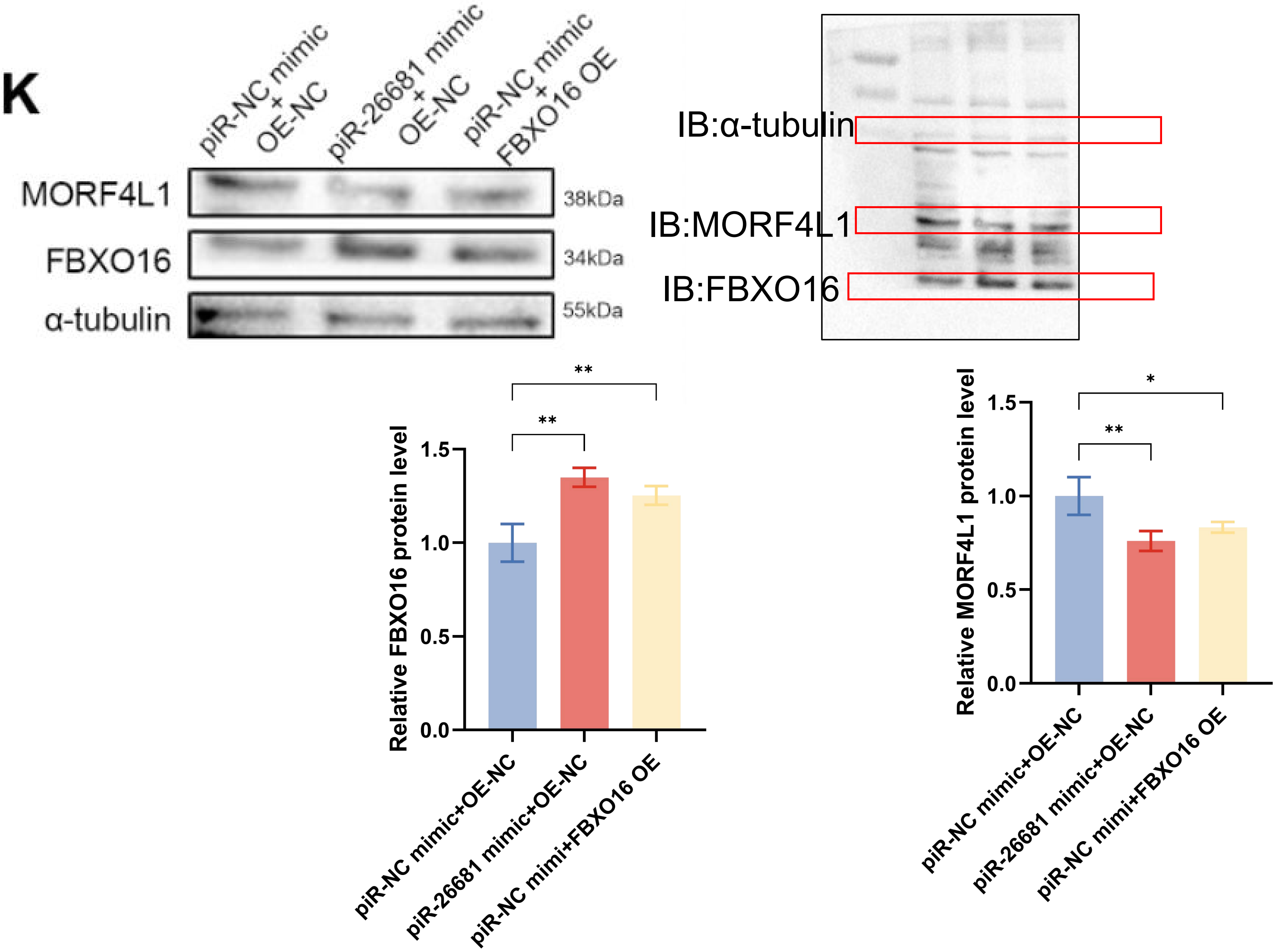
piR-NC mimic + OE-NC
piR-26681 mimic + OE-NC
piR-NC mimic + FBXO16 OE
MORF4L1
FBXO16
α-tubulin
38kDa
34kDa
55kDa
IB:α-tubulin
IB:MORF4L1
IB:FBXO16
Relative FBXO16 protein level
Relative MORF4L1 protein level
0.0
0.5
1.0
1.5
**
*
piR-NC mimic+OE-NC
piR-26681 mimic+OE-NC
piR-NC mimi+FBXO16 OE

Figure 5L

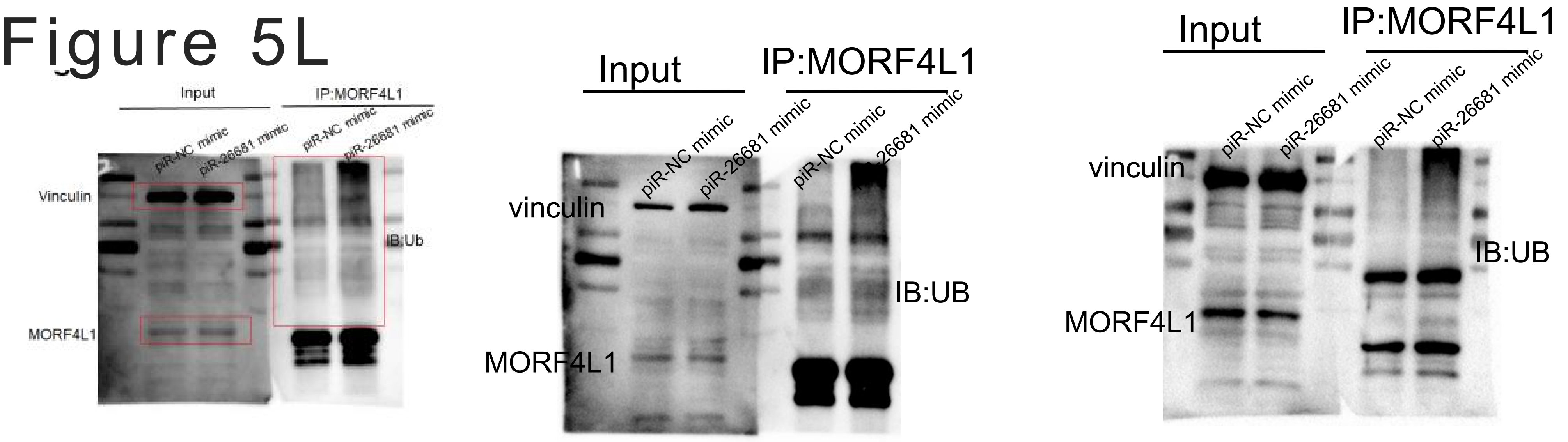

Figure 5M

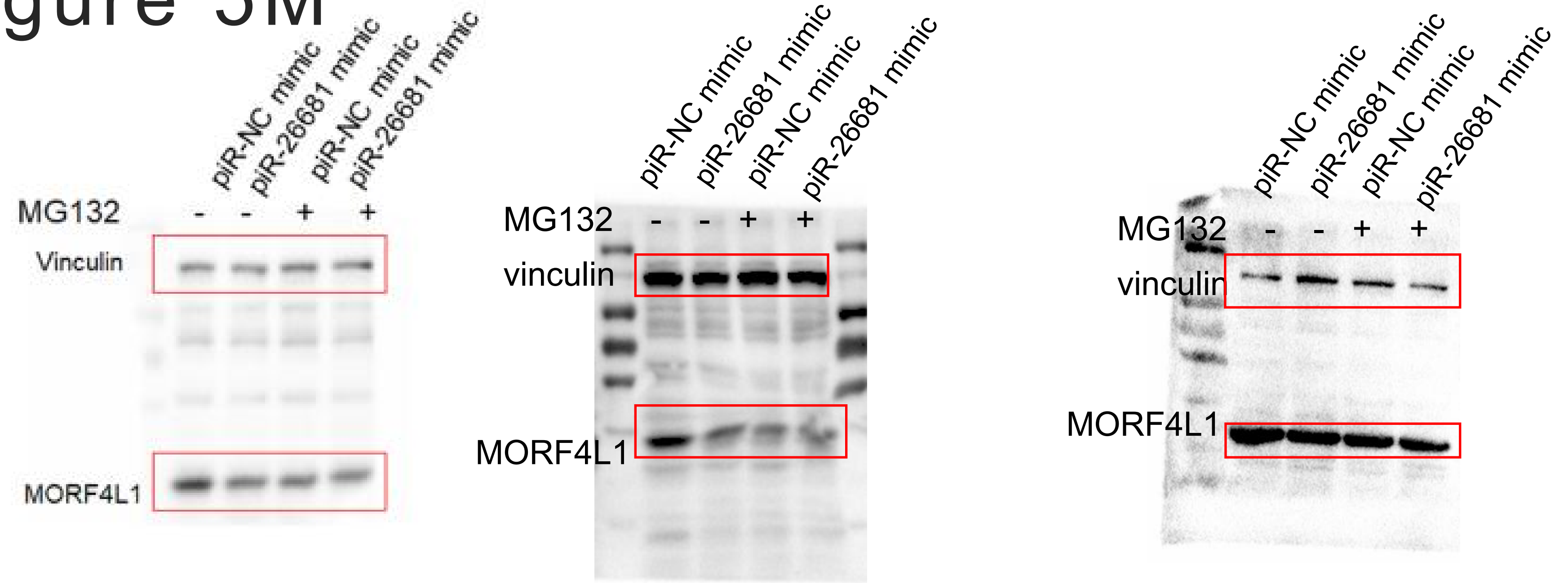

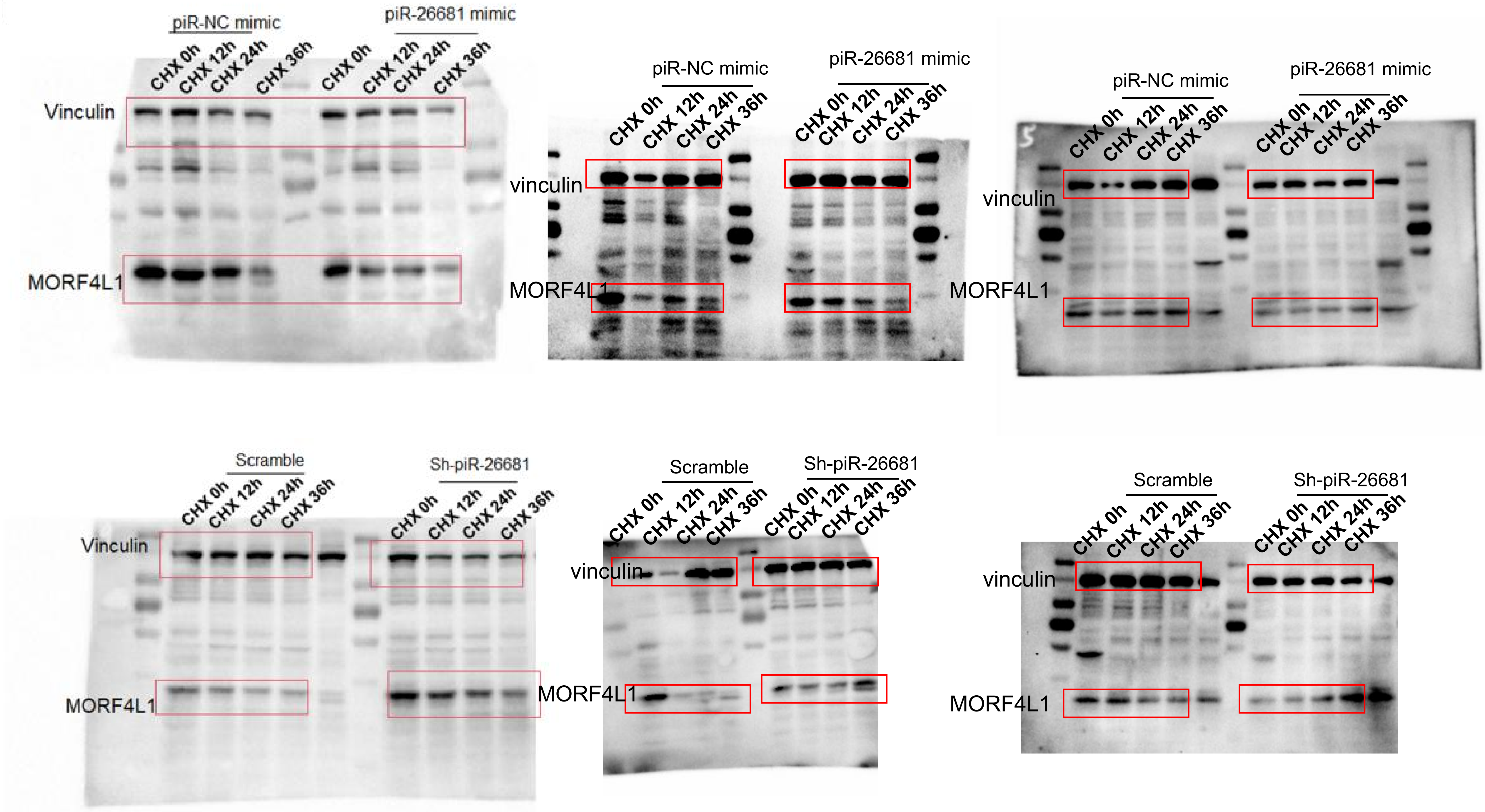
piR-NC mimic
piR-26681 mimic
CHX 0h
CHX 12h
CHX 24h
CHX 36h
Vinculin
MORF4L1
vinculin
Scramble
Sh-piR-26681

Figure 5P

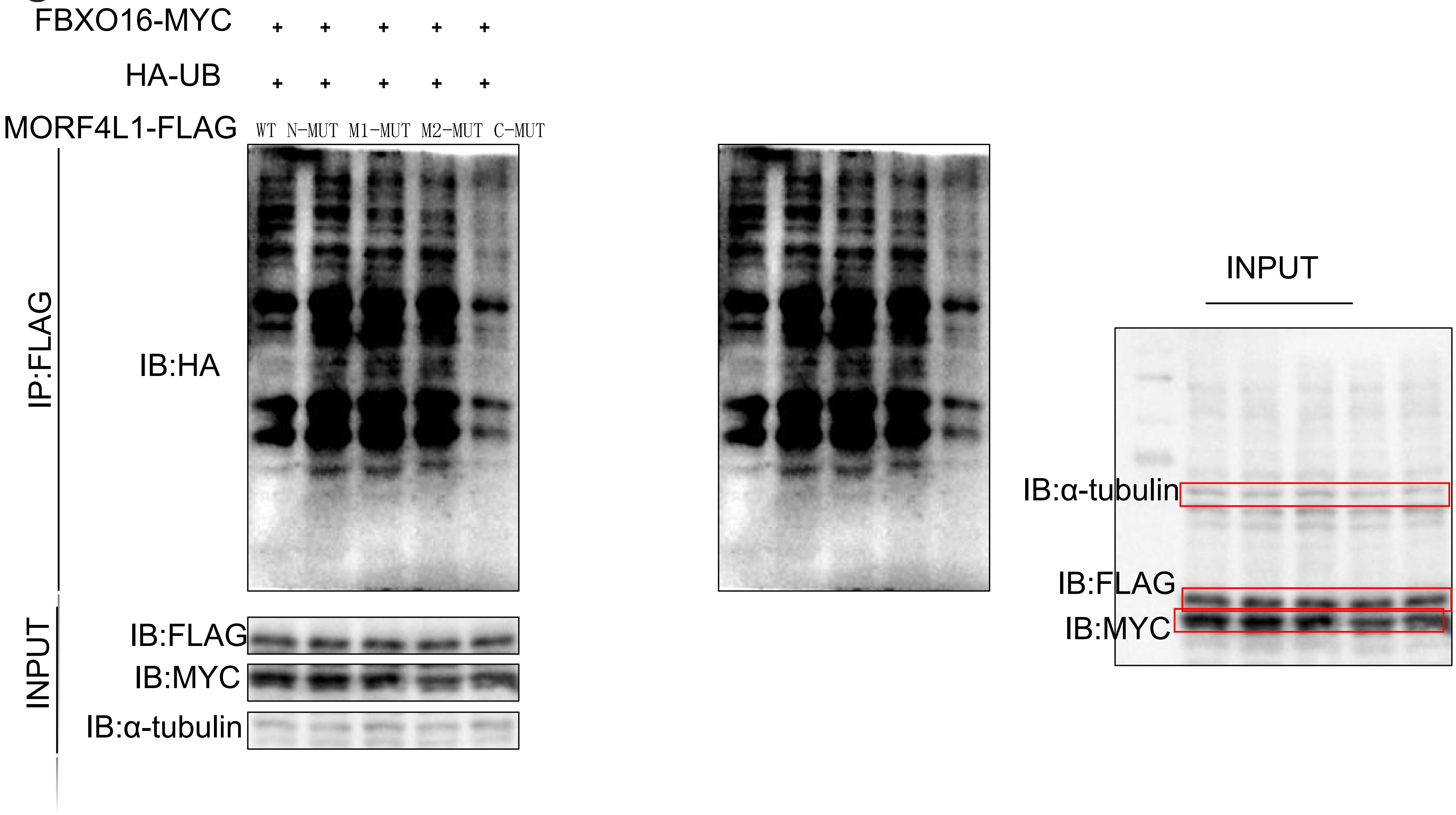
FBXO16-MYC
+ + + + +
HA-UB
+ + + + +
MORF4L1-FLAG
WT N-MUT M1-MUT M2-MUT C-MUT
IP:FLAG
IB:HA
INPUT
IB:FLAG
IB:MYC
IB:α-tubulin
INPUT
IB:α-tubulin
IB:FLAG
IB:MYC

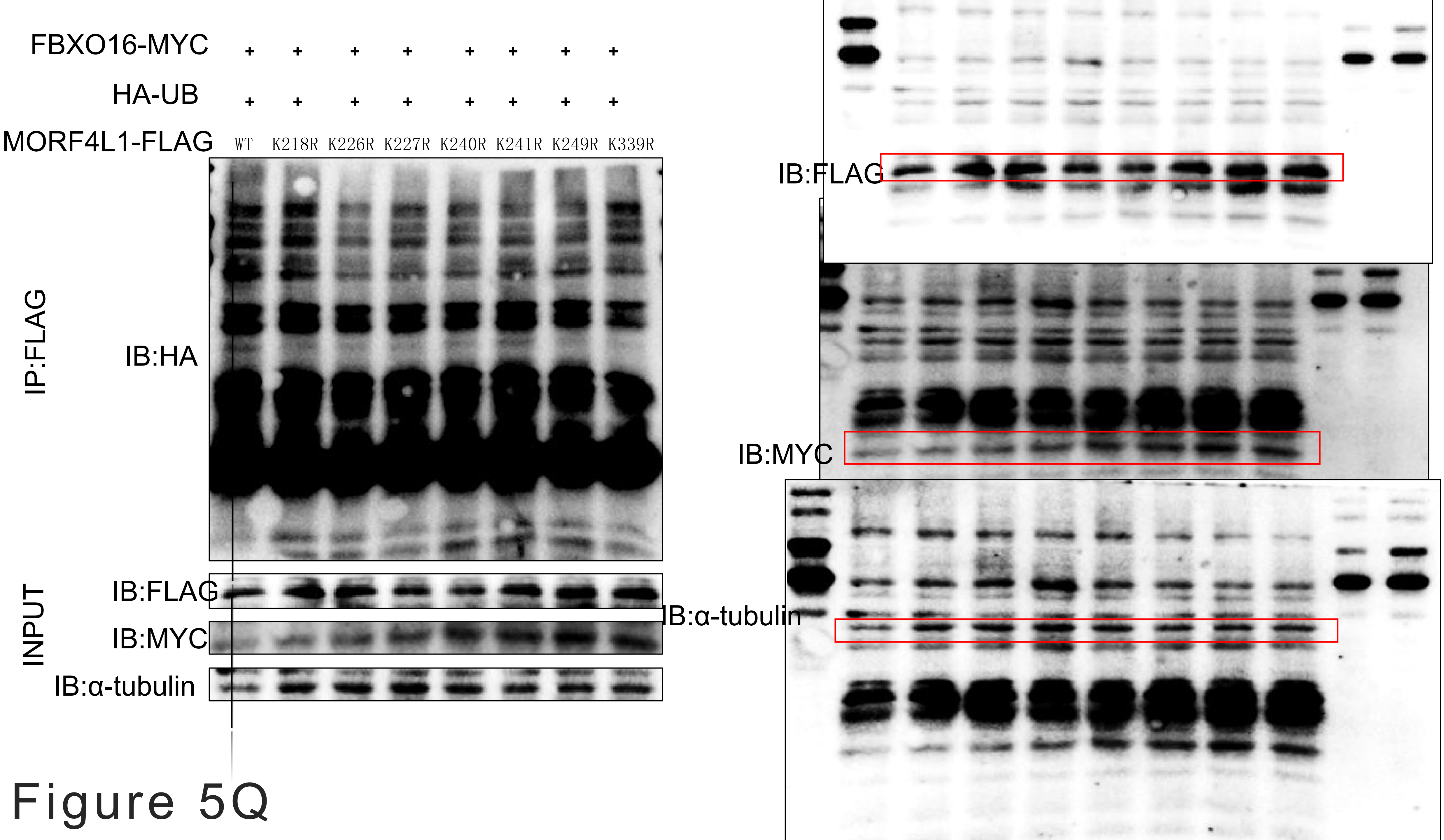
FBXO16-MYC + + + + + + + +
HA-UB + + + + + + + +
MORF4L1-FLAG WT K218R K226R K227R K240R K241R K249R K339R
IP:FLAG
IB:HA
INPUT
IB:FLAG
IB:MYC
IB:α-tubulin
IB:FLAG
IB:MYC
IB:α-tubulin

Figure 5Q

Figure5R

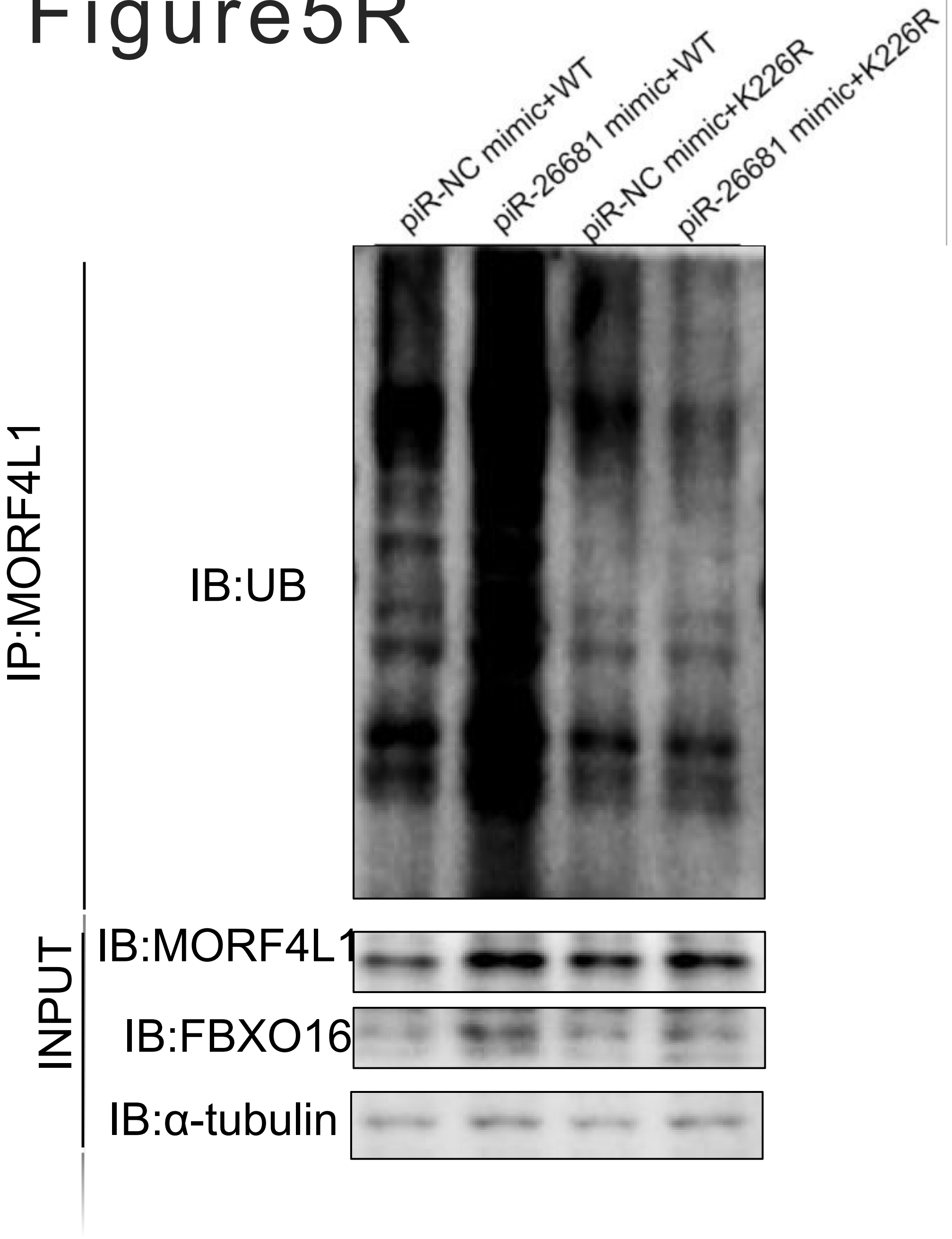

piR-NC mimic+WT
piR-26681 mimic+WT
piR-NC mimic+K226R
piR-26681 mimic+K226R
IP:MORF4L1
IB:UB
INPUT
IB:MORF4L1
IB:FBXO16
IB:α-tubulin

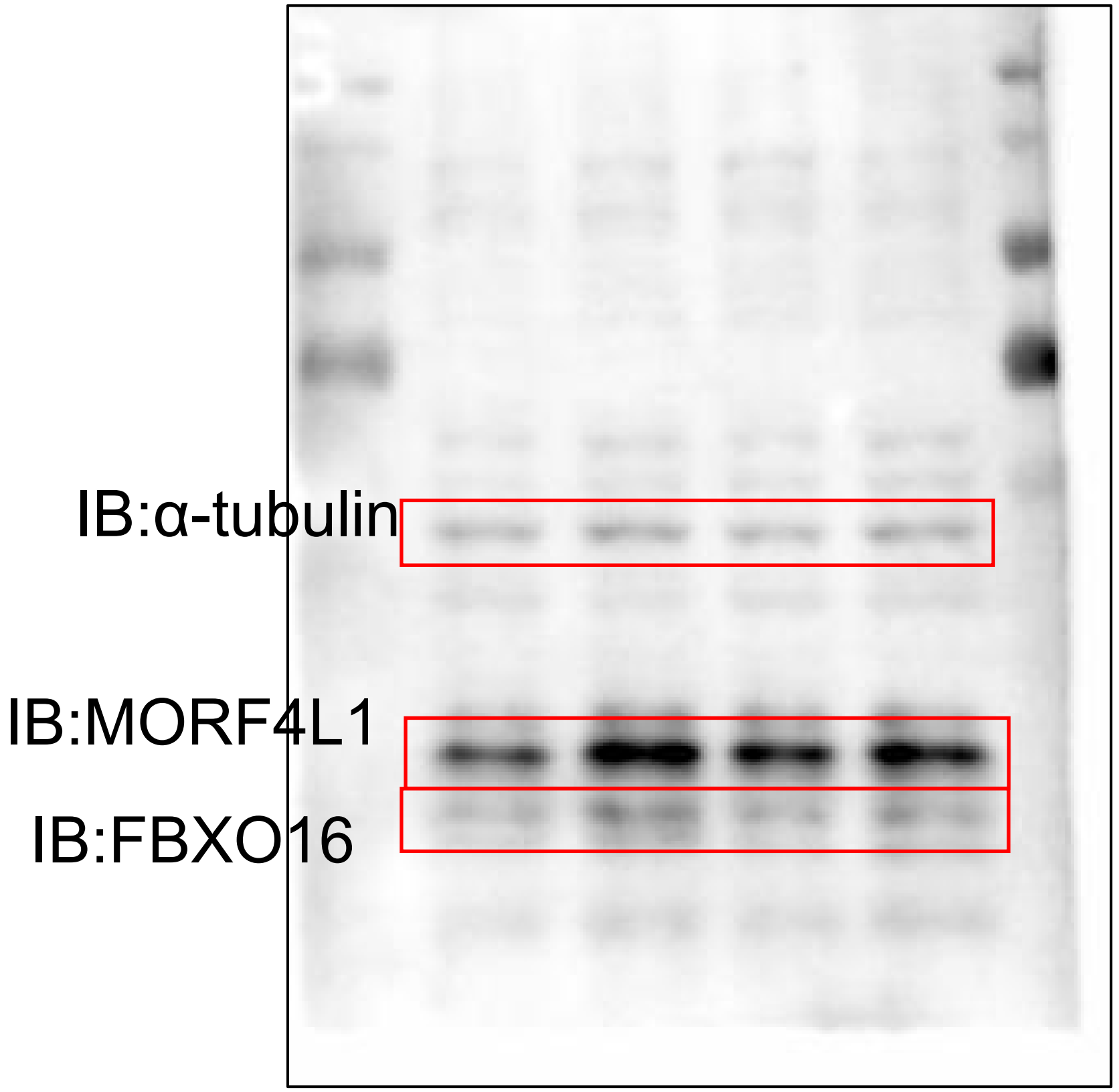

IB:α-tubulin
IB:MORF4L1
IB:FBXO16

Figure 7F

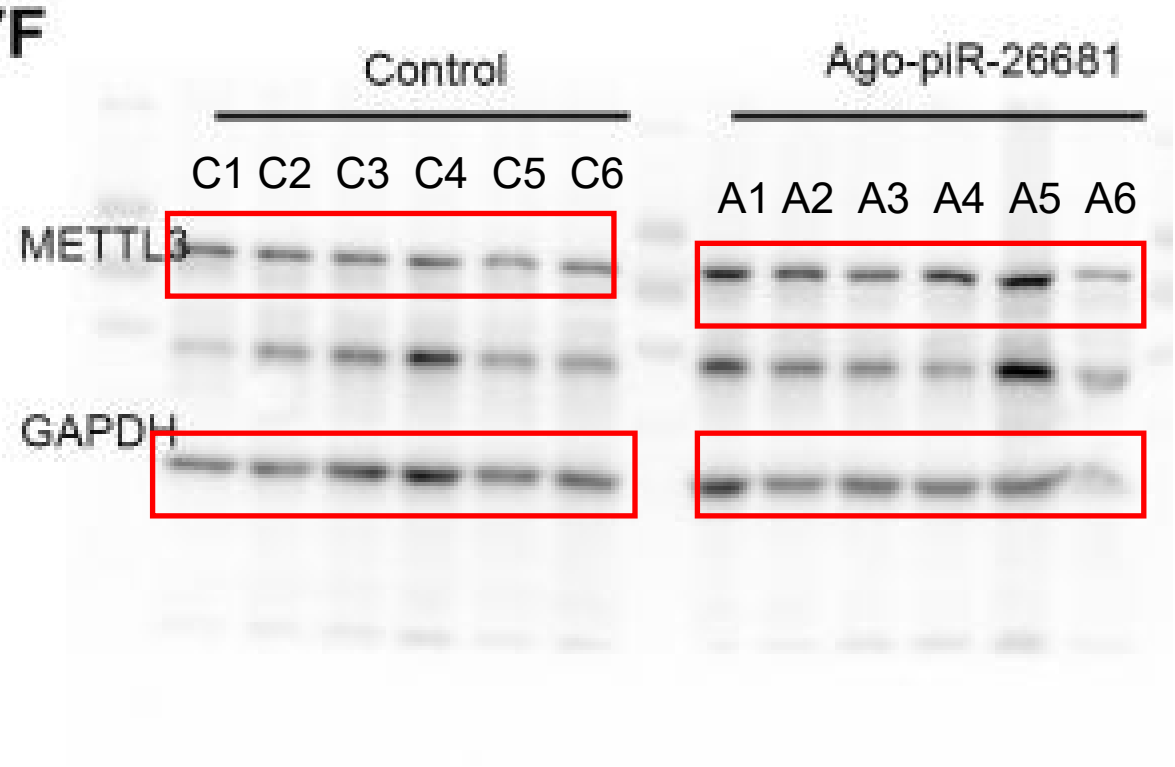

Figure 7G

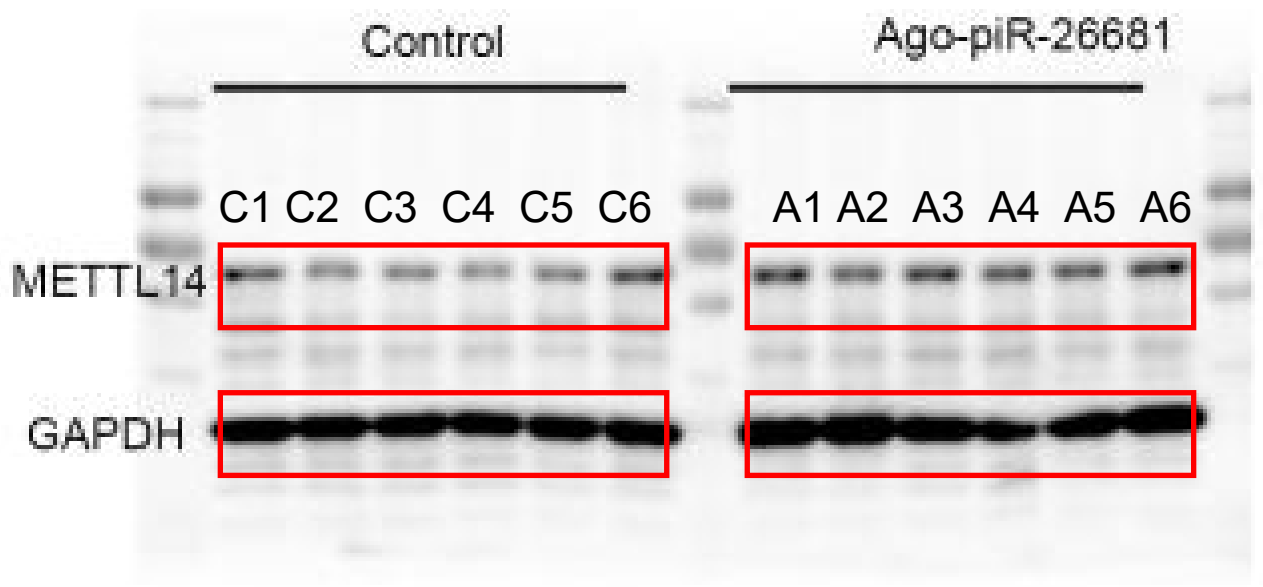

Figure 7H

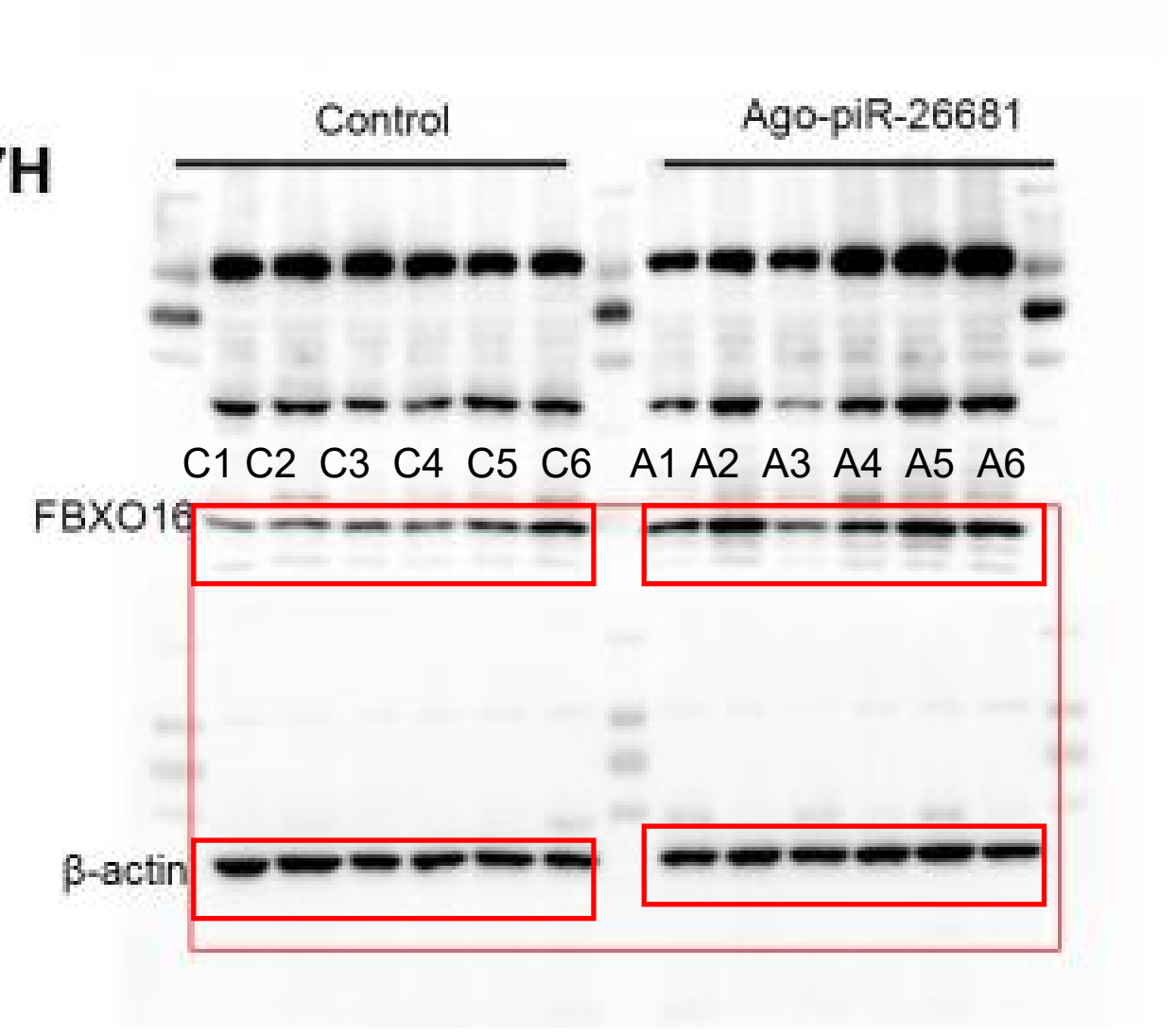

FigureS7A

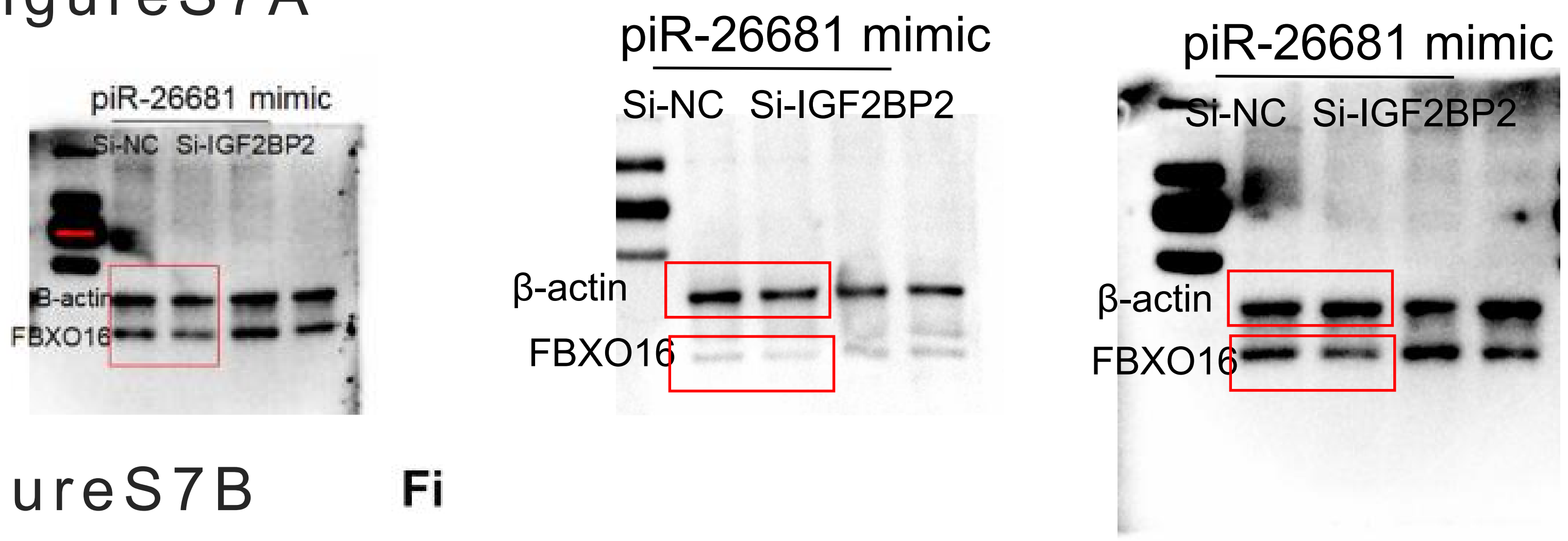

FigureS7B

Fi

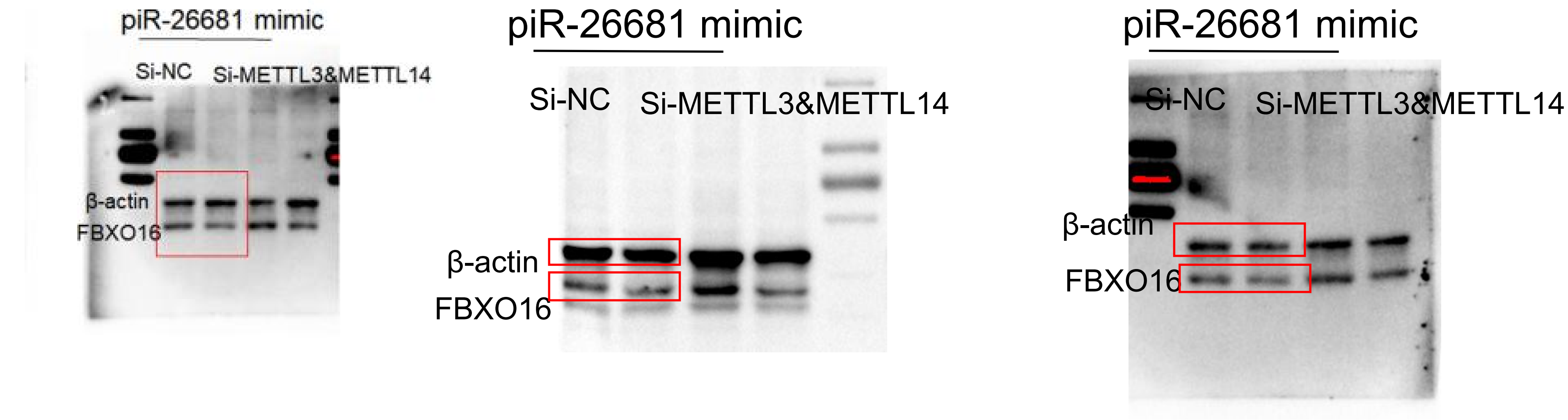

FigureS7C

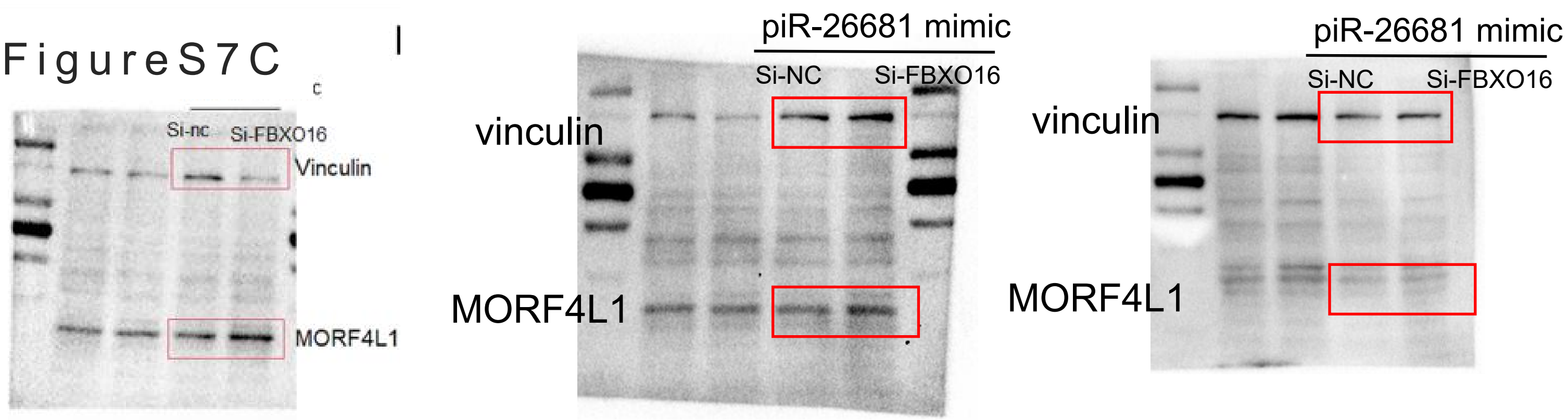

FigureS7D

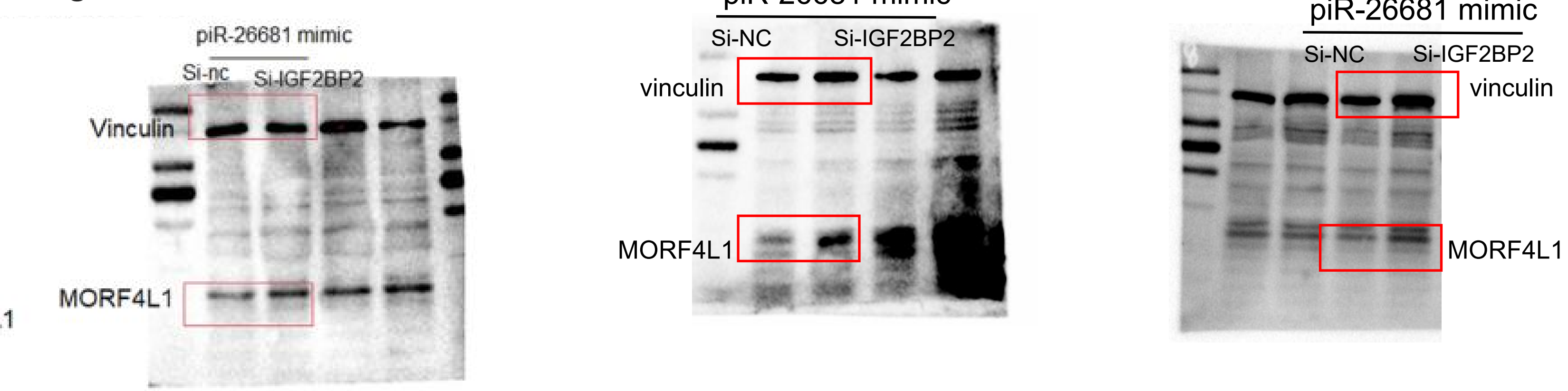

FigureS7E

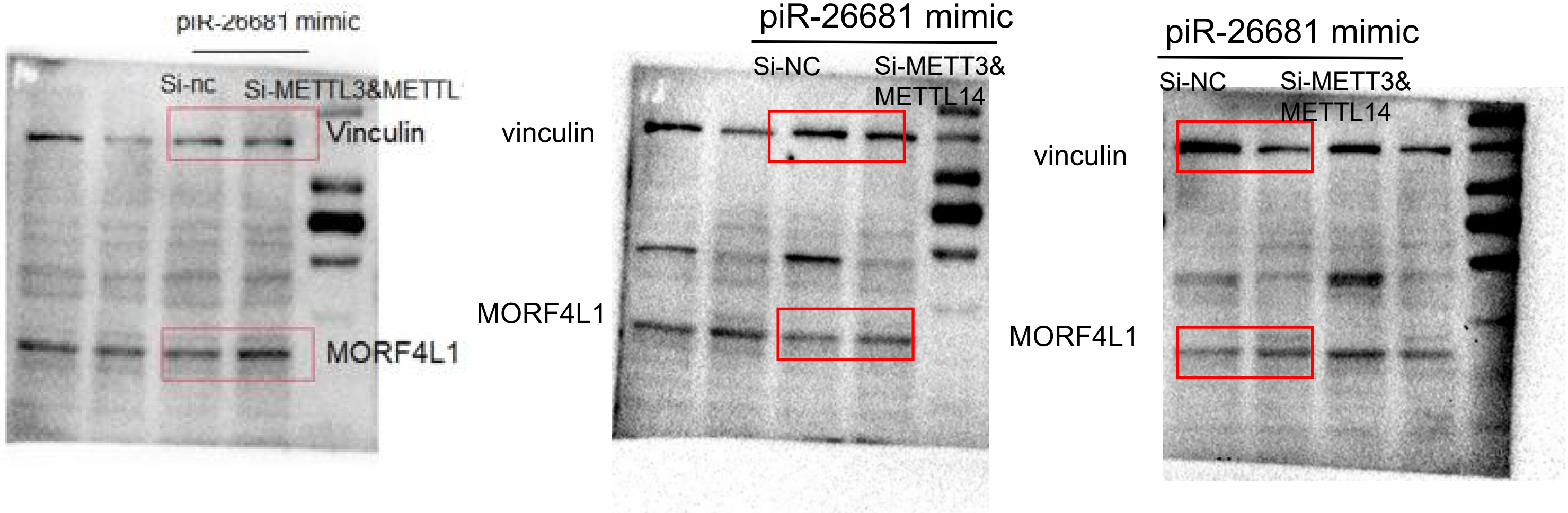

Supplement: Supplementary file 1 [file 11658_2026_969_MOESM2_ESM.pdf]
