# Supplementary material for: piR-26681 suppresses ovarian cancer progression by enhancing METTL3/METTL14-mediated m6A modification of FBXO16 and impairing DNA repair via MORF4L1 degradation
Source: Cell Mol Biol Lett. 2026 Jun 24;31:143. doi: 10.1186/s11658-026-00969-x (PMC13560453; doi:10.1186/s11658-026-00969-x)
Supplement: Supplementary file 2 [file 11658_2026_969_MOESM1_ESM.doc]

**piR-26681 Suppresses Ovarian Cancer Progression by Enhancing METTL3/METTL14-Mediated m6A Modification of FBXO16 and Impairing DNA Repair via MORF4L1 Degradation**

Jie-Lin Wang^#1^, Qing-Hua Wu^#1^, Jing Yuan ^1^, Hai-Juan Bao^1^, Xiang-Chun Huang^1^, Jia-Chen Cheng^1^, Yang Zhao*****^2^, Shuo Chen*****^1^

^1^ Department of Obstetrics and Gynecology, Department of Gynecologic Oncology Research Office; Guangzhou Key Laboratory of Targeted Therapy for Gynecologic Oncology; Guangdong Provincial Key Laboratory of Major Obstetric Diseases; Guangdong Provincial Clinical Research Center for Obstetrics and Gynecology; Guangdong-Hong Kong-Macao Greater Bay Area Higher Education Joint Laboratory of Maternal-Fetal Medicine; The Third Affiliated Hospital, Guangzhou Medical University, Guangzhou, China.

^2^ **Cancer Hospital of Dalian University of Technology, Cancer Hospital of China Medical University, Liaoning Cancer Hospital & Institute, Shenyang, Liaoning , China**

*****Correspondence to: Shuo Chen; Tel/Fax: +86 159 9883 8006; E-mail address: chenshuo077003@163.com. Yang Zhao; Tel/Fax: +86 139 4014 0056; E-mail address: yida.zhaoyang@163.com

Address: No.63 Duobao Road, Liwan District, Guangzhou City, Guangdong Province, P.R. China

#These authors contributed equally to this work.

**Materials and methods**

**Cell Proliferation Assay**
Cells were seeded in 96-well plates at a density of 2,000 cells per well in 100 µL complete medium. Twenty-four hours later, cells were transiently transfected with the indicated constructs. At 0, 24, 48, and 72 hours post-transfection, 10 µL of CCK-8 reagent (Yeasen, Shanghai, China) was added to each well. After a 2-hour incubation at 37 °C, absorbance was measured at 450 nm. All experiments were performed in triplicate.

**EDU Assay**

For the EdU assay using the BeyoClick™ EdU-488 Kit, cells were incubated with 2× EdU working solution for 2 hours, then fixed with 4% paraformaldehyde and permeabilized with 0.3% Triton X-100. After the Click reaction with the prepared solution, cells were stained with Hoechst 33342 for nuclear visualization. Fluorescence was observed under a microscope.

**Transwell Assay**

Matrigel matrix (1:10 dilution, 30 μL; BD Biosciences, San Jose, CA, USA) was added to the upper chamber of the Transwell apparatus and incubated at 37°C for 4 hours to allow coagulation. Then, 200 μL of serum-free medium containing 50,000 cells was added to the upper chamber, and 600 μL of complete medium was placed in the lower chamber. After 48 hours of incubation, cells were washed, fixed with 4% paraformaldehyde, and stained with crystal violet for 15 minutes to quantify the extent of cell invasion.

**Wound Healing Assay**

8× 10^5 cells were seeded in 6-well plates and grown to 80% confluence. A vertical wound was created using a 200-μL pipette tip, and the cells were washed three times with PBS before being cultured in serum-free medium (2 mL). Images of the wound were captured at 0, 24, and 48 hours, and ImageJ was used to measure the wound area.

**Apoptosis Assay**
For apoptosis analysis, 3.0 × 10^5 cells were plated per well in 6-well plates and transfected the following day. Forty-eight hours post-transfection, cells were harvested, washed twice with PBS, and resuspended in 100 µL of Annexin V binding buffer containing 5 µL Annexin V-FITC and 5 µL propidium iodide (BD Pharmingen, San Diego, CA, USA). Samples were incubated in the dark for 15 minutes, followed by addition of 400 µL binding buffer, and analyzed by flow cytometry. Each experiment was repeated three times.

**Colony Formation Assay**
Cells were seeded in 6-well plates at 1,000 cells per well in 2 mL medium and allowed to adhere overnight before transient transfection. After 8–10 days, colonies were gently washed with PBS, fixed in 4% paraformaldehyde for 15 minutes at room temperature, washed again with PBS, and stained with 0.1% crystal violet (Solarbio, Beijing, China). Excess stain was removed by gentle rinsing under running water, and plates were air-dried before colony counting. Experiments were conducted in triplicate.

**Real-time quantitative reverse-transcription polymerase chain reaction (qRT-PCR)**

TRIZOL was used to extract the total RNA from cells or tissues, followed by the generation of endogenous complementary DNA using a Fast Reverse Transcription Kit. The cDNA served as a template for qRT-PCR, which was performed using the QuantStudio 3 (Thermo). The relative expression of the gene was calculated by comparing the cycle threshold (Ct) value of the target gene with that of the control gene (U6) using the 2−ΔCt method. The probe sequence is available in the supplementary file.

**RNA Immunoprecipitation (RIP)**

Briefly, cells were lysed using RIP lysis buffer, where an RNA inhibitor and protease were contained. The supernatant from the cell lysate was collected and incubated with antibody-conjugated beads at four °C overnight. Subsequently, the binding complexes were washed, purified, and analyzed by qRT-PCR.

**Fluorescence In Situ Hybridization (FISH)**

The reverse probe for piR-26681 was synthesized by Sangon Biotech. Subsequently, Fluorescent In Situ Hybridization (FISH) experiments were conducted using a FISH Kit from RIBOBIO, following the manufacturer’s protocol.

**RNA stability test**

After transfection with an overexpressed piR-26681 plasmid, CAOV3 and OVCAR3 cells were treated with 5 μg/ml actinomycin D (ActD, CAS# S8964, Selleck). Total RNA was extracted from the cells at 0, 2, 4, 6, and 8 hours after the addition of actinomycin D. The extracted RNA was then analyzed using qRT-PCR.

**COIP and Western-blot analysis**

Proteins were extracted using RIPA lysate (Beyotime) or Nuclear and Cytoplasmic Protein Extraction Kit (Beyotime), and protein concentrations were measured with a BCA protein assay kit (Beyotime). For Co-IP, the proteins from CAOV3 cells and antibody or normal rabbit immunoglobulin (Ig)G (negative control) and A+G beads (Beyotime, China) were incubated at 4°for 12h. Protein (30ug) was pipetted into an SDS-PAGE gel and subsequently transferred to a PVDF membrane. The membranes were blocked and incubated with primary antibodies, such as METTL3, METTL14, MORF4L1, Cyclin D1, C-Myc, E-cadherin, N-cadherin, vimentin, beta-actin, and GAPDH (Proteintect) and FBXO16 (Zenbio). After three washes with TBST, the membranes were incubated with appropriate anti-rabbit/mouse secondary antibodies at room temperature for 2 hours. Imaging was performed using equipment from BIORAD.

**Cycloheximide Chase and MG132 Assays**

After transfection, CAOV3 or OVCAR3 cells from different groups were treated with cycloheximide (CHX; Selleck, S7418; 100 μg/mL) for various time points (0, 12, 24, 36, and 48 hours) or with MG132 (Selleck, S2619; 20 μM) for 6 hours. Cell lysates were then prepared, and western blot analysis was performed using specific antibodies to assess protein degradation.

**Ubiquitin determination**

To determine ubiquitin levels, cells were transfected with piR-26681 mimic and treated with MG132 for 6 hours. Cell lysates were then incubated with anti-MORF4L1 antibody at 4°C overnight. The next steps were the same as for Co-IP. Western blotting was performed to detect the changes in ubiquitin levels.

**RNA pulldown assay**

Bioprobes, including BIO-NC (UUGUACYACACAAAAGUACUG) and PiR-26681, were synthesized by Sangon company. Subsequently, pulldown experiments were performed using an RNA pulldown kit from BersinBio, following the company’s protocol.

**MeRIP-seq**

Total RNA was extracted using TRIzol. Subsequently, 10μg total RNA was fragmented into 100-200 nt RNA fragments using 10X RNA Fragmentation Buffer (100 mM Tris-HCl, 100 mM ZnCl_2_ in nuclease-free H_2_O). The fragmentation reaction was stopped by adding 10X EDTA (0.5M EDTA). Methylated RNA immunoprecipitation was performed using an EpiTM m6A immunoprecipitation kit (Epibiotek, R1804). The m6A-enriched RNA was purified using TRIzol™ Reagent (Invitrogen, 15596018). Libraries for sequencing were prepared using the Smart-seq method. Both the input samples without IP and the m6A IP samples underwent 150-bp, paired-end sequencing on an Illumina NovaSeq 6000 sequencer.

**m6A dot blot**

Poly(A) RNA was purified from total cellular RNA using the Dynabeads mRNA Purification Kit (ThermoFisher). The purified Poly(A) RNA was denatured at 95°C for 3 minutes and subsequently spotted onto a nylon membrane. The membrane was then subjected to UV crosslinking at 254 nm, 0.12 J/cm^2^. After blocking in PBS with Tween 20 containing 5% bovine skimmed milk for 1 hour, the membrane was incubated with an anti-m6A antibody (2 ug/ml, Proteintech) followed by an anti-mouse IgG secondary antibody. Visualization was performed using a BIO-RAD Imaging System. Subsequently, the same poly(A) RNA was again spotted on a separate section of the membrane, which was then stained with 0.02% methylene blue in 0.3 M sodium acetate (pH 5.2) for 1 hour. The membrane was washed with TBST for 1 hour to remove excess stain.

**Comet experiment**

Drug-treated cells were digested with trypsin, resuspended by centrifugation, and prepared into a 1 × 106 cell suspension in PBS. The comet assay was performed per the instructions of the reagent manufacturer (Comet Assay Kit, Beyotime). After completing the single-cell gel electrophoresis, the cells were neutralized, stained, washed, and observed under a fluorescence microscope and photographed.

**In vivo tumor xenograft model**

In a separate experiment, OVCAR3 cells (5 × 10^6^ in 150 µL FBS-free medium) were injected subcutaneously into 5-week-old female BALB/c-nu mice (n = 12). After the tumor volume reached 100mm^3^, the mice were randomly divided into two treatment groups (6 mice per group). Each group received subcutaneous injections of either 2nmol/each AgopiR-26681 (treatment group) or an equal volume of PBS (control group) 4 times. Tumor volumes were measured and calculated as: Tumor volume (mm3) = 0.5 × length × width^2^. Tumor growth curves are presented as mean ± SEM, and statistical significance between groups was analyzed using two-way ANOVA. Tumor diameters were measured with a Vernier caliper. The mice were euthanized after 18 days, and the tumors were excised. The excised tumors were further subjected to immunohistochemistry (IHC) and western blot experiments. The above operations were completed by experienced experimental technicians and were double-blind. The conduct of these animal experiments adhered to the Guide for the Care and Use of Laboratory Animals, published by the National Institute of Health. The experimental protocol was approved by the Animal Experimentation Ethics Committee of Guangdong Medical Laboratory Animal Center (Approval No. B202208-8). Work has been reported in accordance with the ARRIVE guidelines.

**Data analysis**

GraphPad Prism (version 9.5.0) was used for graphics generation and data analysis. The Kaplan–Meier curve was drawn by https://www.bioinformatics.com.cn, an online platform for data analysis and visualization. All experiments were performed at least three times. The data were expressed as the mean ± standard error of the mean. Statistical analysis was conducted using a two-sample t-test or 2way ANOVA, and a *p*-value of < 0.05 was considered to indicate a significant difference.

**Supplementary Table.1** Primer sequences used for PCR assays

|  | **Forward primer** | **Reverse primer** |
| --- | --- | --- |
| **METTL3** | 5`-TTGTCTCCAACCTTCCGTAGT-3 | 5`-CCAGATCAGAGAGGTGGTGTAG-3 |
| **METTL14** | 5`-AGTGCCGACAGCATTGGTG-3` | 5`-GGAGCAGAGGTATCATAGGAAGC-3` |
| **FBXO16** | 5`- AGCTTTTCGGTCCTCTTCCTC-3` | 5’-GGGTCACGGTTGTCTAGGT-3’ |
| **IGF2BP2** | 5`-AGTGGAATTGCATGGGAAAATCA-3' | 5`-CAACGGCGGTTTCTGTGTC-3' |
| **U6** | 5`-CTCGCTTCGGCAGCACA-3' | 5'-AACGCTTCACGAATTTGCGT-3' |

The above primers are synthesized by BGI Genome company. MiDETECT A TrackTM piR-hsa-26681 Forward Primer and miDETECT A Track U6 primers are synthesized by Ribobio Company.

**Supplementary Table. 2** The oligonucleotides used in this study

|  | **Sequences** |
| --- | --- |
| **SiRNA METTL3** | CAAGTATGTTCACTATGAA |
| **SiRNA METTL14** | CAACTACAATGCAGAAACA |
| **SiRNA IGF2BP2** | GAACGAACTGCAGAACTTA |
| **SiRNA FBXO16** | GTCGAAAGCTTCAAGAGAA |
| **Mimic piR-26681** | GAGAAAGCTCACAAGAACTGCTAACTCAC |

All of the siRNAs are synthesized by Ribobio Company.

## Table S3. Univariate Cox regression analysis of the association between piR-26681 expression and patient survival.

| **Variable** | **coef** | **exp(coef)** | **se(coef)** | **z** | **P value** |
| --- | --- | --- | --- | --- | --- |
| piR-26681 | -0.4452 | 0.6407 | 0.1571 | -2.834 | 0.0046 ** |

## Table S4. Multivariate Cox regression analysis evaluating the prognostic value of piR-26681 expression and clinical variables in ovarian cancer patients.

| **Variable** | **coef** | **exp(coef)** | **se(coef)** | **z** | **P value** |
| --- | --- | --- | --- | --- | --- |
| piR-26681 | -0.172461 | 0.841592 | 0.159314 | -1.083 | 0.279 |
| Age | -0.003769 | 0.996239 | 0.017311 | -0.218 | 0.828 |
| Stage | 1.047438 | 2.850339 | 0.229494 | 4.564 | 5.02e-06 *** |

**Supplementary Figures**

**Figure S1. Screening and functional evaluation of additional candidate piRNAs identified from piRNA sequencing.**

**(A–D)** Kaplan–Meier analyses of progression-free survival (PFS) in ovarian cancer patients stratified by expression levels of four candidate piRNAs (piR-27133, piR-923, piR-9419, and piR-21622). **(E)** Stage-adjusted Kaplan–Meier survival analysis of piR-26681 expression in ovarian cancer patients. **(F)** Relative expression of piR-26681 in normal ovarian tissues and ovarian cancer tissues in an independent validation cohort. **(G)** Expression levels of piR-26681 in early-stage (FIGO I–II) and late-stage (FIGO III–IV) ovarian cancer samples in the validation cohort. **(H)** Distribution of piR-26681 expression across different pathological subtypes of ovarian cancer. **(I–J)** Cell viability assays in CAOV3 (I) and OVCAR3 (J) cells transfected with piR-993 or piR-19620 mimics compared with the negative control (piR-NC mimic), showing no significant effects on cell proliferation. **(K–L)** Relative mRNA expression levels of METTL3 (K) and METTL14 (L) following transfection with piR-993 or piR-19620 mimics, showing no significant changes. **(M)** EdU incorporation assays assessing cell proliferation in CAOV3 and OVCAR3 cells after transfection with piR-993 or piR-19620 mimics. **(N)** Wound healing assays evaluating the migration ability of CAOV3 and OVCAR3 cells transfected with piR-993 or piR-19620 mimics. **(O)** Transwell invasion assays assessing invasive capacity in CAOV3 and OVCAR3 cells after piR-993 or piR-19620 overexpression. **(P)** Flow cytometric analysis of apoptosis using Annexin V-FITC/PI staining in CAOV3 and OVCAR3 cells following transfection with piR-993 or piR-19620 mimics. Data are presented as mean ± SEM. *p < 0.05, **p < 0.01, ***p < 0.001.

**Figure S2. piR-26681 Expression in CAOV3 and OVCAR3 Cells after Overexpression and Downregulation**

**(A)** Relative piR-26681 expression levels in CAOV3 cells after overexpression with piR-26681 mimic or control (piR-NC mimic), measured by qPCR.
**(B)** Relative piR-26681 expression levels in OVCAR3 cells after overexpression with piR-26681 mimic or control (piR-NC mimic), measured by qPCR.
**(C)** Relative piR-26681 expression levels in CAOV3 cells after downregulation with sh-piR-26681 or scramble control, measured by qPCR.
**(D)** Relative piR-26681 expression levels in OVCAR3 cells after downregulation with sh-piR-26681 or scramble control, measured by qPCR.

**Figure S3. Functional analysis of piR-26681 in normal ovarian epithelial cells (IOSE-80).**

**(A)** CCK-8 assay showing cell viability of IOSE-80 cells transfected with piR-26681 mimic or sh-piR-26681 at the indicated time points. **(B)** EdU staining assay evaluating cell proliferation in IOSE-80 cells after transfection with piR-26681 mimic or sh-piR-26681. **(C)** Wound healing assay assessing the migratory ability of IOSE-80 cells following piR-26681 overexpression or knockdown. **(D)** Flow cytometry analysis of apoptosis in IOSE-80 cells using Annexin V/PI staining. **(E)** Transwell invasion assay evaluating the invasive ability of IOSE-80 cells after piR-26681 overexpression or knockdown. Data are presented as mean ± SEM. *p < 0.05, **p < 0.01, ***p < 0.001.

**Figure S4. PIWI protein involvement in the interaction between piR-26681 and the METTL3–METTL14 complex. (A) RNA pull-down assay using biotin-labeled piR-26681 or control RNA (Bio-NC) followed by immunoblotting for PIWIL1–PIWIL4.**

**(B) Co-IP using anti-METTL3 antibody followed by immunoblotting for PIWIL3 and METTL3.**

**(C)Co-IP using anti-METTL14 antibody followed by immunoblotting for PIWIL3 and METTL14.**

**Figure S5. Efficiency of METTL3 and METTL14 Knockdown in Ovarian Cancer Cells**

**(A)** Relative expression of METTL14 after individual and combined knockdown of METTL3 and METTL14 in ovarian cancer cells, as assessed by qPCR.
**(B)** Relative expression of METTL3 after individual and combined knockdown of METTL3 and METTL14 in ovarian cancer cells, as assessed by qPCR.

**Figure S6. Functional enrichment analysis of m⁶A-modified and differentially expressed genes.**

**(A–C)** GO enrichment analysis of transcripts with differential m⁶A peaks identified by MeRIP-seq, including biological process (BP), cellular component (CC), and molecular function (MF). **(D)** KEGG pathway enrichment analysis of transcripts with differential m⁶A peaks.**(E–G)** GO enrichment analysis of differentially expressed genes identified by RNA-seq, including BP, CC, and MF. **(H)** KEGG pathway enrichment analysis of differentially expressed genes.

**Figure S7. Effects of FBXO16, IGF2BP2, and METTL3/METTL14 Knockdown on piR-26681-Mediated Protein Expression**

**(A)** Western blot analysis of FBXO16 protein levels in CAOV3 cells overexpressing piR-26681 with IGF2BP2 knockdown.
**(B)** Western blot analysis of FBXO16 protein levels in CAOV3 cells overexpressing piR-26681 with METTL3/METTL14 knockdown.
**(C)** Western blot analysis of MORF4L1 protein levels in CAOV3 cells overexpressing piR-26681 with FBXO16 knockdown.
**(D)** Western blot analysis of MORF4L1 protein levels in CAOV3 cells overexpressing piR-26681 with IGF2BP2 knockdown.
**(E)** Western blot analysis of MORF4L1 protein levels in CAOV3 cells overexpressing piR-26681 with METTL3/METTL14 knockdown.

**Figure S8. Evaluation of potential F-box proteins interacting with MORF4L1.**

**(A)** Protein–protein interaction network of MORF4L1 retrieved from the BioGRID database. Among F-box proteins, FBXO16 and FBXO32 were predicted as potential interacting partners.**(B)** Endogenous Co-IP assay in CAOV3 cells showing MORF4L1 IP followed by IB analysis of FBXO32.

**Figure S9. Functional assays evaluating the role of FBXO16 and MORF4L1 ubiquitination in ovarian cancer cells.**

**(A)** Cell proliferation measured by CCK-8 assays in cells treated with piR-26681 overexpression or FBXO16 overexpression. **(B)** DNA synthesis assessed by EdU incorporation assays under the same conditions as in **(A). (C)** Wound-healing assays performed under the same conditions as in (A). **(D)** Transwell invasion assays performed under the same conditions as in (A). **(E)** Flow cytometry analysis of apoptosis (Annexin V-FITC/PI staining) under the same conditions as in (A).**(F)** Quantification of functional assays shown in (B–E). **(G)** Comet assays performed under the same conditions as in (A). **(H)** IF staining of γ-H2AX foci under the same conditions as in (A). **(I) C**ell proliferation measured by CCK-8 assays in cells treated with piR-26681 knockdown and/or FBXO16 overexpression. **(J)** EdU incorporation assays under the same conditions as in (I). **(K)** Wound-healing assays under the same conditions as in (I). **(L)** Transwell invasion assays under the same conditions as in (I). **(M)** Flow cytometry analysis of apoptosis (Annexin V-FITC/PI staining) under the same conditions as in (I). **(N)** Quantification of functional assays shown in (I–M). **(O)** EdU incorporation assays in cells expressing WT or K226R MORF4L1 with or without piR-26681 overexpression. **(P)** Wound-healing assays in cells expressing WT or K226R MORF4L1 with or without piR-26681 overexpression. **(Q)** Transwell invasion assays in cells expressing WT or K226R MORF4L1 with or without piR-26681 overexpression. **(R)** Flow cytometry analysis of apoptosis (Annexin V-FITC/PI staining) in cells expressing WT or K226R MORF4L1 with or without piR-26681 overexpression.

**Figure S10. Immunohistochemical Analysis of METTL3, METTL14, and FBXO16 Protein Levels in Tumor Tissues from Xenograft Models.** (A) Immunohistochemical staining of METTL3 in control and Ago-piR-26681-treated tumors. (B) Immunohistochemical staining of METTL14 in control and Ago-piR-26681-treated tumors. (C) Immunohistochemical staining of FBXO16 in control and Ago-piR-26681-treated tumors.

**Cell identification**
